# Benefits and harms adopted by health economic assessments evaluating antenatal and newborn screening programmes in OECD countries: A systematic review of 336 articles and reports

**DOI:** 10.1016/j.socscimed.2022.115428

**Published:** 2022-12

**Authors:** May Ee Png, Miaoqing Yang, Sian Taylor-Phillips, Svetlana Ratushnyak, Nia Roberts, Ashley White, Lisa Hinton, Felicity Boardman, Abigail McNiven, Jane Fisher, Baskaran Thilaganathan, Sam Oddie, Anne-Marie Slowther, Jenny Shilton Osborne, Stavros Petrou, Oliver Rivero-Arias

**Affiliations:** a Nuffield Department of Primary Care Health Sciences, University of Oxford, Oxford, UK; b National Perinatal Epidemiology Unit, Nuffield Department of Population Health, University of Oxford, Oxford, UK; c Bodleian Health Care Libraries, University of Oxford, Oxford, UK; d Warwick Medical School, University of Warwick, Coventry, UK; e THIS Institute, University of Cambridge, Cambridge, UK; f Antenatal Results and Choices, UK; g St George's University Hospital NHS Foundation Trust, London, UK; h Bradford Institute for Health Research, Bradford Children's Research, Bradford, UK

**Keywords:** Benefits, Harms, Screening programme, Antenatal, Newborn, Economic evaluation, Cost-effectiveness analysis

## Abstract

**Background:**

Health economic assessments are used to determine whether the resources needed to generate net benefit from a screening programme, driven by multiple complex benefits and harms, are justifiable. We systematically identified the benefits and harms incorporated within economic assessments evaluating antenatal and newborn screening programmes.

**Methods:**

For this systematic review and thematic analysis, we searched the published and grey literature from January 2000 to January 2021. Studies that included an economic evaluation of an antenatal or newborn screening programme in an OECD country were eligible. We identified benefits and harms using an integrative descriptive analysis, and illustrated a thematic framework. (Systematic review registration PROSPERO, CRD42020165236).

**Findings:**

The searches identified 52,244 articles and reports and 336 (242 antenatal and 95 newborn) were included. Eighty-six subthemes grouped into seven themes were identified: 1) diagnosis of screened for condition, 2) life years and health status adjustments, 3) treatment, 4) long-term costs, 5) overdiagnosis, 6) pregnancy loss, and 7) spillover effects on family members. Diagnosis of screened for condition (115 studies, 47.5%), life-years and health status adjustments (90 studies, 37.2%) and treatment (88 studies, 36.4%) accounted for most of the benefits and harms evaluating antenatal screening. The same themes accounted for most of the benefits and harms included in studies assessing newborn screening. Overdiagnosis and spillover effects tended to be ignored.

**Interpretation:**

Our proposed framework can be used to guide the development of future health economic assessments evaluating antenatal and newborn screening programmes, to prevent exclusion of important potential benefits and harms.

## Highlights

• Health economic assessments are used to determine value for money of antenatal and newborn screening programmes.
• Systematic review conducted to identify benefits and harms adopted in health economic assessments.
• There is no consistency in the adoption of benefits and harms of screening programmes in these studies.
• A thematic framework is proposed to assist researchers with the identification of benefits and harms.

## Author contributors

MEP: Data curation, Formal analysis, Investigation, Methodology, Project administration, Resources, Visualisation, Writing – original draft, Writing – review & editing, MY: Formal analysis, Investigation, Methodology, Writing – review & editing, ST-P: Conceptualization, Formal analysis, Funding acquisition, Writing – review & editing, SR: Methodology, Writing – review & editing, NR: Resources, Writing – review & editing. AW: Writing – review & editing. LH: Conceptualization, Funding acquisition, Writing – review & editing, FB: Conceptualization, Funding acquisition, Writing – review & editing. AMcN: Writing – review & editing. JF: Conceptualization, Funding acquisition, Writing – review & editing, BT: Conceptualization, Funding acquisition, Methodology, Writing – review & editing, SO: Conceptualization, Funding acquisition, Methodology, Writing – review & editing, A-MS: Conceptualization, Funding acquisition, Writing – review & editing, JSO: Project administration, Resources, Writing – review & editing, SP: Conceptualization, Formal analysis, Funding acquisition, Investigation, Methodology, Project administration, Resources, Supervision, Writing – review & editing, OR-A: Conceptualization, Formal analysis, Funding acquisition, Investigation, Methodology, Project administration, Resources, Supervision, Visualisation, Writing – original draft, Writing – review & editing

## 1 Introduction

Antenatal and newborn screening programmes can facilitate earlier detection and diagnosis of health conditions, enabling timely care and treatment for pregnant women and their newborns. A successful screening programme maximises benefits and minimises harms to all the relevant stakeholders affected by screening (Raffle and JM, 2019). Antenatal screening aims to identify conditions early, allowing pregnant women and their partners to make informed choices about pregnancy management, including termination. The identification of conditions is also crucial in newborn screening for the appropriate care and implementation of relevant interventions to maximise survival and quality of life of the newborn.

Antenatal and newborn screening programmes are associated with many benefits and harms. Harms of screening associated with false positive and false negative results include unnecessary additional resources to conduct further investigations, adverse psychological and physical effects and legal claims, as well as decreased trust and confidence in the health care system (Petticrew et al., 2000). In antenatal screening, when a decision to continue a pregnancy is made after a true positive result, a potential screening benefit is the time it offers expectant parents to prepare for the birth of a child with a clinical condition. An informed decision to terminate a pregnancy can also follow a true positive result, but this can lead to long-lasting psychosocial sequelae for women and their partners, affecting their quality of life and their future pregnancy choices (Davies et al., 2005; Fuller et al., 2021; Kaimal et al., 2015; Korenromp et al., 2005; Kuppermann et al., 2004, 2016; Woolf-King et al., 2017). The use of genome-wide sequencing for newborn screening presents an opportunity to identify and treat or prevent severe health conditions, but could cause overdiagnosis, overtreatment and greater uncertainty if not assessed properly (Friedman et al., 2017; Phillips et al., 2018).

Population screening programmes are evaluated by national screening committees using independent evidence-based recommendations relevant to the jurisdiction that may adopt the programme. The United Kingdom National Screening Committee (UK NSC) and the United States Preventive Services Task Force (USPSTF) are examples of screening committees using such approaches. The recommendation to adopt a screening programme on a national scale is based on the premise that the benefits associated with screening outweigh the harms once implemented. In the UK, for instance, the UK NSC requires evidence of these benefits and harms, and data demonstrating that the screening programme represents value for money. The latter is determined using a health economic assessment confirming that the additional costs of implementing a screening programme are justified by the additional benefits achieved. Decision-analytic based economic assessments can account for the abovementioned benefits and harms through outcome measures (e.g. quality-adjusted life years [QALYs]), model inputs and the structure of the decision-problem (Caro et al., 2012). There is established guidance on best practices for economic modelling for screening programmes in general (Weinstein et al., 2003), but this guidance does not address the challenges of how to incorporate the breadth of potentially relevant benefits and harms into a single assessment, and does not specifically focus on antenatal and newborn screening. Guidance in this area, therefore, remains limited (Karnon et al., 2007). Failure to incorporate all relevant benefits and harms when assessing the cost-effectiveness of antenatal and newborn screening programmes may lead screening committees to make decisions based on sub-optimal evidence.

In this study, we report the first systematic review of the benefits and harms of antenatal and newborn screening adopted by different types of health economic assessments in the published and grey literature.

## 2 Methods

### 2.1 Overview

We used the PRISMA 2020 checklist (Page et al., 2021) when reporting the methods and results of this systematic review. The review protocol was registered with PROSPERO (CRD42020165236) and published on January 13, 2020 (Png et al., 2021). This review is based on data available from secondary sources and published materials; hence, ethics committee approval or written informed consent was not required.

### 2.2 Search strategy and selection criteria

The PICOS (Population, Intervention, Comparator, Outcome and Study design) framework was used to develop the study eligibility criteria (Table 1) and applied to the literature searches. The search strategy (Supplementary Table 1) was developed in collaboration with an information specialist (NR) and limited to studies published from January 1, 2000 onwards. A simplified search strategy based on the Cochrane guidelines was applied to the grey literature search (Higgins et al., 2019). Translation of the simplified search terms for non-English websites was performed by professional translators.

**Table 1:** Inclusion and exclusion criteria for identification of relevant studies.

Characteristics	Inclusion criteria	Exclusion criteria
Population	Pregnant women Newborns	Anyone other than pregnant women or newborns Studies on animals Not conducted in an OECD member country)^a^
Intervention	Antenatal or newborn screening programme^b^	Pre-conception screening No screening programme
Comparator	No screening or specific form(s) of screening other than experimental intervention(s), as defined by specific conditions	
Outcome	Benefits and harms of antenatal or newborn screening that have been identified, measured and valued by economic assessments	
Study design	Economic evaluation design: • Cost-effectiveness analysis • Cost-utility analysis • Cost-benefit analysis • Cost-consequences analysis • Cost-minimisation analysis Economic framework that incorporates cost-effectiveness evidence or economic notion of value (e.g., Multi-Criteria Decision Analysis, Programme Budgeting and Marginal Analysis)	Descriptive cost analysis Budget impact analysis Not an economic evaluation Other report types: • Editorial • Letter • Methodological research without applied evidence • Perspective, opinion or commentary • Protocol • Review

a Studies from countries that become OECD members after the title/abstract screening process was completed were not included (last OECD member included was Colombia) (OECD, 2020).

b This includes actual and proposed, e.g., hypothetical screening programmes as well as any aspect of a screening programme (defined as a whole system of activities needed to deliver high quality screening), for example, the performance of screening test.

The published literature was searched using the following electronic bibliographic databases: Medline (OvidSP)[1946-present], Embase (OvidSP)[1974-present], NHS Economic Evaluation Database (via CRDWeb https://www.crd.york.ac.uk/CRDWeb/)[Inception to March 31, 2015], EconLit (Proquest)[1969-present], Science Citation Index, Social Science Citation Index and Conference Proceedings Citation Index – Science (Web of Science Core Collection)[1945-present], CINAHL (EBSCOHost)[1982-present] and PsycINFO (OvidSP)[1806-present]. SCOPUS (Elsevier) was used to run forward and backward citation searches once relevant studies were identified. The academic electronic database search was supplemented by manual reference searching of bibliographies, contacts with experts in the field and author searching. Only studies assessing screening programmes in at least one of the Organization for Economic Co-operation and Development (OECD) countries were included. A full search of the published literature was conducted on January 22, 2021 (Supplementary Table 1). Identified published studies were exported to EndNote version X9 (Clarivate, Philadelphia, United States of America, 2019) for deduplication and then imported into Covidence software (Veritas Health Innovation, n.d.) for screening. Screening of titles and abstracts, and subsequently of full-text articles identified in the published literature, was performed using the eligibility criteria by two independent reviewers (MEP and MY). In addition, MEP assessed 100% and SR 10% of the grey literature during the screening process. Disagreements related to the screening process between the two reviewers were resolved by discussion and involvement of other members of the review team (OR-A and SP) if necessary. For non-English language papers, Google Translate (Google, Mountain View, California, USA) was used to translate relevant documents.

The list of sources of grey literature searched was informed by a recent systematic review of national policy recommendations on newborn screening that identified around 30 websites of national and regional screening organisations with documentation about antenatal and/or newborn screening recommendations (Taylor-Phillips et al., 2018). This was widened to cover websites reported by the Health Grey Matters checklist and those for national and regional screening organisations, health technology assessment agencies, paediatrics organisations, and obstetrics and gynaecology societies in OECD countries, as well as international decision-making bodies, such as the World Health Organization, the European Council, European Commission and the European Observer (CADTH: Canadian Agency for Drugs and Technologies in Health, 2014; Taylor-Phillips et al., 2018). A customised web-scraping tool that used the Google search engine was built using Python to directly query the stated websites in January/February 2021 using English search terms and translated search terms for non-English websites, as well as to automate the data extraction processes. The grey literature that was identified was exported to Microsoft Excel for deduplication. We refer to ‘articles’ and ‘reports’ in our presentation of results when referring to the published and grey literature, respectively.

### 2.3 Data analysis

A data extraction sheet, which was piloted and refined using ten randomly selected studies identified in the academic electronic databases, was created following recommendations from the Cochrane Handbook for Systematic Reviews of Interventions (Higgins et al., 2019). As we had anticipated a large number of articles to data extract and after consulting our Independent Oversight Committee members and information specialist (NR), a selection of the papers/reports was extracted independently by two health economists (MEP and MY), followed by a reconciliation process. High level of agreement between MEP and MY was observed after assessing 10% of the papers/reports during this reconciliation process. The rest of the published literature was singly extracted by the two reviewers (MEP and MY). The grey literature was extracted by one reviewer (MEP). Any disagreement was resolved by discussion and involvement of other members of the review team (OR-A and SP) if necessary. The list of variables extracted from each article and report included at the final stage of the review process was finalised following the piloting and refinement of the data extraction sheet.

The data extraction form consisted of two parts:

1) A section that contained items from the Consolidated Health Economic Evaluation Reporting Standards (CHEERS) checklist (Husereau et al., 2013), modified where applicable to align with our research focus. This included: bibliographic details; condition(s) screened; approaches for measuring and valuing health outcomes; the journal impact factor quartile during the year that the article was published, obtained from Clarivate Analytics and SCImago as an indicator of interest in the topic by journal editors; whether the authors made any policy recommendation based on their economic evaluation evidence; and whether the authors might have had any potential conflicts of interest in promoting their screening programme or mechanism (defined as a study that was funded by an industry sponsor, unless it was an unrestricted grant, and at least one of the authors being clearly employed by the industry sponsor).
2) A bespoke form created by the research team to extract benefits and harms adopted by economic assessments evaluating screening programmes. This form was created *de novo* as we could not find any previous examples in the published literature. A description of the consequences as reported by authors by screening test outcome (i.e. true positives, false positives, true negatives and false negatives) and source (i.e. probability, cost or outcome) was captured and categorised as either a benefit or a harm. We also recorded the stage of the disease pathway at which the screening test was administered and the phase(s) of the screening programme using categorisations from recent guidance (Raffle and JM, 2019). The form also recorded whether the structure of decision-analytical models had been reported, and any consequences associated with treatment if included.

Since only aggregated data and no effect sizes were sought, we did not assess the risk of bias or conduct a formal meta-analysis. Instead, the reporting quality of articles and reports (excluding conference abstracts) was assessed using the CHEERS checklist (Husereau et al., 2013). The items include title and abstract; background and objectives; target population and subgroups; setting and location; study perspective; comparators; time horizon; discount rate; choice of health outcomes; measurement of effectiveness; measurement and valuation of preference-based outcomes; estimation of resources and costs; currency, price date and conversion; choice of model; assumptions; analytical methods; study parameters; incremental costs and outcomes; characterizing of uncertainty; characterizing of heterogeneity; study findings, limitations, generalizability, and current knowledge; source of funding; and conflicts of interest. These items were considered as ‘satisfied’ if reported in full or ‘not satisfied’ if not reported or partially reported.

We used the information captured in the bespoke form to create a framework of benefits and harms adopted by health economic assessments using a process of grouping themes into categories derived from information extracted about consequences in the bespoke form (Morse, 2008). An integrative descriptive analysis (Sandelowski, 2010) of the collated themes within each category was then conducted, resulting in a thematic framework of benefits and harms consisting of a primary theme and up to four levels of subtheme(s). In the first step, the description of consequences was categorised into specific themes by ST-P. This pool of themes was the starting point of an iterative process where members of the study team (ST-P, MEP, OR-A, and SP) merged, separated and refined the wording of themes and subthemes. During this step, special attention was paid to avoid overlapping of broad themes. The iterative process was maintained until consensus was reached among the study team (ST-P, MEP, OR-A, and SP). Articles and reports were categorised into themes and subtheme(s) according to the condition and screening type. Bar charts were generated to illustrate the thematic framework across and by medical condition(s).

### Role of the funding source

The funder of the study had no role in study design, data collection, data analysis, data interpretation, or writing of the report.

## 3 Results

### 3.1 Systematic review

**Fig. 1:**
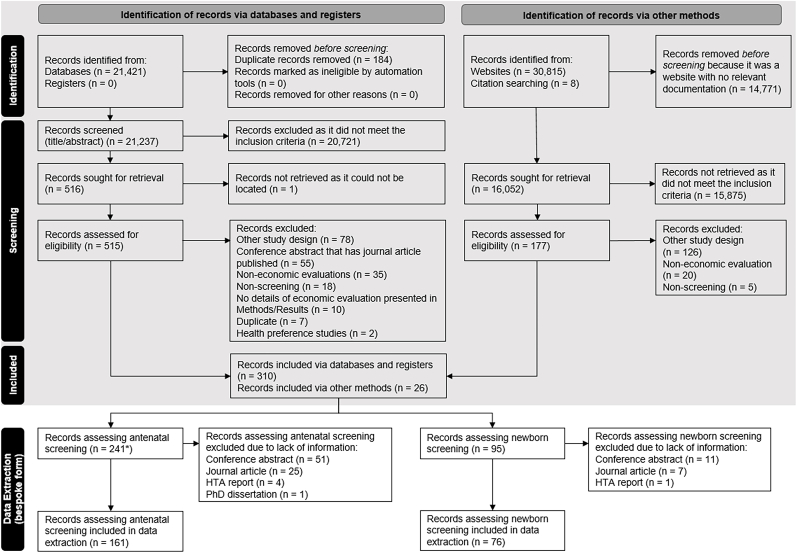
Modified PRISMA flow diagram of articles and reports selection and data extraction process. CAPTION:* One HTA report included two separate economic evaluations that were separated into two different reports, resulting in 242 outputs from the 241 records.

We identified 52,244 articles and reports from the searches of the published and grey literature. Among the 16,052 records that were sought for retrieval based on identification of records via other methods (i.e. grey literature), 7464 records were non-English (46.5%). Thirty-nine studies of the non-English records were assessed for eligibility with five subsequently included in the data extraction phase. A total of 336 records (310 articles and 26 reports) were included in the systematic review. One HTA report included two separate economic evaluations that were separated into two different reports, resulting in 337 outputs. Study selection and reasons for exclusion as well as data extraction of the ancillary form are summarised in the PRISMA diagram (Fig. 1). The list of studies excluded is summarised in Supplementary Table 2. The number of articles and reports are presented in Supplementary Fig. 1 by year of publication and screening type; no general trend was observed between the year of publication and screening type. Characteristics of the included articles and reports are presented in Table 2. The majority of those included were journal articles (228, 67.7%) with almost half conducted in the United States of America (109, 32.2%) or the United Kingdom (43, 12.7%). For the majority of articles and reports, further information was required to determine if the authors had potential conflicts of interest (221, 65.6%). Furthermore, the authors did not make any recommendation about the adoption of the screening programme based on the economic evidence generated for the majority of the articles and reports (273, 81.0%). The majority of the articles were published in top quartile medical journals (i.e. quartile one; 129, 38.3%).

**Table 2:** Characteristics of articles and reports.

	Articles and reports assessing antenatal screening (%) (n = 242)	Articles and reports assessing newborn screening (%) (n = 95)	Total articles and reports (%) (n = 337)
Publication type			
Journal article	156 (64.5)	72 (75.8)	228 (67.7)
Conference abstract	61 (25.2)	12 (12.6)	73 (21.7)
HTA report	24 (9.9)	11 (11.6)	35 (10.4)
PhD dissertation	1 (0.4)	0 (0)	1 (0.3)
Country of screening programme^a^			
United States of America	82 (33.7)	27 (28.4)	109 (32.2)
United Kingdom	32 (13.2)	11 (11.6)	43 (12.7)
Canada	17 (7)	15 (15.8)	32 (9.5)
The Netherlands	12 (4.9)	9 (9.5)	21 (6.2)
France	8 (3.3)	4 (4.2)	12 (3.6)
Australia	9 (3.7)	3 (3.2)	12 (3.6)
Spain	6 (2.5)	4 (4.2)	10 (3.0)
Colombia	3 (1.2)	3 (3.2)	6 (1.8)
Austria	3 (1.2)	1 (1.1)	4 (1.2)
Israel	4 (1.6)	0 (0)	4 (1.2)
Italy	3 (1.2)	1 (1.1)	4 (1.2)
Germany	1 (0.4)	2 (2.1)	3 (0.9)
Belgium	1 (0.4)	2 (2.1)	3 (0.9)
Finland	1 (0.4)	1 (1.1)	2 (0.6)
Sweden	1 (0.4)	3 (3.2)	4 (1.2)
Chile	1 (0.4)	0 (0)	1 (0.3)
Czech Republic	1 (0.4)	0 (0)	1 (0.3)
Denmark	1 (0.4)	0 (0)	1 (0.3)
Ireland	2 (0.8)	0 (0)	2 (0.6)
Japan	0 (0)	1 (1.1)	1 (0.3)
New Zealand	1 (0.4)	1 (1.1)	2 (0.6)
Norway	2 (0.8)	0 (0)	2 (0.6)
Switzerland	1 (0.4)	0 (0)	1 (0.3)
Not stated	51 (21)	7 (7.4)	58 (17.2)
Potential conflicts of interest			
No	70 (28.9)	38 (40)	108 (32.0)
Yes	7 (2.9)	1 (1.1)	8 (2.4)
More information needed to classify	165 (68.2)	56 (58.9)	221 (65.6)
Policy recommendation			
No	194 (80.2)	79 (83.2)	273 (81.0)
Yes	48 (19.8)	16 (16.8)	64 (19.0)
Journal impact factor quartile (articles only)			
First quartile of medical journals	36 (10.7)	93 (27.6)	129 (38.3)
Second quartile of medical journals	17 (5.0)	26 (7.7)	43 (12.8)
Third quartile of medical journals	11 (3.3)	27 (8.0)	38 (11.3)
Fourth quartile of medical journals	3 (0.9)	7 (2.1)	10 (3.0)
Not available	5 (1.5)	3 (0.9)	8 (2.4)

a Will not total up to 100% as some articles and reports included more than one country. HTA: Health technology assessment (refers to reports generated by screening and HTA organisations).

The characteristics of screening programmes and populations in the included articles and reports are summarised in Supplementary Table 3. There were 173 (71.5%) studies on antenatal screening and 63 (66.3%) studies on newborn screening that did not state the setting of the screening. The majority of the antenatal studies did not state the women's gestational stage at the time of screening (168, 65.4%). The majority of the studies were targeted at the general population of pregnant women (197, 57.1%) or infants (91, 26.4%). Many studies were investigations at the symptomless stage with pathologically definable change present (303, 89.9%) or involved all phases of the screening process (162, 48.1%).

The medical conditions investigated are summarised in Supplementary Table 4. Genetic conditions and infectious diseases (153, 63.2%) were the main areas covered by the articles and reports assessing antenatal screening. Metabolic and structural conditions (57, 60.0%) were the main areas covered by health economic assessments evaluating newborn screening programmes.

The methodological characteristics of the health economic assessments are summarised in Table 3. Decision analytical models were employed in 272 (81.0%) of the articles and reports whilst 117 (43.0%) of all articles and reports used a lifetime time horizon. Almost half of them conducted a cost-utility analysis reporting quality-adjusted life years (QALYs) (167, 49.4%). The costing perspective adopted was not stated in 117 (33.7%) articles and reports. Maternal preference-based outcomes (QALYs; disability-adjusted life years (DALYs)) were reported in 94 (72.9%) antenatal screening evaluations whereas infant preference-based outcomes were reported in 34 (89.5%) newborn screening evaluations.

**Table 3:** Health economic assessment characteristics of the articles and reports.

	Articles and reports assessing antenatal screening (%) (n = 242)	Articles and reports assessing newborn screening (%) (n = 95)
Study design		
Individual patient-level data analysis	12 (5.0)	6 (6.4)
Cohort	10 (4.1)	5 (5.3)
Cross-sectional	0 (0)	1 (1.1)
Randomised controlled trial	2 (0.8)	0 (0)
Decision-analytical model	200 (82.6)	72 (76.6)
Decision tree	90 (37.2)	39 (41.5)
Decision tree and Markov model	9 (3.7)	6 (6.4)
Discrete event simulation model	1 (0.4)	1 (1.1)
Markov model	10 (4.1)	15 (16.0)
Model type not specified	83 (34.3)	8 (8.5)
Patient-level simulation model	7 (2.9)	3 (3.2)
Other	2 (0.8)	3 (3.2)
Not stated	28 (11.6)	13 (13.8)
Type of economic evaluation^a^		
Cost-benefit analysis	17 (7.0)	5 (5.3)
Cost-consequences analysis	7 (2.9)	3 (3.2)
Cost-effectiveness analysis	87 (36.0)	47 (50.0)
Cost-minimisation analysis	2 (0.8)	3 (3.2)
Cost-utility analysis	129 (53.3)	38 (40.4)
Perspective of costs^a^		
Health system or payer	107 (43.5)	53 (52.5)
Societal	44 (17.9)	25 (24.8)
Not applicable^b^	0 (0)	1 (1.0)
Not stated	95 (38.6)	22 (21.8)
Time horizon of decision-analytical model		
Up to delivery	9 (4.5)	1 (1.4)
Up to 1 year	26 (13.0)	6 (8.3)
Between 1 year to specific time horizon excluding lifetime	8 (4.0)	14 (19.4)
Lifetime	80 (40.0)	37 (51.4)
Not stated	77 (38.5)	14 (19.4)
Sources to inform health benefits		
Primary data collection	21 (9.7)	13 (14.1)
Evidence-synthesis of secondary data	167 (77.3)	62 (67.4)
Combination of primary and secondary data	28 (13.0)	16 (17.4)
Expert opinion only	0 (0)	1 (1.1)
Reporting of preference-based outcomes in cost-utility analysis		
Maternal QALYs/DALYs	94 (72.9)	4 (10.5)
Infant QALYs/DALYs	19 (14.7)	34 (89.5)
Maternal and infant QALYs/DALYs	16 (12.4)	0 (0)

a Will not total up to 100% as some articles and reports reported more than one category.

b This is a multiple-criteria decision analysis.

Reporting quality assessed using the CHEERS checklist was heterogeneous among the 264 full length articles and reports (as summarised in Supplementary Table 5). The top five items not satisfied among the studies for both antenatal and newborn screening programmes were ‘Abstract’ (229, 86.7%), ‘Time horizon’ (220, 83.3%), ‘Choice of model’ (208, 78.8%), ‘Discount rate’ (183, 69.3 and ‘Study funding, limitation, generalizability, and current knowledge’ (182, 68.9%). The top five items satisfied among the studies for both antenatal and newborn screening programmes were ‘Background and objectives' (264, 100%), ‘Target population and subgroups' (264, 100%), ‘Choice of health outcomes' (263, 99.6%), ‘Measurement of effectiveness' (260, 98.5%), and ‘Estimate resources and cost’ (247, 93.6%).

### 3.2 Thematic synthesis

We identified 86 unique descriptions of consequences across all articles and reports from our bespoke extraction form. Our thematic analysis resulted in seven core themes of benefits and harms with each core theme including up to four levels of subtheme(s). An abridged version of the thematic framework with a description of each theme and key examples is presented in Table 4 with the full version up to subtheme level 4 presented in Supplementary Table 6.

**Table 4:** Thematic framework of benefits and harms adopted by health economic assessments evaluating antenatal and newborn screening programmes (abridged version).

Theme no.	Theme	Description	Key selected examples
1	Diagnosis of screened for condition	Related to the process of identifying a condition through screening. For example, cases diagnosed or missed, confirmatory tests (necessary and unnecessary), reduction in infants born with condition through effective treatment, or pregnancy termination	Infants born with condition Confirmatory test and additional tests to reach diagnosis of screened for condition Cases missed at screening Cases diagnosed at screening Screened for condition related complications Additional screening of partners Additional testing to reach diagnosis in the absence of screening (links to diagnostic odyssey)
2	Life years and health status adjustments	Impact of identifying a condition on the health of women, infants and other family members and included, for example, standard health measures such as QALYs, disability-adjusted life years (DALYs), life years, or impact of anxiety on parents after a false positive result	Infant life years post birth (including QALYs) Maternal life years (including QALYs) Parental QALYs Psychological (anxiety/disutility from false positive results, genetic variants of unclear penetrance, or knowledge of disease)
3	Treatment	Caused by harms of adverse reactions, unnecessary interventions and antibiotic resistance, or benefits of adverse complications averted due to timely interventions	Comparison of earlier treatment after screen detection and later after symptomatic detection Additional healthcare post-diagnosis Hospital stay Missed due to false negative Prevention of screened for condition (infectious) Psychological (counselling about screening/confirmatory test/genetic diagnosis) Screened for condition related treatment/management treatment related harm (disutility/anxiety/adverse reaction/antibiotic resistance)< Unnecessary due to false positive
4	Long-term cost associated with screened for condition	Impact on long-term healthcare and non-healthcare costs related to identifying a condition through screening.	Direct healthcare cost Direct non-healthcare cost (education/social care/caregiving) Productivity gains Societal cost
5	Overdiagnosis	Impact on costs and consequences of detecting a condition that would never develop into symptomatic disease.	QALY decrement Unnecessary test/treatment
6	Pregnancy loss	Caused by treatment or an invasive diagnostic procedure, or an informed decision of termination after a true positive result	Spontaneous Termination (of unaffected fetus due to false positive test result/prevent downstream adverse maternal outcomes/psychological consequences) Treatment/test related
7	Spillover effects	Benefits or harms to parents and other relevant stakeholders from the child's diagnosis	Benefits or harms to parents from child's diagnosis with genetic condition, through knowledge of their own genetic status

**Fig. 2:**
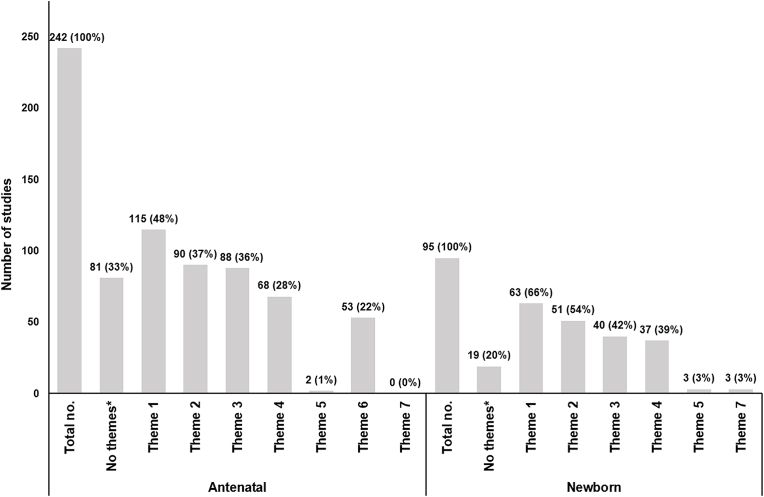
Benefits and harms adopted by health economic assessments evaluating antenatal screening programmes and newborn screening programmes using thematic framework. CAPTION: Theme 1: Diagnosis of screened for condition, Theme 2: Life years and health status adjustments, Theme 3: Treatment, Theme 4: Long-term cost associated with screened for condition, Theme 5: Overdiagnosis, Theme 6: Pregnancy loss, Theme 7: Spillover effects. * 51 out of the 81 (63.0%) antenatal screening evaluations and 11 out of the 19 (57.9%) newborn screening evaluations with no themes were conference abstracts. Remaining studies provided limited information to capture benefits and harms in the bespoke form.

The benefits and harms incorporated within health economic assessments are presented in Fig. 2 by screening type using the thematic framework. Limited information about benefits and harms could be extracted from 81 (33.5%) out of the 242 antenatal screening evaluations and 19 (20.0%) out of the 95 newborn screening evaluations to inform our bespoke form. These included 51 out of the 81 (63.0%) antenatal screening evaluations and 11 out of the 19 (57.9%) newborn screening evaluations described in conference abstracts. Across all conditions in antenatal screening in Fig. 2 (n = 242), 115 (47.5%) incorporated benefits and harms related to the diagnosis of screened for condition (theme 1). Ninety (37.2%) of evaluations included benefits and harms related to life-years and health status adjustments (theme 2). Eighty-eight (36.4%) of the antenatal screening evaluations included benefits and harms associated with treatment (theme 3). In general, for antenatal screening, benefits and harms associated with the long-term costs of screened for conditions (theme 4) was adopted in 68 (28.1%) of the evaluations. Only 21 out of the 242 (8.7%) antenatal screening evaluations incorporated benefits and harms from all of themes 1 to 4. Newborn screening, as shown in Fig. 2, had 63 (66.3%) studies that incorporated benefits and harms related to the diagnosis of screened for condition (theme 1). Fifty-one (53.7%) evaluations included life-years and health status adjustment related benefits and harms (theme 2). Forty (42.1%) of the antenatal screening evaluations included benefits and harms associated with treatment (theme 3). Benefits and harms associated with the long-term costs of screened for conditions (theme 4) were only adopted in 37 (38.9%) of the evaluations. Only 17 out of the 95 (17.9%) newborn screening evaluations adopted benefits and harms from all of themes 1 to 4. Benefits and harms related to overdiagnosis (5, 1.5%) and spillover effects (1, 0.3%) were largely absent from the studies.

Supplementary Table 7 summarises the benefits and harms adopted in the articles and reports for specific conditions. Health economic assessments evaluating antenatal screening programmes for infectious diseases adopted the broadest spectrum of benefits and harms compared to the other conditions.

## 4 Discussion

To our knowledge, this review is the first of its kind focusing on the adoption of benefits and harms by health economic assessments of antenatal and newborn screening programmes. Previous work has focused on the identification of methodological challenges and the development of good practice guidelines in the conduct of health economic assessments (Cacciatore et al., 2020; Karnon et al., 2007; Langer et al., 2012). However, this is the first systematic review to identify benefits and harms of these studies in OECD countries. Almost half of the included articles were published in first-quartile journals, indicating interest in the topic by high-impact journals. Most of the economic evidence of antenatal screening programmes focused on screening for genetic conditions or infectious diseases, whilst that surrounding newborn screening programmes primarily focused on screening for metabolic or structural conditions.

We found clear evidence that decision-analytic models represent the main vehicle for the conduct of these studies, unsurprisingly given the nature of the evidence synthesis needed. Almost half of the articles and reports used standard health economic measures of QALYs or DALYs to measure the health benefits of the screening programmes. Lifetime horizons are important to understand the costs and consequences of these screening programmes in the long run, but such an approach was adopted by less than half of the studies. Current lack of long-term data to inform this aspect of economic evaluations partly explains this result (Phillips et al., 2018), but it highlights a serious limitation of these studies. It also indicates that these studies did not adhere to recognised methods guidelines for the conduct of economic evaluations for the purposes of assessing the value for money of screening programmes (National Institute for Health and Care Excellence, 2013).

Our thematic analysis summarised a wide range of benefits and harms adopted by these studies and summarised them into seven core themes. There is no consistency on the selection of benefits and harms across and within conditions suggesting that additional guidance is needed in this field. In general, articles and reports assessing antenatal and newborn screening programmes have considered benefits and harms that reflect the processes of identifying a condition in their health economic assessments. This includes, for example, cases correctly identified or missed or the number of unnecessary tests due to false positives. This result is not surprising because benefits and harms associated with the diagnosis of screened for conditions provide the first line of clinical evidence about these programmes and are of key interest to screening organisations. Around half of the articles and reports evaluating newborn screening programmes across all conditions did not consider benefits and harms associated with life years and health status adjustments. Our review also found that benefits and harms identified as important by screening agencies and international health organisations, including overdiagnosis and spillover effects on family members, have rarely been adopted by these economic evaluations (Raffle and JM, 2019; World Health Organization, 2020). In the case of spillover effects, the only relevant subtheme identified was benefits to parents that inform future reproductive decisions from discovering carrier status as a consequence of the child's diagnosis (Shermock et al., 2005). It is difficult to understand why authors have tended to exclude these relevant themes in their economic analyses because such information is rarely reported. In the case of spillover effects, i.e. the impact of a patient's health and wellbeing on family members and informal caregivers, there have been several calls by academics to routinely include these effects in the evaluation of adult and child health interventions, indicating that this is not an issue specific to evaluations of antenatal and newborn screening programmes (Brouwer et al., 2009; Brouwer, 2018). Access to appropriate data sources to inform model parameters, and time and budget constraints are possible reasons for the omission of overdiagnosis and spillover effects in these studies. However, this should be confirmed by future research and guidance on the conduct of these studies in practice.

Authors did not generally refer to “benefits” and “harms” when describing the utilities and dis-utilities included in their evaluations. In addition, what constitutes a benefit or harm depends on the perspective of the particular stakeholder involved in the decision-making. For instance, a reduction in the number of infants born with a condition through pregnancy termination may be seen by some as a societal benefit in economic terms, due to health care savings and reduced societal comorbidity. However, this may well be considered a devastating harm for families who value living with an infant with a condition. Therefore, we had to extract and interpret detailed information about the consequences included in the studies and reports for the thematic analysis from a neutral ethical perspective when categorising benefits and harms together into unique themes. It is worthwhile noting that the terms benefits and harms are commonly used by national screening committees to communicate their decisions about implementation of these programmes (Centers for Disease Control and Prevention, 2021; UK National Screening Committee, 2021). We hope that our work encourages other health economists to think about the potential breadth of benefits and harms that can be captured within standard health metrics, such as QALYs, when designing, presenting and communicating their work.

A key strength of this review includes the focus on a comprehensive set of antenatal and newborn screening programmes across OECD countries. We did not restrict our searches to English-only records to avoid language bias, and did not restrict our searches to the published literature only in order to avoid publication bias. We have also identified a thematic framework of benefits and harms that can act as a starting point for researchers when considering the benefits and harms to be included in their analyses in the future. However, this study has its limitations. We did not dual extract data as currently recommended (Higgins et al., 2019) due to the large amount of information to extract from the final set of included articles and reports, and the timelines allowed to complete the project. For practical purposes and quality assurance, dual data extraction was performed for 10% of the papers after consulting our Independent Oversight Committee and information specialist (NR) using a reconciliation process that ended in a high-level agreement between reviewers. We also re-ran our search strategies up to November 22, 2021 and an additional 18 articles had been published since January 2021. To incorporate this more recent literature, one assessor (MEP) extracted the consequences included in these new articles using our bespoke form, and no new themes of benefits and harms were identified (Supplementary Table 8), demonstrating the robustness of our framework. It is also possible that we have missed important consequences of benefits and harms associated with these types of screening as our thematic framework is informed by already completed studies. There is currently ongoing qualitative work evaluating the spectrum of benefits and harms of importance to stakeholders affected by antenatal and newborn screening, which will inform whether our framework needs expanding or contracting (UK National Institute for Health and Care Research NIHR Funding and Awards, 2022). Therefore, our framework should be used with caution and as a tool to guide discussions during the design of these studies and should not be employed as a checklist.

In conclusion, we have conducted the first systematic review identifying the benefits and harms incorporated into economic evaluations of antenatal and newborn screening programmes. We found that many of these studies did not adhere to the most recent guidance on the conduct of economic evaluations and that many benefits and harms considered important by screening agencies, including overdiagnosis, are often overlooked (UK National Screening Committee, 2021). Our work suggests that there is an immediate need to provide guidance for researchers conducting these types of studies in the future. Our proposed framework of benefits and harms can be used as a starting point to guide the development of health economic assessments evaluating antenatal and newborn screening for specific conditions.

## Funding

This project was funded by the 10.13039/501100000272National Institute for Health Research (UK): 10.13039/501100000664Health Technology Assessment Programme (NIHR127489).SP receives support as a UK National Institute for Health Research (NIHR) Senior Investigator (NF-SI-0616-10103) and from the UK NIHR Applied Research Collaboration Oxford and Thames Valley. ST-P is supported by an NIHR Career Development Fellowship (CDF-2016-09-018). LH is based at the Healthcare Improvement Studies Institute (THIS Institute), University of Cambridge. THIS Institute is supported by the Health Foundation, an independent charity committed to bringing about better health and healthcare for people in the UK.

## UK department of health disclaimer

The views and opinions expressed therein are those of the authors and do not necessarily reflect those of the Health Technology Assessment Programme, NIHR, NHS, or the Department of Health.

## Data sharing

Study data are available on request to the corresponding author.

## Declaration of competing interest

OR-A, JF, BT and FB are members of the Foetal, Maternal and Child Health (FMCH) reference group of the UK National Screening Committee (UK NSC). ST-P is a member of the UK NSC Adult Reference Group. JF and AMS are members of the UK NSC. The remaining authors declare that they have no competing financial interests or personal relationshipts that could have appeared to influence the work reported in this paper.

## Data Availability

Data will be made available on request.
