# Supplementary material for: Benefits and harms adopted by health economic assessments evaluating antenatal and newborn screening programmes in OECD countries: A systematic review of 336 articles and reports
Source: Soc Sci Med. 2022 Dec;314:115428. doi: 10.1016/j.socscimed.2022.115428 (PMC9720154; doi:10.1016/j.socscimed.2022.115428)
Supplement: Multimedia component 1 [file mmc1.docx]

**Benefits and harms adopted in health economic assessments evaluating antenatal and newborn screening programmes in the OECD countries: A systematic review of 336 articles and reports**

**Supplementary Material**

[Supplementary Tables 2](#_Toc106453827)

[Supplementary Table 1. Search strategy for Ovid MEDLINE® Epub Ahead of Print, In-Process & Other Non-Indexed Citations, Ovid MEDLINE® Daily and Ovid MEDLINE® 1946 to present 2](#_Toc106453828)

[Supplementary Table 2: List of studies excluded at full-text screening stage, with brief reasons 10](#_Toc106453829)

[Supplementary Table 3: Characteristics of screening programmes and population in the articles and reports 11](#_Toc106453830)

[Supplementary Table 4: Medical conditions investigated 13](#_Toc106453831)

[Supplementary Table 5: Summary of reporting quality of articles and reports (excluding conference abstracts) assessed using CHEERS checklist 14](#_Toc106453832)

[Supplementary Table 6: Thematic framework of benefits and harms adopted by health economic assessments evaluating antenatal and newborn screening programmes 16](#_Toc106453833)

[Supplementary Table 7: Summary of thematic framework of benefits and harms adopted by health economic assessments evaluating antenatal and newborn screening programmes by conditions 29](#_Toc106453834)

[Supplementary Table 8: Thematic framework of benefits and harms adopted by health economic assessments evaluating antenatal and newborn screening programmes in the refresh search from 1^st^ January 2021 to 22^nd^ November 2021 31](#_Toc106453835)

[Supplementary Figures 34](#_Toc106453836)

[Supplementary Figure 1: Number of articles and reports published from 2000 to January 2021 34](#_Toc106453837)

[References of Supplementary Material 35](#_Toc106453838)

## Supplementary Tables

### Supplementary Table 1. Search strategy for Ovid MEDLINE® Epub Ahead of Print, In-Process & Other Non-Indexed Citations, Ovid MEDLINE® Daily and Ovid MEDLINE® 1946 to present

Search Strategy: A full search of the published literature was conducted on 24^th^ April 2020 with a top-up search conducted on 2^nd^ July 2020 to include the search term ‘perinatal’, and a refresh search on 22^nd^ January 2021.

| **#** | **Query** |
| --- | --- |
| 1 | Ultrasonography, Prenatal/ |
| 2 | exp Prenatal diagnosis/ |
| 3 | (ultrasound* or ultra-sound or ultrasonogra* or ultra-sonogra* or sonogra* or echocardiogra* or nuchal translucen* or amniocentesis or chorionic villus sampl* or cvs or (((noninvasive prenatal or non-invasive prenatal) adj2 (test* or screen*)) or nipt)).ti,ab. |
| 4 | ((fetal or foetal or fetus or foetus or prenat* or pre-nat* or prepart* or pre-part* or antenatal or ante-natal or perinatal or pregnant or pregnancy or trimester?) adj3 (screen* or test* or diagnos* or scan* or structural assessment* or structural survey*)).ti,ab. |
| 5 | screen*.ti. |
| 6 | exp Abortion, Induced/ |
| 7 | ((induced or therap*) adj3 abortion?).ab,kw. or abortion?.ti. |
| 8 | 1 or 2 or 3 or 4 or 5 or 6 or 7 |
| 9 | exp Congenital Abnormalities/ |
| 10 | primary dysautonomias/ or dysautonomia, familial/ or Tay-Sachs Disease/ |
| 11 | Muscular Atrophy, Spinal/ |
| 12 | (dysautonomia? or tay sachs).ti,ab,kw. |
| 13 | (congenital* adj2 (defect? or malformation? or abnormalit* or anomal* or aneuploid*)).ti,ab. |
| 14 | ((fetal or foetal or fetus or foetus) adj2 (defect? or malformation? or abnormalit* or anomal* or aneuploid*)).ti,ab. |
| 15 | ((structural or neural tube?) adj2 (defect? or malformation? or abnormalit* or anomal*)).ti,ab. |
| 16 | ((non-chromosom* or nonchromosom* or chromosom*) adj2 (defect? or malformation? or abnormalit* or anomal*)).ti,ab. |
| 17 | (((down* or patau* or edward*) adj2 syndrome*) or trisomy 13 or trisomy 18 or trisomy 21).ti,ab. |
| 18 | spinal muscular atrophy.ti,ab,kw. |
| 19 | 9 or 10 or 11 or 12 or 13 or 14 or 15 or 16 or 17 or 18 |
| 20 | 8 and 19 |
| 21 | exp Congenital Abnormalities/di, dg |
| 22 | Prenatal Care/ or Perinatal Care/ |
| 23 | (fetal or foetal or fetus or foetus or prenat* or pre-nat* or prepart* or pre-part* or antenatal or ante-natal or perinatal or pregnant or pregnancy or trimester?).ti,ab. |
| 24 | 22 or 23 |
| 25 | 21 and 24 |
| 26 | ((fetal or foetal or fetus or foetus or prenat* or pre-nat* or prepart* or pre-part* or antenatal or ante-natal or perinatal) adj (screen* or test* or diagnos*)).ti. |
| 27 | 20 or 25 or 26 |
| 28 | Diabetes, Gestational/ or exp Hypertension, Pregnancy-Induced/ |
| 29 | (eclampsia or preeclampsia or pregnancy induced hypertension).ti,ab,kw. |
| 30 | ((gestational or pregnan* or maternal) adj2 diabet*).ti,ab,kw. |
| 31 | exp Obstetric Labor, Premature/ or Vasa Previa/ or Placenta Previa/ or Fetal Death/ |
| 32 | ((preterm or premature) adj2 labo?r).ti,ab,kw. |
| 33 | (f?etal death? or stillbirth? or still birth?).ti,ab,kw. |
| 34 | ((placenta or vasa) adj pr?evia).ti,ab,kw. |
| 35 | anemia, hemolytic, congenital/ or exp anemia, sickle cell/ or exp thalassemia/ |
| 36 | (sickle cell or thalass?emia?).ti,ab,kw. |
| 37 | exp Syphilis/ |
| 38 | syphilis.ti,ab,kw. |
| 39 | exp Hepatitis B/ |
| 40 | Hepatitis B virus/ |
| 41 | (hepatitis b or hbv).ti,ab,kw. or hepatitis.ti. |
| 42 | exp HIV/ |
| 43 | exp HIV Infections/ |
| 44 | (hiv or human immunodeficiency virus).ti,ab,kw. |
| 45 | exp Chlamydia Infections/ or exp Chlamydia/ |
| 46 | chlamydia.ti,ab,kw. |
| 47 | exp Cytomegalovirus Infections/ |
| 48 | cytomegalovirus.ti,ab,kw. |
| 49 | exp Streptococcal Infections/ |
| 50 | (group b strep or strep b or (streptococc* adj infection?)).ti,ab,kw. |
| 51 | exp Parvoviridae Infections/ |
| 52 | parovirus.ti,ab,kw. |
| 53 | Rubella/ or Rubella virus/ or Rubella Syndrome, Congenital/ |
| 54 | rubella.ti,ab,kw. |
| 55 | Toxoplasmosis/ or Toxoplasmosis, Congenital/ |
| 56 | toxoplasmosis.ti,ab,kw. |
| 57 | exp Anemia/ |
| 58 | exp Blood Group Antigens/ |
| 59 | exp Thrombophilia/ |
| 60 | thrombophilia?.ti,ab,kw. |
| 61 | an?emia?.ti,ab,kw. |
| 62 | (blood group? or rhd status or rhesus positive or rhesus negative or rhesus status).ti,ab,kw. |
| 63 | exp Urinary Tract Infections/ |
| 64 | ("urinary tract infection*" or "urine infection*" or uti or cystitis or bacteriuria).ti,ab,kw. |
| 65 | Vaginosis, Bacterial/ |
| 66 | vaginosis.ti,ab,kw. |
| 67 | domestic violence/ or spouse abuse/ |
| 68 | ((spous* or intimate partner or domestic) adj2 (violence or abuse)).ti,ab,kw. |
| 69 | or/28-68 |
| 70 | exp pregnancy/ or pregnant women/ |
| 71 | exp fetus/ |
| 72 | (pregnan$ or f?etal or f?etus or FVS).ti,ab. |
| 73 | preconception care/ or prenatal care/ or perinatal care/ |
| 74 | (pregnan* or preconception* or pre-conception* or antenat* or ante-nat* or antepart* or ante-part* or prenat* or pre-nat* or prepart* or pre-part* or perinatal or maternal or mother*).ti,ab. |
| 75 | 70 or 71 or 72 or 73 or 74 |
| 76 | Mass Screening/ |
| 77 | screen*.ti,ab. |
| 78 | exp Population Surveillance/ |
| 79 | Self Report/ |
| 80 | (selfreport* or self-report* or ((oral or tak*) adj3 history)).ti,ab. |
| 81 | exp Hematologic Tests/ or Diagnostic Tests, Routine/ or Serologic Tests/ |
| 82 | ((h?ematolog* or blood or serum or serologic*) adj3 (test* or assay*)).ti,ab. |
| 83 | ((sero* adj5 (test* or screen* or diagnos*)) or (serotest* or seroscreen* or serodiagnos*)).ti,ab. |
| 84 | exp immunoassays/ |
| 85 | Polymerase Chain Reaction/ |
| 86 | (immuno-assay* or immunoassay* or elisa or eia or Fluorescent antibody to membrane antibod* or fama or trfia).ti,ab. |
| 87 | (enzyme linked immunosorbent assay* or elisa or enzyme immunoassay* or eia or recombinant immunoblot assay* or riba).ti,ab. |
| 88 | (polymerase chain reaction or pcr).ti,ab. |
| 89 | (routine adj5 (test* or screen* or diagnos*)).ti,ab. |
| 90 | (test* or diagnos* or assay*).ti. |
| 91 | 76 or 77 or 78 or 79 or 80 or 81 or 82 or 83 or 84 or 85 or 86 or 87 or 88 or 89 or 90 |
| 92 | 69 and 75 and 91 |
| 93 | Prenatal diagnosis/ or maternal serum screening tests/ |
| 94 | ((pregnan* or preconception* or pre-conception* or antenat* or ante-nat* or antepart* or ante-part* or prenat* or pre-nat* or prepart* or pre-part* or perinatal or maternal or mother*) adj5 (screen* or diagnos* or test*)).ti,ab. |
| 95 | 93 or 94 |
| 96 | 69 and 95 |
| 97 | Diabetes, Gestational/di or exp Hypertension, Pregnancy-Induced/di or exp Obstetric Labor, Premature/di or Vasa Previa/di or Placenta Previa/di or Fetal Death/di or anemia, hemolytic, congenital/di or exp anemia, sickle cell/di or exp thalassemia/di |
| 98 | exp Syphilis/di or exp Hepatitis B/di or exp HIV Infections/di or exp Chlamydia Infections/di or exp Cytomegalovirus Infections/di or exp Streptococcal Infections/di or exp Parvoviridae Infections/di or Rubella/di or Rubella Syndrome, Congenital/di or Toxoplasmosis/di or Toxoplasmosis, Congenital/di |
| 99 | exp Anemia/di or exp Blood Group Antigens/di or exp Thrombophilia/di |
| 100 | exp Urinary Tract Infections/di or Vaginosis, Bacterial/di |
| 101 | 97 or 98 or 99 or 100 |
| 102 | 75 and 101 |
| 103 | 92 or 96 or 102 |
| 104 | Neonatal Screening/ |
| 105 | (heelprick* or heel prick*).ti,ab,kw. |
| 106 | ((neonat* or newborn) adj2 screen*).ti,ab,kw. |
| 107 | exp Infant, Newborn/ |
| 108 | (newborn? or neonat* or infant?).ti,ab,kw. |
| 109 | 107 or 108 |
| 110 | Physical Examination/ |
| 111 | (physical adj3 exam*).ti,ab,kw. |
| 112 | Mass screening/ |
| 113 | screen*.ti,ab,kw. |
| 114 | Genetic testing/ |
| 115 | early diagnosis/ |
| 116 | diagnostic tests, routine/ |
| 117 | (routine adj5 (test* or diagnos*)).ti,ab,kw. |
| 118 | Serologic Tests/ |
| 119 | serologic.ti,ab,kw. |
| 120 | ((sero* adj5 diagnos*) or (serotest* or seroscreen* or serodiagnos*)).ti,ab,kw. |
| 121 | Dried Blood Spot Testing/ |
| 122 | (blood spot* or bloodspot*).ti,ab,kw. |
| 123 | exp Hearing Tests/ |
| 124 | ((hearing or auditor* or acoustic* or otoacoustic*) adj3 (test* or diagnos*)).ti,ab,kw. |
| 125 | (automated auditory brainstem response? or aabr or otoacoustic emission? or aoae).ti,ab,kw. |
| 126 | 110 or 111 or 112 or 113 or 114 or 115 or 116 or 117 or 118 or 119 or 120 or 121 or 122 or 123 or 124 or 125 |
| 127 | anemia, hemolytic, congenital/ or anemia, sickle cell/ or exp thalassemia/ |
| 128 | (sickle cell or thalass?emia?).ti,ab,kw. |
| 129 | eye diseases/ or eye diseases, hereditary/ or cataract/ or vision disorders/ or exp blindness/ |
| 130 | (cataract? or blind* or ((eye? or vision?) adj2 (disease? or disorder?))).ti,ab,kw. |
| 131 | exp Heart Defects, Congenital/ |
| 132 | ((heart or cardi* or septal or atrial or ventric*) adj2 (defect? or anomal* or malformation?)).ti,ab,kw. |
| 133 | ((coarctat* adj2 aorta) or (valv* adj2 stenosis) or "transdisposition of the great arter*" or patent ductus arteriosus or ebstein* anomal* or "tetralogy of fallot" or hypoplastic left heart syndrome or tricuspid atresia or truncus arteriosus or anomalous pulmonary venous connection).ti,ab,kw. |
| 134 | Hip Dislocation/ |
| 135 | (hip? adj2 (dysplasia? or dislocat*)).ti,ab,kw. |
| 136 | exp testicular diseases/ |
| 137 | (((undescend* or retract*) adj2 testic*) or cryptorchid*).ti,ab,kw. |
| 138 | exp Hearing Loss/ |
| 139 | ((hearing adj2 (loss or disorder?)) or deaf*).ti,ab,kw. |
| 140 | Cystic Fibrosis/ |
| 141 | cystic fibrosis.ti,ab,kw. |
| 142 | Congenital Hypothyroidism/ |
| 143 | congenital hypothyroid*.ti,ab,kw. |
| 144 | Biliary Atresia/ |
| 145 | biliary atresia.ti,ab,kw. |
| 146 | exp Genetic Diseases, Inborn/ or exp "Sex Chromosome Disorders of Sex Development"/ |
| 147 | Muscular Dystrophy, Duchenne/ or exp Muscular Atrophy, Spinal/ |
| 148 | (phenylketonuria? or medium chain acyl coa dehydrogenase deficien* or medium chain acylcoa dehydrogenase deficien* or mcadd or maple syrup urine disease? or msud or isovaleric acid?emia? or iso-valeric acid?emia? or glutaric aciduria? or homocystinuria? or amino acid metabolism disorder? or biotinidase deficiency or congenital adrenal hyperplasia or duchenne muscular dystrophy or oxidation disorder? or thrombocytop?enia? or galactos?emia? or kernicterus or dehydrogenase deficiency or lchadd or mucopolysaccharidosis or severe combined immunodeficienc* or spinal muscular atrophy or tyrosin?emia? or adrenoleukodystrophy or ccald or canavan or klinefelter syndrome or 22q11 deletion syndrome or digeorge syndrome).ti,ab. |
| 149 | 127 or 128 or 129 or 130 or 131 or 132 or 133 or 134 or 135 or 136 or 137 or 138 or 139 or 140 or 141 or 142 or 143 or 144 or 145 or 146 or 147 or 148 |
| 150 | 109 and 126 and 149 |
| 151 | 104 or 105 or 106 or 150 |
| 152 | 27 or 103 or 151 |
| 153 | Economics/ |
| 154 | exp "costs and cost analysis"/ |
| 155 | Economics, Dental/ |
| 156 | exp economics, hospital/ |
| 157 | Economics, Medical/ |
| 158 | Economics, Nursing/ |
| 159 | Economics, Pharmaceutical/ |
| 160 | (economic$ or cost or costs or costly or costing or price or prices or pricing or pharmacoeconomic$).ti,ab. |
| 161 | (expenditure$ not energy).ti,ab. |
| 162 | value for money.ti,ab. |
| 163 | budget$.ti,ab. |
| 164 | "Value of Life"/ |
| 165 | quality-adjusted life years/ |
| 166 | Decision Theory/ |
| 167 | (financ* or fiscal or funding or fee* or charge* or budget*).ti,ab. |
| 168 | (value adj2 (money or monetary)).ti,ab. |
| 169 | ("Value of life" or "quality adjusted life year*" or qaly* or qald* or qale* or "disability adjusted life year*" or daly).ti,ab. |
| 170 | (short form* or shortform*).ti,ab. |
| 171 | (sf6* or sf-6* or sf8 or sf-8 or sf10 or sf-10 or sf12 or sf-12 or sf16 or sf-16 or sf20 or sf-20 or sf36 or sf-36).ti,ab. |
| 172 | (euroqol or euro qol or "euro quality of life" or euroqual or euro qual or eq5d or eq-5d).ti,ab. |
| 173 | (AQoL* or "Assessment of Quality of Life").ti,ab. |
| 174 | ("16D Health Related Quality of Life" or 16D HRQoL or "17D Health Related Quality of Life" or 17D HRQoL).ti,ab. |
| 175 | ("Child Health Utility 9 Dimension" or CHU9D or "CHU-9D").ti,ab. |
| 176 | 15 dimensional instrument.ti,ab. |
| 177 | ("quality of wellbeing*" or "quality of well being*" or qwb).ti,ab. |
| 178 | (hye or health* year equivalent*).ti,ab. |
| 179 | (health utilit* or disutilit*).ti,ab. |
| 180 | (hui or hui1 or hui2 or hui3 or hui4 or hui-4 or hui-1 or hui-2 or hui-3).ti,ab. |
| 181 | (health* adj2 priorities).ti,ab. |
| 182 | (Adolescent Health Utility Measure or AHUM).ti,ab. |
| 183 | (preference* adj3 (valu* or measur* or health or life or estimat* or elicit* or disease or score* or instrument or instruments)).ti,ab. |
| 184 | "preference based measure of HRQoL".ti,ab. |
| 185 | (willingness adj2 pay).ti,ab. |
| 186 | standard gamble.ti,ab. |
| 187 | (time trade off or time tradeoff or tto).ti,ab. |
| 188 | (vas or visual analog*).ti,ab. |
| 189 | discrete choice*.ti,ab. |
| 190 | (utility elicitation or direct elicitation).ti,ab. |
| 191 | scoring algorithm.ti,ab. |
| 192 | best worst scaling.ti,ab. |
| 193 | (multi attribute utility or multiattribute utility).ti,ab. |
| 194 | (markov or monte carlo method).ti,ab. |
| 195 | exp Resource Allocation/ |
| 196 | Health Priorities/ |
| 197 | ((multicriteria or multi-criteria) adj2 (decision or analys* or decision aid* or decision making)).ti,ab. |
| 198 | (benefit risk asessment or risk benefit assessment).ti,ab. |
| 199 | weighted product.ti,ab. |
| 200 | ((analytic* hierarchy or analytic* network) adj process*).ti,ab. |
| 201 | ("measuring attractiveness by a categorical based evaluation technique" or "goal programming" or "elimination and choice expressing reality" or ELECTRE or "preference ranking organization method of enrichment evaluation" or PROMETHEE or "technique for order preference by similarity to ideal solution" or TOPSIS or "measuring attractiveness by a categorical based evaluation technique" or MACBETH).ti,ab. |
| 202 | "Accountability for reasonableness".ti,ab. |
| 203 | (decision* adj (tree* or model* or analysis)).ti,ab. |
| 204 | (resource* adj2 (use* or utilisation or allocat*)).ti,ab. |
| 205 | (ration or rationing).ti,ab. |
| 206 | exp Comparative Effectiveness Research/ |
| 207 | Comparative Effectiveness Research.ti,ab. |
| 208 | or/153-207 |
| 209 | 152 and 208 |
| 210 | ((energy or oxygen) adj cost).ti,ab. |
| 211 | (metabolic adj cost).ti,ab. |
| 212 | ((energy or oxygen) adj expenditure).ti,ab. |
| 213 | 210 or 211 or 212 |
| 214 | 209 not 213 |
| 215 | (comment or letter or editorial or historical article).pt. |
| 216 | 214 not 215 |
| 217 | exp animals/ not humans/ |
| 218 | 216 not 217 |
| 219 | limit 218 to yr="2000 -Current" |
| 220 | (2020* or 2021*).ed,ez,yr. |
| 221 | 219 and 220 |

### Supplementary Table 2: List of studies excluded at full-text screening stage, with brief reasons

| **Reason of exclusion** | **Source** |
| --- | --- |
| Other study design (e.g. reviews) | 1–201 |
| Non-economic evaluations | 202–256 |
| Conference abstract that has journal article published | 70,257–310 |
| Non-screening (i.e. not related to antenatal or newborn screening) | 311–333 |
| No details of economic evaluation presented in Methods/Results | 334–343 |
| Duplicate | 344–350 |
| Health preference studies | 351,352 |

### Supplementary Table 3: Characteristics of screening programmes and population in the articles and reports

|  | **Articles and reports assessing antenatal screening (%)** | **Articles and reports assessing newborn screening (%)** | **Total articles and reports (%)** |
| --- | --- | --- | --- |
| Type of screening | 242 (100) | 95 (100) | 337 (100) |
| Setting of screening* |  |  |  |
| Home | 0 (0) | 3 (3.2) | 3 (0.9) |
| Primary care | 6 (2.5) | 3 (3.2) | 9 (2.7) |
| Secondary care | 58 (24.0) | 22 (23.2) | 80 (23.7) |
| Primary and secondary care | 5 (2.1) | 4 (4.2) | 9 (2.7) |
| Not stated | 173 (71.5) | 63 (66.3) | 236 (70.0) |
| Population* |  |  |  |
| Healthy pregnancy | 196 (79.0) | 1 (1.0) | 197 (57.1) |
| Pregnant women and their partner/relative | 8 (3.2) | 0 (0) | 8 (2.3) |
| Pregnancy at risk | 37 (14.9) | 1 (1.0) | 38 (11.0) |
| Healthy infant | 7 (2.8) | 84 (86.6) | 91 (26.4) |
| Infant at risk | 0 (0) | 11 (11.3) | 11 (3.2) |
| Gestation stage of pregnant women |  |  |  |
| First trimester | 25 (9.7) | 0 (0) | 25 (7.2) |
| First or second trimester | 3 (1.2) | 0 (0) | 3 (0.9) |
| First and second trimesters | 6 (2.3) | 0 (0) | 6 (1.7) |
| First and third trimesters | 1 (0.4) | 0 (0) | 1 (0.3) |
| Second trimester | 25 (9.7) | 0 (0) | 25 (7.2) |
| Second and third trimesters | 10 (3.9) | 0 (0) | 10 (2.9) |
| Third trimester | 19 (7.4) | 0 (0) | 19 (5.5) |
| Not stated | 168 (65.4) | 2 (2.2) | 170 (49.0) |
| Not applicable | 0 (0) | 88 (97.8) | 88 (25.4) |
| Stage of disease pathway |  |  |  |
| Person at risk but no pathological changes present | 24 (9.9) | 6 (6.3) | 30 (8.9) |
| Symptomless stage with pathologically definable change present | 217 (89.7) | 86 (90.5) | 303 (89.9) |
| Signs and/or symptoms exist but condition undiagnosed | 1 (0.4) | 2 (2.1) | 3 (0.9) |
| Clinical phase | 0 (0) | 1 (1.1) | 1 (0.3) |
| Phase(s) of screening programme |  |  |  |
| Screening and diagnostic | 60 (24.8) | 16 (16.8) | 76 (22.6) |
| Screening and intervention | 59 (24.4) | 10 (10.5) | 69 (20.5) |
| Screening, diagnostic and intervention | 103 (42.6) | 59 (62.1) | 162 (48.1) |
| Not clear | 20 (8.3) | 10 (10.5) | 30 (8.9) |

* Will not total up to 100% as some articles and reports presented more than one category.

### Supplementary Table 4: Medical conditions investigated

| **Conditions** | **Articles and reports assessing antenatal screening (%)** | **Articles and reports assessing newborn screening (%)** |
| --- | --- | --- |
| Developmental | 0 (0) | 6 (6.3) |
| Endocrinology | 24 (9.9) | 4 (4.2) |
| Genetic | 77 (31.8) | 12 (12.6) |
| Gestational cardiorenal | 5 (2.1) | 0 (0) |
| Haematology | 18 (7.4) | 12 (12.6) |
| Infection | 76 (31.4) | 3 (3.2) |
| Intrauterine fetal demise/ sudden infant death syndrome | 1 (0.4) | 1 (1.1) |
| Maternal mental health | 1 (0.4) | 6 (6.3) |
| Metabolic | 0 (0) | 32 (33.7) |
| Neurodevelopment | 1 (0.4) | 3 (3.2) |
| Neurological | 0 (0) | 1 (1.1) |
| Nutrition | 4 (1.7) | 0 (0) |
| Structural | 36 (14.9) | 25 (26.3) |
| Urology | 1 (0.4) | 0 (0) |
| All conditions* | 242 | 95 |

*The sum of the individual conditions is not equivalent to the total value across all conditions because there are five articles and reports that investigated more than one condition.

### Supplementary Table 5: Summary of reporting quality of articles and reports (excluding conference abstracts) assessed using CHEERS checklist

| **CHEERS item no.** | **CHEERS item** | **Articles and reports assessing antenatal screening (%)** | | | **Articles and reports assessing newborn screening (%)** | | |
| --- | --- | --- | --- | --- | --- | --- | --- |
|  |  | **Satisfied** | **Not satisfied** | **Not applicable** | **Satisfied** | **Not satisfied** | **Not applicable** |
|  | ***Title and abstract*** |  |  |  |  |  |  |
| 1 | Title | 155 (85.6) | 26 (14.4) | 0 (0) | 70 (86.4) | 11 (13.6) | 0 (0) |
| 2 | Abstract | 21 (11.6) | 160 (88.4) | 0 (0) | 13 (15.7) | 69 (83.1) | 1 (1.2) |
|  | ***Introduction*** |  |  |  |  |  |  |
| 3 | Background and objectives | 181 (100) | 0 (0) | 0 (0) | 83 (100) | 0 (0) | 0 (0) |
|  | ***Methods*** |  |  |  |  |  |  |
| 4 | Target population and subgroups | 181 (100) | 0 (0) | 0 (0) | 83 (100) | 0 (0) | 0 (0) |
| 5 | Setting and location | 65 (35.9) | 116 (64.1) | 0 (0) | 30 (36.1) | 53 (63.9) | 0 (0) |
| 6 | Study perspective | 134 (74) | 47 (26) | 0 (0) | 67 (80.7) | 15 (18.1) | 1 (1.2) |
| 7 | Comparators | 159 (87.8) | 22 (12.2) | 0 (0) | 75 (90.4) | 8 (9.6) | 0 (0) |
| 8 | Time horizon | 28 (15.5) | 153 (84.5) | 0 (0) | 15 (18.1) | 67 (80.7) | 1 (1.2) |
| 9 | Discount rate | 51 (28.2) | 130 (71.8) | 0 (0) | 29 (34.9) | 53 (63.9) | 1 (1.2) |
| 10 | Choice of health outcomes | 181 (100) | 0 (0) | 0 (0) | 82 (98.8) | 0 (0) | 1 (1.2) |
| 11 | Measurement of effectiveness | 178 (98.3) | 3 (1.7) | 0 (0) | 82 (98.8) | 1 (1.2) | 0 (0) |
| 12 | Measurement and valuation of preference-based outcomes | 48 (26.5) | 34 (18.8) | 99 (54.7) | 19 (22.9) | 13 (15.7) | 51 (61.4) |
| 13 | Estimate resources and cost | 168 (92.8) | 13 (7.2) | 0 (0) | 79 (95.2) | 3 (3.6) | 1 (1.2) |
| 14 | Currency, price date, and conversion | 139 (76.8) | 42 (23.2) | 0 (0) | 64 (77.1) | 18 (21.7) | 1 (1.2) |
| 15 | Choice of model | 28 (15.5) | 153 (84.5) | 0 (0) | 28 (33.7) | 55 (66.3) | 0 (0) |
| 16 | Assumptions | 131 (72.4) | 20 (11) | 30 (16.6) | 60 (72.3) | 6 (7.2) | 17 (20.5) |
| 17 | Analytic method | 116 (64.1) | 65 (35.9) | 0 (0) | 63 (75.9) | 20 (24.1) | 0 (0) |
|  | ***Results*** |  |  |  |  |  |  |
| 18 | Study parameters | 155 (85.6) | 26 (14.4) | 0 (0) | 73 (88) | 9 (10.8) | 1 (1.2) |
| 19 | Incremental costs and outcomes | 168 (92.8) | 13 (7.2) | 0 (0) | 75 (90.4) | 7 (8.4) | 1 (1.2) |
| 20 | Characterizing uncertainty | 161 (89) | 20 (11) | 0 (0) | 69 (83.1) | 13 (15.7) | 1 (1.2) |
| 21 | Characterizing heterogeneity | 24 (13.3) | 3 (1.7) | 154 (85.1) | 4 (4.8) | 0 (0) | 79 (95.2) |
|  | ***Discussion*** |  |  |  |  |  |  |
| 22 | Study funding, limitation, generalisability, and current knowledge | 58 (32) | 123 (68) | 0 (0) | 23 (27.7) | 59 (71.1) | 1 (1.2) |
|  | ***Other*** |  |  |  |  |  |  |
| 23 | Source of funding | 100 (55.2) | 81 (44.8) | 0 (0) | 55 (66.3) | 28 (33.7) | 0 (0) |
| 24 | Conflict of interest | 107 (59.1) | 74 (40.9) | 0 (0) | 51 (61.4) | 32 (38.6) | 0 (0) |

### Supplementary Table 6: Thematic framework of benefits and harms adopted by health economic assessments evaluating antenatal and newborn screening programmes

| **Theme no.** | **Theme** | **Subtheme level 1** | **Subtheme level 2** | **Subtheme level 3** | **Subtheme level 4** | **Antenatal screening** | **Newborn screening** |
| --- | --- | --- | --- | --- | --- | --- | --- |
| 1 | Diagnosis of screened for condition | additional screening of partners |  |  |  | 353 | - |
| 1 | Diagnosis of screened for condition | additional testing to reach diagnosis in the absence of screening (links to diagnostic odyssey) |  |  |  | 354–356 | 357 |
| 1 | Diagnosis of screened for condition | born with condition | reduction in children born with condition through effective treatment of the screened for condition |  |  | 358–380 | 361,381 |
| 1 | Diagnosis of screened for condition | born with condition | reduction in children born with condition through termination of pregnancy |  |  | 382–408 | - |
| 1 | Diagnosis of screened for condition | cases diagnosed at screening |  |  |  | 356,382–384,386,388,391,397,398,408–430 | 357,431–445 |
| 1 | Diagnosis of screened for condition | cases diagnosed at screening rather than later symptomatically |  |  |  | 390,404,406,446–448 | 276,449–455 |
| 1 | Diagnosis of screened for condition | cases diagnosed at screening that would have become symptomatic |  |  |  | - | 456 |
| 1 | Diagnosis of screened for condition | cases diagnosed at screening | maternal |  |  | 385 | - |
| 1 | Diagnosis of screened for condition | cases missed at screening |  |  |  | 367,402,411,416,417,428,429,457,458 | 459,460 |
| 1 | Diagnosis of screened for condition | cases missed at screening | legal cost of reimbursing false negatives |  |  | - | 461 |
| 1 | Diagnosis of screened for condition | confirmatory test and additional tests to reach diagnosis of screened for condition |  |  |  | 270,382–384,404,409,462–465 | 357,433,466–470 |
| 1 | Diagnosis of screened for condition | confirmatory test and additional tests to reach diagnosis of screened for condition [invasive] |  |  |  | 355,402 | - |
| 1 | Diagnosis of screened for condition | confirmatory test and additional tests to reach diagnosis of screened for condition | early vs late |  |  | - | 435 |
| 1 | Diagnosis of screened for condition | confirmatory test and additional tests to reach diagnosis of screened for condition | unnecessary due to false positive |  |  | 353,356,358,360,362–365,369–371,374–376,378–380,385,388,390,391,393,397,398,402,403,406,410,411,414–416,418,422,423,425,446–448,471–494 | 276,278,283,381,431,432,434,436–445,449,451–454,456,459,461,469,470,495–522 |
| 1 | Diagnosis of screened for condition | confirmatory test and additional tests to reach diagnosis of screened for condition | unnecessary due to false positive [invasive] |  |  | 355,392,399,400,405,412,413,415,417,419,421,424,426,428,429,457,523–526 | 527 |
| 1 | Diagnosis of screened for condition | confirmatory test and additional tests to reach diagnosis of screened for condition | unnecessary due to false positive | time spent attending |  | - | 431 |
| 1 | Diagnosis of screened for condition | screened for condition related complications | morbidity |  |  | - | 438 |
| 2 | Life years and health status adjustments | infant life years post birth | DALYs |  |  | 378,482 | 469,506 |
| 2 | Life years and health status adjustments | infant life years post birth | morbidity |  |  | 374,474,528,529 | 444 |
| 2 | Life years and health status adjustments | infant life years post birth | mortality |  |  | 362,373,374,380,387,430,479,494,528,530,531 | 437,444,519 |
| 2 | Life years and health status adjustments | infant life years post birth | QALYs |  |  | 362,374,376,380,387,464,472,473,479,480,483,488,532–548 | 361,443,444,459,469,496,497,500–503,506,510,511,515,521,549–552 |
| 2 | Life years and health status adjustments | infant life years post birth | replacement of affected aborted fetus with subsequent unaffected pregnancy and life |  |  | 407 | - |
| 2 | Life years and health status adjustments | infant life years post birth | years |  |  | 360,364,366,375,385,403,474,486,487,489,530,535,553,554 | 283,357,361,381,435,444,449,461,468,470,497,498,500,504,505,509,512–517,520,522,552,555,556 |
| 2 | Life years and health status adjustments | infant life years post birth | years | monetary value |  | - | 557 |
| 2 | Life years and health status adjustments | maternal life years | mortality |  |  | 369,374 | 558 |
| 2 | Life years and health status adjustments | maternal life years | QALYs |  |  | 270,376,387,394–396,412,418,420,422,430,458,463–465,471,472,474,476,479–481,483–485,487,490,492–494,523,525,529,531,533,534,537,539,541,545–547,559–571 | 278,466,469,508,515,518,519,527,557 |
| 2 | Life years and health status adjustments | maternal life years | QALYs | decrement due to caring for child with screened for condition |  | 572 | - |
| 2 | Life years and health status adjustments | maternal life years | years |  |  | 360,385,403,474,486,487,489,553,554 | 283,361,381,444,468,470,498,500,504,509,512–515,520,522,552 |
| 2 | Life years and health status adjustments | parental QALYs |  |  |  | - | 449,461,495,507 |
| 2 | Life years and health status adjustments | psychological | anxiety from genetic variants of unclear penetrance |  |  | - | 466 |
| 2 | Life years and health status adjustments | psychological | disutility due to knowledge of disease |  |  | 484 | - |
| 2 | Life years and health status adjustments | psychological | disutility due to knowledge of disease in those with positive screening results (stress and anxiety) |  |  | 422 | - |
| 2 | Life years and health status adjustments | psychological | early diagnosis induced anxiety |  |  | - | 466 |
| 2 | Life years and health status adjustments | psychological | false positive anxiety |  |  | 424 | 439,497 |
| 2 | Life years and health status adjustments | psychological | false positive anxiety | parental QALYs |  | - | 461 |
| 2 | Life years and health status adjustments | psychological | genetic variants of unclear penetrance | unclear harms including behavioural changes | QALYs | - | 466 |
| 2 | Life years and health status adjustments | screened for condition associated mortality / treatment associated mortality/other causes mortality |  |  |  | - | 556 |
| 3 | Treatment | additional healthcare post-diagnosis |  |  |  | 359,528 | 455,466,496 |
| 3 | Treatment | comparison of earlier treatment after screen detection and later after symptomatic detection | increase in treatment required for false negative |  |  | 533,572 | 440,468 |
| 3 | Treatment | comparison of earlier treatment after screen detection and later after symptomatic detection | increase in treatment required for false negative | prior to diagnosis |  | - | 510 |
| 3 | Treatment | comparison of earlier treatment after screen detection and later after symptomatic detection | reduction in treatment required |  |  | 354,548,573 | - |
| 3 | Treatment | comparison of earlier treatment after screen detection and later after symptomatic detection | reduction in treatment required | costs |  | 369,370,422,476,488,535,548,560,564,574–576 | 435,444,461,468,498,499,504,508,515,516,520,522 |
| 3 | Treatment | comparison of earlier treatment after screen detection | adverse complications of screened for condition averted |  |  | 353,354,368–370,410,414,465,477,479,482,487,491,533,545–547,554,560,566,568,576–578 | 6,361,456,513,519,579 |
| 3 | Treatment | hospital stay |  |  |  | 478,540,580 | 503,517 |
| 3 | Treatment | missed due to false negative |  |  |  | 371 | - |
| 3 | Treatment | prevention of screened for condition (infectious) | increase in future earning potential |  |  | 580 | - |
| 3 | Treatment | prevention of screened for condition (infectious) | unnecessary prophylaxis in false positives |  |  | 368,371,372,374,376,379,473,488,532,533,543,570,573,576,580 | - |
| 3 | Treatment | prevention of screened for condition (infectious) | unnecessary prophylaxis in false positives | allergic reaction |  | 570 | - |
| 3 | Treatment | psychological | counselling about genetic diagnosis |  |  | 382,397 | - |
| 3 | Treatment | psychological | counselling about screening or confirmatory test | false positive |  | 366,376,405,406,424,543 | - |
| 3 | Treatment | psychological | counselling about screening or confirmatory test |  |  | 353,382,397 | - |
| 3 | Treatment | screened for condition related treatment/management |  |  |  | 270,353,378,382,409,422,458,464,465,475,546,581 | 467–470,556 |
| 3 | Treatment | screened for condition related treatment/management | unnecessary in false positives with no confirmatory test |  |  | 359,360,362–364,367,371,377,380,385,403,422,471,476,479–481,484,487,490,528,529,538–540,542,545,547,548,553,554,561,562,564,567,572,578 | 276,455,461,498,501,502,505,513,517,521,550,551,557,582 |
| 3 | Treatment | treatment related harm | adverse reaction to treatment |  |  | 372,374,480,485,489,491,532,560,570,572 | 283,505,509,513,517,520,583 |
| 3 | Treatment | treatment related harm | antibiotic resistance |  |  | 359 | - |
| 3 | Treatment | treatment related harm | disutility of treatment |  |  | 487 | - |
| 3 | Treatment | treatment related harm | treatment related anxiety |  |  | - | 515 |
| 3 | Treatment | unnecessary due to false positive |  |  |  | 375,474,544,546 | - |
| 4 | Long-term cost associated with screened for condition | cost-savings from averted births of fetuses with anomalies |  |  |  | 354 | - |
| 4 | Long-term cost associated with screened for condition | direct healthcare and non-healthcare cost |  |  |  | 353,374,480 | 497 |
| 4 | Long-term cost associated with screened for condition | direct healthcare and non-healthcare cost | healthcare and productivity gains |  |  | 407 | - |
| 4 | Long-term cost associated with screened for condition | direct healthcare and non-healthcare cost | healthcare and social services |  |  | - | 518 |
| 4 | Long-term cost associated with screened for condition | direct healthcare and non-healthcare cost | healthcare, education and social services |  |  | - | 466,502 |
| 4 | Long-term cost associated with screened for condition | direct healthcare and non-healthcare cost | social services |  |  | 380 | 515 |
| 4 | Long-term cost associated with screened for condition | direct healthcare and non-healthcare cost | treatment and caregiving |  |  | 403 | 519 |
| 4 | Long-term cost associated with screened for condition | direct healthcare cost |  |  |  | 270,360,363,364,366,369,373,375,376,382,393,399,401,405,407,457,462,464,465,474,477,479,482–484,486–488,493,530–532,534–536,538,543,544,548,553,559,562,567,568,571,572,575,576,580,581 | 381,443,444,449,450,452,459,461,469,470,496,500,501,503,504,510,511,513,514,522,552,556,584,585 |
| 4 | Long-term cost associated with screened for condition | direct non-healthcare cost |  |  |  | 385 | - |
| 4 | Long-term cost associated with screened for condition | direct non-healthcare cost | caregiving |  |  | 384,473,524,586 | 582 |
| 4 | Long-term cost associated with screened for condition | direct non-healthcare cost | child protective services investigation and foster care placements if mothers successfully completed substance abuse treatment |  |  | 530 | - |
| 4 | Long-term cost associated with screened for condition | direct non-healthcare cost | education and social services |  |  | - | 435 |
| 4 | Long-term cost associated with screened for condition | direct non-healthcare cost | social care |  |  | - | 504 |
| 4 | Long-term cost associated with screened for condition | productivity gains |  |  |  | 370,562 | 550 |
| 4 | Long-term cost associated with screened for condition | societal cost |  |  |  | 356,412,425,523,525 | 276,278,495,550 |
| 5 | Overdiagnosis | QALY decrement |  |  |  | 480 | 515 |
| 5 | Overdiagnosis | unnecessary test/treatment |  |  |  | 477,480 | 461,502,515 |
| 6 | Pregnancy loss | spontaneous |  |  |  | 356,359,383,384,387,402,406,427,478,492,572 | - |
| 6 | Pregnancy loss | termination |  |  |  | 355,387,419,430,571 | - |
| 6 | Pregnancy loss | termination | date/trimester |  |  | 397,412,523 | - |
| 6 | Pregnancy loss | termination | of unaffected fetus due to false positive test result |  |  | 381,386–388,390,391,400,405,415,416,418,420,424,427,579 | - |
| 6 | Pregnancy loss | termination | of unaffected fetus due to false positive test result | psychological consequences |  | 419 | - |
| 6 | Pregnancy loss | termination | prevent downstream adverse maternal outcomes |  |  | 526 | - |
| 6 | Pregnancy loss | termination | prevent later miscarriage |  |  | 359,397 | - |
| 6 | Pregnancy loss | termination | psychological consequences |  |  | 419,526,572 | - |
| 6 | Pregnancy loss | treatment/test related |  |  |  | 278,355,356,382–384,388–392,394,395,397,398,400,402,404–406,408,409,411–413,415,416,418–421,424–429,447,448,457,472,492,525,526,548,571,572,579,584 | - |
| 6 | Pregnancy loss | treatment/test related | unaffected |  |  | 355,382 | - |
| 7 | Spillover effects | benefits to parents from child’s diagnosis with genetic condition, through knowledge of their own genetic status |  |  |  | - | 557 |

### Supplementary Table 7: Summary of thematic framework of benefits and harms adopted by health economic assessments evaluating antenatal and newborn screening programmes by conditions

| **Screening type** | **Core theme** | **Developmental** | **Endocrinology** | **Genetic** | **Gestational cardiorenal** | **Haematology** | **Infection** | **Intrauterine fetal demise** | **Maternal mental health** | **Metabolic** | **Neurodevelopment** | **Neurological** | **Nutrition** | **Structural** | **Urology** |
| --- | --- | --- | --- | --- | --- | --- | --- | --- | --- | --- | --- | --- | --- | --- | --- |
| Antenatal | Total no. of studies | 0 (0%) | 24 (100%) | 77 (100%) | 5 (100%) | 18 (100%) | 76 (100%) | 1 (100%) | 1 (100%) | 0 (0%) | 1 (100%) | 0 (0%) | 4 (100%) | 36 (100%) | 1 (100%) |
|  | No themes* | 0 (0%) | 9 (38%) | 24 (31%) | 1 (20%) | 8 (44%) | 22 (29%) | 0 (0%) | 0 (0%) | 0 (0%) | 0 (0%) | 0 (0%) | 1 (25%) | 16 (44%) | 0 (0%) |
|  | Theme 1 | 0 (0%) | 8 (33%) | 49 (64%) | 3 (60%) | 7 (39%) | 32 (42%) | 0 (0%) | 1 (100%) | 0 (0%) | 0 (0%) | 0 (0%) | 2 (50%) | 14 (39%) | 1 (100%) |
|  | Theme 2 | 0 (0%) | 13 (54%) | 14 (18%) | 2 (40%) | 4 (22%) | 38 (50%) | 1 (100%) | 1 (100%) | 0 (0%) | 1 (100%) | 0 (0%) | 2 (50%) | 14 (39%) | 0 (0%) |
|  | Theme 3 | 0 (0%) | 11 (46%) | 8 (10%) | 4 (80%) | 7 (39%) | 47 (62%) | 1 (100%) | 1 (100%) | 0 (0%) | 0 (0%) | 0 (0%) | 2 (50%) | 7 (19%) | 1 (100%) |
|  | Theme 4 | 0 (0%) | 4 (17%) | 15 (19%) | 1 (20%) | 3 (17%) | 36 (47%) | 0 (0%) | 0 (0%) | 0 (0%) | 1 (100%) | 0 (0%) | 0 (0%) | 8 (22%) | 1 (100%) |
|  | Theme 5 | 0 (0%) | 0 (0%) | 0 (0%) | 0 (0%) | 0 (0%) | 2 (3%) | 0 (0%) | 0 (0%) | 0 (0%) | 0 (0%) | 0 (0%) | 0 (0%) | 0 (0%) | 0 (0%) |
|  | Theme 6 | 0 (0%) | 0 (0%) | 43 (56%) | 0 (0%) | 2 (11%) | 2 (3%) | 0 (0%) | 0 (0%) | 0 (0%) | 0 (0%) | 0 (0%) | 1 (25%) | 6 (17%) | 0 (0%) |
|  | Theme 7 | 0 (0%) | 0 (0%) | 0 (0%) | 0 (0%) | 0 (0%) | 0 (0%) | 0 (0%) | 0 (0%) | 0 (0%) | 0 (0%) | 0 (0%) | 0 (0%) | 0 (0%) | 0 (0%) |
| Newborn | Total no. of studies | 6 (100%) | 4 (100%) | 12 (100%) | 0 (0%) | 12 (100%) | 3 (100%) | 1 (100%) | 6 (0%) | 32 (100%) | 3 (100%) | 1 (100%) | 0 (0%) | 25 (100%) | 0 (0%) |
|  | No themes* | 3 (50%) | 1 (25%) | 1 (8%) | 0 (0%) | 3 (25%) | 0 (0%) | 0 (0%) | 3 (0%) | 9 (28%) | 1 (33%) | 0 (0%) | 0 (0%) | 3 (12%) | 0 (0%) |
|  | Theme 1 | 2 (33%) | 2 (50%) | 8 (67%) | 0 (0%) | 8 (67%) | 1 (33%) | 1 (100%) | 2 (0%) | 22 (69%) | 1 (33%) | 0 (0%) | 0 (0%) | 19 (76%) | 0 (0%) |
|  | Theme 2 | 3 (50%) | 1 (25%) | 8 (67%) | 0 (0%) | 9 (75%) | 0 (0%) | 1 (100%) | 3 (0%) | 19 (59%) | 2 (67%) | 1 (100%) | 0 (0%) | 8 (32%) | 0 (0%) |
|  | Theme 3 | 1 (17%) | 1 (25%) | 3 (25%) | 0 (0%) | 7 (58%) | 1 (33%) | 1 (100%) | 1 (0%) | 14 (44%) | 1 (33%) | 1 (100%) | 0 (0%) | 11 (44%) | 0 (0%) |
|  | Theme 4 | 1 (17%) | 2 (50%) | 5 (42%) | 0 (0%) | 4 (33%) | 1 (33%) | 1 (100%) | 1 (0%) | 17 (53%) | 1 (33%) | 0 (0%) | 0 (0%) | 7 (28%) | 0 (0%) |
|  | Theme 5 | 0 (0%) | 0 (0%) | 0 (0%) | 0 (0%) | 0 (0%) | 0 (0%) | 0 (0%) | 0 (0%) | 3 (9%) | 0 (0%) | 0 (0%) | 0 (0%) | 0 (0%) | 0 (0%) |
|  | Theme 7 | 0 (0%) | 0 (0%) | 1 (8%) | 0 (0%) | 0 (0%) | 2 (67%) | 0 (0%) | 0 (0%) | 0 (0%) | 0 (0%) | 0 (0%) | 0 (0%) | 0 (0%) | 0 (0%) |

Theme 1: Diagnosis of screened for condition, Theme 2: Life years and health status adjustments, Theme 3: Treatment, Theme 4: Long-term cost associated with screened for condition, Theme 5: Overdiagnosis, Theme 6: Pregnancy loss, Theme 7: Spillover effects.
*Limited information about benefits and harms could be extracted to inform our bespoke form as the majority in this category were conference abstracts.

### Supplementary Table 8: Thematic framework of benefits and harms adopted by health economic assessments evaluating antenatal and newborn screening programmes in the refresh search from 1^st^ January 2021 to 22^nd^ November 2021

| **Theme no.** | **Theme** | **Subtheme level 1** | **Subtheme level 2** | **Subtheme level 3** | **Antenatal screening** | **Newborn screening** |
| --- | --- | --- | --- | --- | --- | --- |
| 1 | Diagnosis of screened for condition | born with condition | reduction in children born with condition through termination of pregnancy |  | 587 |  |
| 1 | Diagnosis of screened for condition | cases diagnosed at screening |  |  | 588–591 |  |
| 1 | Diagnosis of screened for condition | cases diagnosed at screening that would have become symptomatic |  |  |  | 592 |
| 1 | Diagnosis of screened for condition | cases missed at screening |  |  | 588 |  |
| 1 | Diagnosis of screened for condition | confirmatory test and additional tests to reach diagnosis of screened for condition | unnecessary due to false positive |  | 591,593,594 | 592 |
| 1 | Diagnosis of screened for condition | confirmatory test and additional tests to reach diagnosis of screened for condition | unnecessary due to false positive [invasive] |  | 588,595 |  |
| 1 | Diagnosis of screened for condition | screened for condition related complications | morbidity |  | 596 |  |
| 2 | Life years and health status adjustments | infant life years post birth | DALYs |  | 596 |  |
| 2 | Life years and health status adjustments | infant life years post birth | morbidity |  | 590,595–597 |  |
| 2 | Life years and health status adjustments | infant life years post birth | mortality |  | 589,590,597,598 |  |
| 2 | Life years and health status adjustments | infant life years post birth | QALYs |  | 593,598 | 592,599 |
| 2 | Life years and health status adjustments | maternal life years | mortality |  | 594 |  |
| 2 | Life years and health status adjustments | maternal life years | QALYs |  | 587,591,594,598 |  |
| 3 | Treatment | comparison of earlier treatment after screen detection and later after symptomatic detection | reduction in treatment required | costs | 598 |  |
| 3 | Treatment | comparison of earlier treatment after screen detection | adverse complications of screened for condition averted |  | 594,597,598 |  |
| 3 | Treatment | hospital stay |  |  | 590 |  |
| 3 | Treatment | treatment related harm | adverse reaction to treatment |  | 597 |  |
| 3 | Treatment | unnecessary due to false positive |  |  | 589,590,594–598 |  |
| 6 | Pregnancy loss | termination of pregnancy |  |  | 588,595 |  |
| 6 | Pregnancy loss | treatment/test related |  |  | 587,595 |  |
| 6 | Pregnancy loss | treatment/test related | unaffected |  | 588 |  |
| 7 | Spillover effects |  |  |  | 593 |  |

*Note: Five studies from refresh search were not presented here as they did not include benefits/harms of antenatal/newborn screening*^600–604^*.*

## Supplementary Figures

### Supplementary Figure 1: Number of articles and reports published from 2000 to January 2021


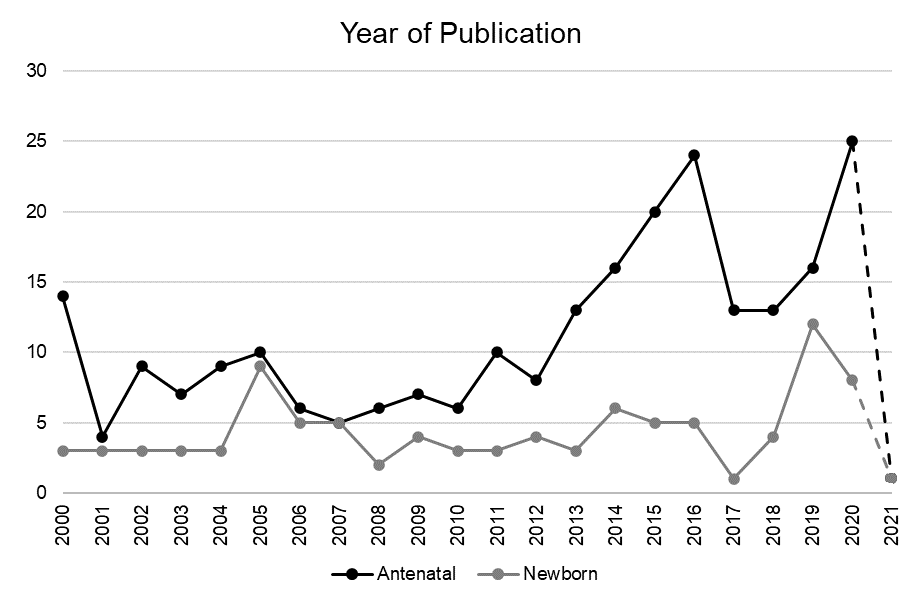


Note: Dotted lines were used to indicate that only January 2021 was included in this chart.

## References of Supplementary Material

1 Anonymous. Cost-effectiveness of voluntary prenatal and routine newborn HIV screening. *AIDS Reader* 2001;**11**:81.

2 Anonymous. Non-invasive Prenatal Testing: A Review of the Cost Effectiveness and Guidelines. *Canadian Agency for Drugs and Technologies in Health CADTH Rapid Response Reports* 2014;**02**:10.

3 Asciutto R, Di Napoli A, Vecchi S, Sicuro J, Mirisola C, Petrelli A. A systematic review of economic evaluations of neonatal and maternal healthcare in immigrant and ethnic minority women. *Epidemiologia e Prevenzione* 2020;**44**:142–52. https://doi.org/10.19191/EP20.5-6.S1.P142.084.

4 Bert F, Gualano MR, Biancone P, Brescia V, Camussi E, Martorana M, *et al.* HIV screening in pregnant women: A systematic review of cost-effectiveness studies. *International Journal of Health Planning and Management* 2018;**33**:31–50.

5 Bobic B, Villena I, Stillwaggon E. Prevention and mitigation of congenital toxoplasmosis. Economic costs and benefits in diverse settings. *Food and Waterborne Parasitology* 2019;**16**:e00058.

6 Bradshaw EA, Martin GR. Screening for critical congenital heart disease: advancing detection in the newborn. *Current Opinion in Pediatrics* 2012;**24**:603–8.

7 Brice P, Jarrett J, Mugford M. Genetic screening for cystic fibrosis: An overview of the science and the economics. *Journal of Cystic Fibrosis* 2007;**6**:255–61.

8 Bryan S, Dormandy E, Roberts T, Ades A, Barton P, Juarez-Garcia A, *et al.* Screening for sickle cell and thalassaemia in primary care: a cost-effectiveness study. *British Journal of General Practice* 2011;**61**:e620-7.

9 Cacciatore P, Visser LA, Buyukkaramikli N, van der Ploeg CPB, van den Akker-van Marle ME. The Methodological Quality and Challenges in Conducting Economic Evaluations of Newborn Screening: A Scoping Review. *International Journal of Neonatal Screening* 2020;**6**:. https://doi.org/10.3390/ijns6040094.

10 Castilla-Rodriguez I, Vallejo-Torres L, Couce ML, Valcarcel-Nazco C, Mar J, Serrano-Aguilar P. Cost-Effectiveness Methods and Newborn Screening Assessment. *Advances in Experimental Medicine and Biology* 2017;**1031**:267–81.

11 Caughey AB, Kaimal AJ, Odibo AO. Cost-effectiveness of Down syndrome screening paradigms. *Clinics in Laboratory Medicine* 2010;**30**:629–42.

12 Colbourn TE, Asseburg C, Bojke L, Philips Z, Welton NJ, Claxton K, *et al.* Preventive strategies for group B streptococcal and other bacterial infections in early infancy: cost effectiveness and value of information analyses. *BMJ* 2007;**335**:655.

13 Colgan S, Gold L, Wirth K, Ching T, Poulakis Z, Rickards F, *et al.* The cost-effectiveness of universal newborn screening for bilateral permanent congenital hearing impairment: systematic review. *Academic Pediatrics* 2012;**12**:171–80.

14 Coward S, Leggett L, Kaplan GG, Clement F. Cost-effectiveness of screening for hepatitis C virus: a systematic review of economic evaluations. *BMJ Open* 2016;**6**:e011821.

15 DeVore GR. Is genetic ultrasound cost-effective? *Seminars in Perinatology* 2003;**27**:173–82.

16 Ewer AK, Furmston AT, Middleton LJ, Deeks JJ, Daniels JP, Pattison HM, *et al.* Pulse oximetry as a screening test for congenital heart defects in newborn infants: a test accuracy study with evaluation of acceptability and cost-effectiveness. *Health Technology Assessment* 2012;**16**:v–xiii, 1.

17 Ferrier C, Dhombres F, Guilbaud L, Durand-Zaleski I, Jouannic JM. Ultrasound screening for birth defects: A medico-economic review. *Gynecologie, Obstetrique, Fertilite and Senologie* 2017;**45**:408–15.

18 Fitria N, van Asselt ADI, Postma MJ. Cost-effectiveness of controlling gestational diabetes mellitus: a systematic review. *European Journal of Health Economics* 2019;**20**:407–17.

19 Garcia-Perez L, Linertova R, Alvarez-de-la-Rosa M, Bayon JC, Imaz-Iglesia I, Ferrer-Rodriguez J, *et al.* Cost-effectiveness of cell-free DNA in maternal blood testing for prenatal detection of trisomy 21, 18 and 13: a systematic review. *European Journal of Health Economics* 2018;**19**:979–91.

20 Geue C, Wu O, Xin Y, Heggie R, Hutchinson S, Martin NK, *et al.* Cost-effectiveness of HBV and HCV screening strategies - A systematic review of existing modelling techniques. *PLoS ONE* 2015;**10**:.

21 Gilbert RE, Peckham CS. Congenital toxoplasmosis in the United Kingdom: To screen or not to screen? *Journal of Medical Screening* 2002;**9**:135–41.

22 Gliddon HD, Peeling RW, Kamb ML, Toskin I, Wi TE, Taylor MM. A systematic review and meta-analysis of studies evaluating the performance and operational characteristics of dual point-of-care tests for HIV and syphilis. *Sexually Transmitted Infections* 2017;**93**:S3–15.

23 Gonzalez FM, Veneziano MA, Puggina A, Boccia S. A Systematic Review on the Cost-Effectiveness of Genetic and Electrocardiogram Testing for Long QT Syndrome in Infants and Young Adults. *Value in Health* 2015;**18**:700–8.

24 Grosse SD. Showing Value in Newborn Screening: Challenges in Quantifying the Effectiveness and Cost-Effectiveness of Early Detection of Phenylketonuria and Cystic Fibrosis. *Healthcare* 2015;**3**:1133–57.

25 Grosse SD, Mason CA, Gaffney M, Thomson V, White KR. What Contribution Did Economic Evidence Make to the Adoption of Universal Newborn Hearing Screening Policies in the United States? *International Journal of Neonatal Screening* 2018;**4**:25.

26 Grosse SD, Olney RS, Baily MA. The cost effectiveness of universal versus selective newborn screening for sickle cell disease in the US and the UK: a critique. *Applied Health Economics & Health Policy* 2005;**4**:239–47.

27 Grosse SD, Rogowski WH, Ross LF, Cornel MC, Dondorp WJ, Khoury MJ. Population screening for genetic disorders in the 21st century: evidence, economics, and ethics. *Public Health Genomics* 2010;**13**:106–15.

28 Grosse SD, Teutsch SM, Haddix AC. Lessons from cost-effectiveness research for United States public health policy. *Annual Review of Public Health* 2007;**28**:365–91.

29 Grosse SD, Thompson JD, Ding Y, Glass M. The Use of Economic Evaluation to Inform Newborn Screening Policy Decisions: The Washington State Experience. *Milbank Quarterly* 2016;**94**:366–91. https://doi.org/10.1016/j.jpeds.2016.01.029. [38] Peterson C, Grosse SD, Oster ME, Olney RS, Cassell CH. Cost‐effectiveness of routine screening for critical congenital heart disease in US newborns. Pediatrics. 2013; 132( 3): e595‐ 603. [39] Grosse SD. Economic analyses of genetic tests in personalized medicine: clinical utility first, then cost utility. Genet Med. 2014; 16( 3): 225‐ 227. [42] Grosse SD. Economic evaluations of newborn screening interventions. In: WJ Ungar, ed. Economic Evaluation in Child Heal.

30 Grosse SD, Van Vliet G. How many deaths can be prevented by newborn screening for congenital adrenal hyperplasia? *Hormone Research* 2007;**67**:284–91.

31 Grosse SD, Van Vliet G. Challenges in Assessing the Cost-Effectiveness of Newborn Screening: The Example of Congenital Adrenal Hyperplasia. *International Journal of Neonatal Screening* 2020;**6**:. https://doi.org/10.3390/ijns6040082.

32 Hahne SJ, Veldhuijzen IK, Wiessing L, Lim TA, Salminen M, Laar M. Infection with hepatitis B and C virus in Europe: a systematic review of prevalence and cost-effectiveness of screening. *BMC Infectious Diseases* 2013;**13**:181.

33 Hieronimus S, Le Meaux JP. Relevance of gestational diabetes mellitus screening and comparison of selective with universal strategies. *Diabetes and Metabolism* 2010;**36**:575–86.

34 Honest H, Forbes C, Durée K, Norman G, Duffy S, Tsourapas A, *et al.* Screening to prevent spontaneous preterm birth: systematic reviews of accuracy and effectiveness literature with economic modelling. *Health Technology Assessment* 2009;**13**:1–627.

35 Hubbard HB. A primer on economic evaluations related to expansion of newborn screening for genetic and metabolic disorders. *JOGNN: Journal of Obstetric, Gynecologic, & Neonatal Nursing* 2006;**35**:692–9.

36 Huntington S, Weston G, Seedat F, Marshall J, Bailey H, Tebruegge M, *et al.* Repeat screening for syphilis in pregnancy as an alternative screening strategy in the UK: a cost-effectiveness analysis. *BMJ Open* 2020;**10**:e038505. https://doi.org/10.1136/bmjopen-2020-038505.

37 Ibekwe E, Haigh C, Duncan F, Fatoye F. Economic impact of routine opt-out antenatal human immune deficiency virus screening: A systematic review. *Journal of Clinical Nursing* 2017;**26**:3832–42.

38 Institute of Health E. First and Second Trimester Prenatal Screening for Trisomies 13, 18, and 21 and Open Neural Tube Defects. *Institute of Health Economics* 2012;**06**:9.

39 John NM, Wright SJ, Gavan SP, Vass CM. The role of information provision in economic evaluations of non-invasive prenatal testing: a systematic review. *European Journal of Health Economics* 2019;**20**:1123–31.

40 Kaambwa B, Bryan S, Gray J, Milner P, Daniels J, Khan KS, *et al.* Cost-effectiveness of rapid tests and other existing strategies for screening and management of early-onset group B streptococcus during labour. *BJOG: An International Journal of Obstetrics & Gynaecology* 2010;**117**:1616–27.

41 Karnon J, Goyder E, Tappenden P, McPhie S, Towers I, Brazier J, *et al.* A review and critique of modelling in prioritising and designing screening programmes. *Health Technology Assessment* 2007;**11**:iii–137.

42 Kobrynski L. Newborn screening for severe combined immune deficiency (technical and political aspects). *Current Opinion in Allergy and Clinical Immunology* 2015;**15**:539–46.

43 Krauth C. Health economic analysis of screening. *Gms Current Topics in Otorhinolaryngology Head and Neck Surgery* 2008;**7**:Doc01. https://doi.org/10.1542/peds.110.5.855. [35] Kemper AR, Downs SM. A cost-effectiveness analysis of newborn hearing screening strategies. Arch Pediatr Adolesc Med. 2000;154:484–488. [36] Kezirian EJ, White KR, Yueh B, Sullivan SD. Cost and cost-effectiveness of universal screening for hearing loss in newborns. Otolaryngol Head Neck Surg. 2001;124:359–364. doi: 10.1067/mhn.2001.113945. [43] Grill E, Uus K, Hessel F, Davies L, Taylor RS, Wasem J, Bamford J. Neonatal hearing screening: modelling cost and effectiven.

44 Kubiak C, Jyonouchi S, Kuo C, Garcia-Lloret M, Dorsey MJ, Sleasman J, *et al.* Fiscal implications of newborn screening in the diagnosis of severe combined immunodeficiency. *Journal of Allergy and Clinical Immunology in Practice* 2014;**2**:697–702.

45 Langer A, Holle R, John J. Specific guidelines for assessing and improving the methodological quality of economic evaluations of newborn screening. *BMC Health Services Research* 2012;**12**:300.

46 Langer A, John J. Newborn screening and health economics - A challenging relationship. *Monatsschrift Fur Kinderheilkunde* 2009;**157**:1230–6.

47 Lees CM, Davies S, Dezateux C. Neonatal screening for sickle cell disease. *Cochrane Database of Systematic Reviews* 2000:CD001913.

48 Lipstein EA, Vorono S, Browning MF, Green NS, Kemper AR, Knapp AA, *et al.* Systematic evidence review of newborn screening and treatment of severe combined immunodeficiency. *Pediatrics* 2010;**125**:e1226-35.

49 Lohse N, Marseille E, Kahn JG. Development of a model to assess the cost-effectiveness of gestational diabetes mellitus screening and lifestyle change for the prevention of type 2 diabetes mellitus. *International Journal of Gynaecology and Obstetrics* 2011;**115 Suppl**:S20-5.

50 Nargesi S, Rezapour A, Souresrafil A, Dolatshahi Z, Khodaparast F. Cost-Effectiveness Analysis of Pulse Oximetry Screening in the Full-Term Neonates for Diagnosis of Congenital Heart Disease: A Systematic Review. *Iranian Journal of Pediatrics* 2020;**30**:. https://doi.org/10.5812/ijp.105393.

51 Neyt M, Hulstaert F, Gyselaers W. Introducing the non-invasive prenatal test for trisomy 21 in Belgium: a cost-consequences analysis. *BMJ Open* 2014;**4**:e005922.

52 Nosratnejad S, Barfar E, Hosseini H, Barooti E, Rashidian A. Cost-effectiveness of anemia screening in vulnerable groups: A systematic review. *International Journal of Preventive Medicine* 2014;**5**:813–9.

53 Nshimyumukiza L, Menon S, Hina H, Rousseau F, Reinharz D. Cell-free DNA noninvasive prenatal screening for aneuploidy versus conventional screening: A systematic review of economic evaluations. *Clinical Genetics* 2018;**94**:3–21.

54 Pandor A, Eastham J, Chilcott J, Paisley S, Beverley C. Economics of tandem mass spectrometry screening of neonatal inherited disorders. *International Journal of Technology Assessment in Health Care* 2006;**22**:321–6.

55 Petrou S, Henderson J, Roberts T, Martin MA. Recent economic evaluations of antenatal screening: a systematic review and critique. *Journal of Medical Screening* 2000;**7**:59–73.

56 Pollitt RJ. Newborn mass screening versus selective investigation: benefits and costs. *Journal of Inherited Metabolic Disease* 2001;**24**:299–302.

57 Postma MJ, Sagoe KW, Dronkers F, Sprenger HG, de Jong- van den Berg L, Beck EJ. Cost-effectiveness of antenatal HIV-testing: reviewing its pharmaceutical and methodological aspects. *Expert Opinion on Pharmacotherapy* 2004;**5**:521–8.

58 Prosser LA, Grosse SD, Kemper AR, Tarini BA, Perrin JM. Decision analysis, economic evaluation, and newborn screening: challenges and opportunities. *Genetics in Medicine* 2013;**14**:703–12.

59 Roberts T, Henderson J, Mugford M, Bricker L, Neilson J, Garcia J. Antenatal ultrasound screening for fetal abnormalities: a systematic review of studies of cost and cost effectiveness. *BJOG: An International Journal of Obstetrics & Gynaecology* 2002;**109**:44–56.

60 Rochau U, Rushaj VQ, Schaffner M, Schonhensch M, Stojkov I, Jahn B, *et al.* Decision-Analytic Modeling Studies in Prevention and Treatment of Iodine Deficiency and Thyroid Disorders: A Systematic Overview. *Thyroid* 2020;**30**:746–58. https://doi.org/10.1089/thy.2018.0776.

61 Rose M, Myers J, Evans J, Prince A, Espinosa C. Hepatitis C virus risk-based vs. universal screening among pregnant women: Implementation and cost-effectiveness analysis. *Hepatology* 2018;**68 (Supple**:55A.

62 Saab S, Kullar R, Khalil H, Gounder P. Cost-effectiveness of Universal Hepatitis C Screening in Pregnant Women: A Systematic Review. *Journal of Clinical Gastroenterology* 2020. https://doi.org/10.1097/MCG.0000000000001360.

63 Santoro SL, Chicoine B, Jasien JM, Kim JL, Stephens M, Bulova P, *et al.* Pneumonia and respiratory infections in Down syndrome: A scoping review of the literature. *American Journal of Medical Genetics Part A* 2021;**185**:286–99. https://doi.org/10.1002/ajmg.a.61924.

64 Schmidt M, Werbrouck A, Verhaeghe N, De Wachter E, Simoens S, Annemans L, *et al.* Strategies for newborn screening for cystic fibrosis: A systematic review of health economic evaluations. *Journal of Cystic Fibrosis* 2018;**17**:306–15.

65 Scott DA, Loveman E, McIntyre L, Waugh N. Screening for gestational diabetes: a systematic review and economic evaluation. *Health Technology Assessment* 2002;**6**:1–161.

66 Sinkey RG, Odibo AO. Cost-Effectiveness of Old and New Technologies for Aneuploidy Screening. *Clinics in Laboratory Medicine* 2016;**36**:237–48.

67 Sparks TN, Caughey AB. How should costs and cost-effectiveness be considered in prenatal genetic testing? *Seminars in Perinatology* 2018;**42**:275–82. https://doi.org/10.1053/j.semperi.2018.07.003.

68 Therrell Jr. BL, Buechner C. Newborn screening for all identifiable disorders with tandem mass spectrometry is cost effective: supporting arguments. *Annals of the Academy of Medicine Singapore* 2008;**37**:32–4.

69 Tita AT, Grobman WA, Rouse DJ. Antenatal herpes serologic screening: an appraisal of the evidence. *Obstetrics and Gynecology* 2006;**108**:1247–53.

70 Tiwana SK, Rascati KL. Expanded newborn screening in Texas: A cost-effectiveness analysis using markov modeling. *Value in Health* 2009;**12 (3)**:A165.

71 Vande Velde S, Schillemans A, Van Biervliet S, Robberecht E. Neonatal screening for cystic fibrosis. *Tijdschrift Voor Geneeskunde* 2004;**60**:1217–24.

72 Waugh N, Royle P, Clar C, Henderson R, Cummins E, Hadden D, *et al.* Screening for hyperglycaemia in pregnancy: a rapid update for the National Screening Committee. *Health Technology Assessment* 2010;**14**:1–183.

73 Weile LK, Kahn JG, Marseille E, Jensen DM, Damm P, Lohse N. Global cost-effectiveness of GDM screening and management: current knowledge and future needs. *Best Practice and Research in Clinical Obstetrics and Gynaecology* 2015;**29**:206–24.

74 Wright SJ, Jones C, Payne K, Dharni N, Ulph F. The Role of Information Provision in Economic Evaluations of Newborn Bloodspot Screening: A Systematic Review. *Applied Health Economics & Health Policy* 2015;**13**:615–26.

75 Wu O, Robertson L, Twaddle S, Lowe G, Clark P, Walker I, *et al.* Screening for thrombophilia in high-risk situations: a meta-analysis and cost-effectiveness analysis. *British Journal of Haematology* 2005;**131**:80–90.

76 Zakiyah N, Postma MJ, Baker PN, van Asselt AD, Consortium IMpro. Pre-eclampsia Diagnosis and Treatment Options: A Review of Published Economic Assessments. *PharmacoEconomics* 2015;**33**:1069–82.

77 UK National Screening Committee. *Antenatal screening for Fragile X Syndrome*. 2010. URL: https://legacyscreening.phe.org.uk/policydb_download.php?doc=1168 (Accessed February 17, 2021).

78 UK National Screening Committee. *Repeat screening for syphilis in pregnancy: A cost-effectiveness model*. 2020. URL: https://legacyscreening.phe.org.uk/policydb_download.php?doc=1307 (Accessed February 17, 2021).

79 Chapple J. *Antenatal screening for Toxoplasmosis*. 2015. URL: https://legacyscreening.phe.org.uk/policydb_download.php?doc=1141 (Accessed February 17, 2021).

80 Knowles R, Hunter R. *Screening for Congenital Heart Defects*. 2014. URL: https://legacyscreening.phe.org.uk/documents/pulse-oximetry/CHD and PO First Review Doc.pdf (Accessed February 17, 2021).

81 Cartwright S. *An evaluation of carrier screening for spinal muscular atrophy against the National Screening Committee criteria*. 2012. URL: https://legacyscreening.phe.org.uk/policydb_download.php?doc=279 (Accessed February 17, 2021).

82 UK National Screening Committee. *Evaluation of Antenatal Screening for Thrombophilia against National Screening Committee Handbook Criteria, with consideration of neonatal screening and general population screening*. n.d. URL: https://legacyscreening.phe.org.uk/policydb_download.php?doc=1174 (Accessed February 17, 2021).

83 Wood P. *Vasa praevia and placenta praevia screening in pregnancy*. 2013. URL: https://legacyscreening.phe.org.uk/policydb_download.php?doc=1109 (Accessed February 17, 2021).

84 UK National Screening Committee. *Screening for Group B Streptococcal infection in pregnancy*. 2012. URL: https://legacyscreening.phe.org.uk/policydb_download.php?doc=499 (Accessed February 17, 2021).

85 Solutions for Public Health. *Screening for antenatal and postnatal mental health problems*. 2019. URL: https://legacyscreening.phe.org.uk/policydb_download.php?doc=1112 (Accessed February 17, 2021).

86 Waugh N, Royle P, Clar C, Henderson R, Cummins E, Hadden D, *et al.* Screening for hyperglycaemia in pregnancy: a rapid update for the National Screening Committee. *Health Technology Assessment* 2010;**14**:1–83.

87 Solutions for Public Health. *Antenatal screening for hepatitis C virus*. 2018. URL: https://legacyscreening.phe.org.uk/policydb_download.php?doc=1064 (Accessed February 17, 2021).

88 UK National Screening Committee. *Newborn screening for cytomegalovirus*. 2017. URL: https://legacyscreening.phe.org.uk/policydb_download.php?doc=1015 (Accessed February 17, 2021).

89 UK National Screening Committee. *Screening for Very Long Chain Acyl Coenzyme A Dehydrogenase Deficiency*. 2014. URL: https://legacyscreening.phe.org.uk/policydb_download.php?doc=1034 (Accessed February 17, 2021).

90 UK National Screening Committee. *Screening for Carnitine Transporter Deficiency*. 2014. URL: https://legacyscreening.phe.org.uk/policydb_download.php?doc=472 (Accessed February 17, 2021).

91 UK National Screening Committee. *Screening for Tyrosinaemia I*. 2014. URL: https://legacyscreening.phe.org.uk/policydb_download.php?doc=474 (Accessed February 17, 2021).

92 UK National Screening Committee. *Screening for Citrullinaemia and Argininosuccinate lyase deficiency*. 2014. URL: https://legacyscreening.phe.org.uk/policydb_download.php?doc=1160 (Accessed February 17, 2021).

93 Public Health England. *Recommendation to the UK National Screening Committee (UK NSC) for population screening for critical congenital heart disease and significant non-cardiac conditions using pulse oximetry screening in addition to current routine screening*. n.d. URL: https://legacyscreening.phe.org.uk/documents/pulse-oximetry/PO Research Review.pdf (Accessed February 17, 2021).

94 UK National Screening Committee. *Newborn screening for propionic acidaemia*. 2015. URL: https://legacyscreening.phe.org.uk/policydb_download.php?doc=1102 (Accessed February 17, 2021).

95 UK National Screening Committee. *Screening for Amino Acid Metabolism Disorders Disease*. 2015. URL: https://legacyscreening.phe.org.uk/policydb_download.php?doc=1161 (Accessed February 17, 2021).

96 Chilcott J, Bessey A, Pandor A, Paisley S. *Expanded newborn screening for inborn errors of the metabolism*. 2013. URL: https://legacyscreening.phe.org.uk/policydb_download.php?doc=416 (Accessed February 17, 2021).

97 UK National Screening Committee. *Screening for Severe Combined Immunodeficiency*. 2012. URL: https://legacyscreening.phe.org.uk/policydb_download.php?doc=1124 (Accessed February 17, 2021).

98 UK National Screening Committee. *Screening for Group B Streptococcal infection in pregnancy*. 2012. URL: https://legacyscreening.phe.org.uk/policydb_download.php?doc=1262 (Accessed February 17, 2021).

99 Hill C. *An evaluation of screening for postnatal depression against NSC criteria*. 2010. URL: https://legacyscreening.phe.org.uk/policydb_download.php?doc=140 (Accessed February 17, 2021).

100 Australian Government Department of Health. *Clinical Practice Guidelines: Pregnancy care (Economic analyses)*. 2018. URL: https://www.health.gov.au/sites/default/files/clinical-practice-guidelines-pregnancy-care-economic-analyses.pdf (Accessed February 17, 2021).

101 Ampersand Health Science. *Evidence evaluation report - Thyroid dysfunction*. 2017. URL: https://consultations.health.gov.au/health-services-division/antenatal-care-guidelines-review/supporting_documents/Thyroid dysfunction evidence evaluation 16May17.docx (Accessed February 17, 2021).

102 Medical Services Advisory Committee. *Genetic test for fragile X syndrome*. 2002. URL: http://www.health.gov.au/internet/msac/publishing.nsf/Content/EA0095D0692554F6CA25801000123B7D/$File/1035-Genetic-test-for-fragile-X-syndrome-Assessment-Report.pdf (Accessed February 17, 2021).

103 Medical Services Advisory Committee. *Application No. 1492 – Non-Invasive Prenatal Testing*. 2018. URL: https://www1.health.gov.au/internet/msac/publishing.nsf/Content/B1BCB2807D09C12DCA258258000F2025/$File/1492 - Final PSD.pdf (Accessed February 17, 2021).

104 Medical Services Advisory Committee. *Application No. 1467 – Obstetric MRI*. 2018. URL: https://www1.health.gov.au/internet/msac/publishing.nsf/Content/3809AECE10C4998CCA2580D5000F0B7C/$File/1467-Final_PSD.pdf (Accessed February 17, 2021).

105 Medical Services Advisory Committee. *Application No. 1573 – Reproductive carrier testing for cystic fibrosis, spinal muscular atrophy and fragile X syndrome*. 2020. URL: https://www1.health.gov.au/internet/msac/publishing.nsf/Content/4EF0E3C5A7CC9D05CA2584240009557E/$File/1573 - Final PSD_Jul2020.docx (Accessed February 17, 2021).

106 Medical Services Advisory Committee. *Application No. 1533 – Genome-wide microarray testing for pregnancies with major fetal structural abnormalities detected by ultrasound*. 2019. URL: https://www1.health.gov.au/internet/msac/publishing.nsf/Content/316E609CD3EC1E8DCA2583350000C6DA/$File/1533 Final PSD.pdf (Accessed February 17, 2021).

107 Medical Services Advisory Committee. *Application 1335 – Point of Care Tests to exclude preterm labour: Phosphorylated Insulin-like Growth Factor Binding Protein test*. 2014. URL: https://www1.health.gov.au/internet/msac/publishing.nsf/Content/4D7B4B53015CDBA1CA25801000123BDD/$File/1335-Final-PSD-accessible.docx (Accessed February 17, 2021).

108 Ampersand Health Science Writing. *Evidence evaluation report - Pre-eclampsia*. 2017. URL: https://consultations.health.gov.au/health-services-division/antenatal-care-guidelines-review/supporting_documents/Preeclampsia evaluation report 16May17.pdf (Accessed February 17, 2021).

109 Medical Services Advisory Committee. *Application No. 1216 – Cystic fibrosis transmembrane regulator (CFTR) testing*. 2015. URL: https://www1.health.gov.au/internet/msac/publishing.nsf/Content/D54C11627FEA9785CA25801000123BDC/$File/1216-FinalPSD-CFTR-accessible.docx (Accessed February 17, 2021).

110 Parrella A, Hiller J, Mundy L. *Screening and treatment of Gestational Diabetes Mellitus*. 2005. URL: https://www.health.gov.au/internet/horizon/publishing.nsf/Content/87861CF6758FF75ACA2575AD0080F348/$File/Gestational Diabetes December2005.pdf (Accessed February 17, 2021).

111 Medical Services Advisory Committee. *Application No. 1216.1 – Cystic Fibrosis Transmembrane Regulator (CFTR) testing*. 2017. URL: https://www1.health.gov.au/internet/msac/publishing.nsf/Content/4C5CB83B16D72E3FCA25815D001BFB37/$File/1216.1-FinalPSD-accessible.docx (Accessed February 17, 2021).

112 Mundy L, Merlin T. *Ultrasound screening for hip dysplasia: A new screening programme for the early detection of hip dysplasia in neonates*. 2003. URL: https://www1.health.gov.au/internet/horizon/publishing.nsf/Content/0F73968BFE0DC379CA2575AD0080F2E6/$File/v2_2.rtf (Accessed February 17, 2021).

113 Australian Government Department of Health. *Group B streptococcus*. n.d. URL: https://consultations.health.gov.au/phd-tobacco/antenatal-care_module-ii/user_uploads/7.5group-b-streptococcus.docx (Accessed February 17, 2021).

114 Australian Government Department of Health. *Newborn Bloodspot Screening Condition Assessment Summary: Congenital Adrenal Hyperplasia (CAH)*. n.d. URL: https://www.health.gov.au/sites/default/files/documents/2020/02/newborn-bloodspot-screening-condition-assessment-summary-congenital-adrenal-hyperplasia_0.pdf (Accessed February 17, 2021).

115 Australian Government Department of Health. *Haemoglobin disorders*. n.d. URL: https://consultations.health.gov.au/phd-tobacco/antenatal-care_module-ii/user_uploads/7.2haemoglobin-disorders.docx (Accessed February 17, 2021).

116 Medical Services Advisory Committee. *Genetic test for fragile X syndrome*. 2002. URL: https://www1.health.gov.au/internet/msac/publishing.nsf/Content/EA0095D0692554F6CA25801000123B7D/$File/1035-Genetic-test-for-fragile-X-syndrome-One-page-Summary.pdf%0A (Accessed February 17, 2021).

117 Gerkens S, Martin N, Thiry N, Hulstaert F. *Hepatitis C: Screening en Preventie*. 2011. URL: https://kce.fgov.be/sites/default/files/atoms/files/KCE_173A_hepatitis_C_2.pdf (Accessed February 17, 2021).

118 Government of Canada. *Why is it important to address Fetal Alcohol Spectrum Disorder (FASD)?*. 2007. URL: https://www.canada.ca/content/dam/phac-aspc/migration/phac-aspc/hp-ps/dca-dea/prog-ini/fasd-etcaf/publications/pdf/factsheet2-fasd-etcaf-eng.pdf (Accessed February 17, 2021).

119 Canadian Task Force on Preventive Health Care. *Prevention of Early-onset Group B Streptococcal (GBS) Infection in the Newborn: Systematic Review and Recommendations*. 2001. URL: https://canadiantaskforce.ca/wp-content/uploads/2016/09/2002-streptococcal-systematic-review-and-recommendations-en.pdf (Accessed February 17, 2021).

120 Medical Advisory Secretariat. *Neonatal Screening of Inborn Errors of Metabolism Using Tandem Mass Spectrometry: An Evidence-Based Analysis*. 2003. URL: https://www.hqontario.ca/Portals/0/Documents/evidence/reports/rev_tandms_090102.pdf (Accessed February 17, 2021).

121 Turcotte C, Blancquaert I, St-Louis M. *Évaluation de la pertinence du dépistage néonatal sanguin de la galactosémie classique (GALT)*. 2020. URL: https://www.inesss.qc.ca/fileadmin/doc/INESSS/Rapports/Depistage/INESSS_DepistageNeonatal_GALT.pdf (Accessed February 17, 2021).

122 Brunet J, Blancquaert I, Lalancette-Hébert M, St-Louis M. *Évaluation de la pertinence du dépistage néonatal sanguin du déficit en biotinidase (BIOT)*. 2020. URL: https://www.inesss.qc.ca/fileadmin/doc/INESSS/Rapports/Depistage/INESSS_DepistageNeonatal_Biotinidase.pdf (Accessed February 17, 2021).

123 Brunet J, St-Louis M, Blancquaert I. *Évaluation de la pertinence du dépistage néonatal sanguin par spectrométrie de masse en tandem du déficit en bêta-cétothiolase (βKT)*. 2020. URL: https://www.inesss.qc.ca/fileadmin/doc/INESSS/Rapports/Depistage/INESSS_DepistageNeonatal_BKT.pdf (Accessed February 17, 2021).

124 Institut national d’excellence en santé et en services sociaux (INESS). *Assessment of the relevance of tandem mass spectrometry-based newborn blood spot screening for carnitine uptake deficiency (CUD)*. 2020. URL: https://www.inesss.qc.ca/fileadmin/doc/INESSS/Rapports/Depistage/INESSS_Neonatal_CUD_Summary.pdf (Accessed February 17, 2021).

125 Létourneau I, Blancquaert I, Lalancette-Hébert M, Brunet J. *Évaluation de la pertinence du dépistage néonatal sanguin par spectrométrie de masse en tandem de l’acidémie isovalérique (IVA)*. 2020. URL: https://www.inesss.qc.ca/fileadmin/doc/INESSS/Rapports/Depistage/INESSS_DepistageNeonatal_IVA.pdf%0A (Accessed February 17, 2021).

126 Létourneau I, Blancquaert I, Brabant J, Lalancette-Hébert M. *Évaluation de la pertinence du dépistage néonatal sanguin par spectrométrie de masse en tandem du défaut de captation de la carnitine cellulaire (CUD)*. 2020. URL: https://www.inesss.qc.ca/fileadmin/doc/INESSS/Rapports/Depistage/INESSS_DepistageNeonatal_CUD.pdf%0A (Accessed February 17, 2021).

127 Makni H, St-Hilaire C, Robb L, K L, I B. *Spectrométrie de masse en tandem et dépistage néonatal des erreurs innées du métabolisme*. 2007. URL: https://www.inesss.qc.ca/fileadmin/doc/AETMIS/Rapports/DepistageGenetique/2007_03_Mono.pdf%0A (Accessed February 17, 2021).

128 Côté B, Gosselin C. *Pertinence d’élargir le programme de dépistage néonatal sanguin au Québec*. 2013. URL: https://www.inesss.qc.ca/fileadmin/doc/INESSS/Rapports/Genetique/INESSS_Depistage_neonatal_sanguin.pdf%0A (Accessed February 17, 2021).

129 Agence d’évaluation des technologies et des modes d’intervention en santé (AETMIS). *La spectrométrie de masse en tandem et le dépistage néonatal sanguin au Québec*. 2007. URL: https://www.inesss.qc.ca/fileadmin/doc/AETMIS/Rapports/DepistageGenetique/ETMIS2007_Vol.3_No3.pdf%0A (Accessed February 17, 2021).

130 Brunet J, Blancquaert I, St-Louis M. *Évaluation de la pertinence du dépistage néonatal sanguin par spectrométrie de masse en tandem du déficit en holocarboxylase synthétase (HCS)*. 2020. URL: https://www.inesss.qc.ca/fileadmin/doc/INESSS/Rapports/Depistage/INESSS_DepistageNeonatal_HCS.pdf%0A (Accessed February 17, 2021).

131 Institut national d’excellence en santé et en services sociaux (INESSS). *Détection des aneuploïdies des chromosomes 13, 18, 21, X et y par QF-PCR*. 2013. URL: https://www.inesss.qc.ca/fileadmin/doc/INESSS/Analyse_biomedicale/Avril_2014/Detection_aneuploidies_chromosomes_13-18-21-X_Y_par_QF-PCR.pdf%0A (Accessed February 17, 2021).

132 Hvas A, Ehlers L, Møller H. *Screening af gravide indvandrere for hæmoglobinopati – en medicinsk teknologivurdering*. 2009. URL: https://www.sst.dk/-/media/Udgivelser/2009/Publ2009/MTV/haemoglobinopati/Screening_gravide_indvandrere_net_final,-d-,pdf.ashx%0A (Accessed February 17, 2021).

133 Gemeinsamer Bundesausschuss. *Kinder-Richtlinie: Screening von Neugeborenen zur Früherkennung von SCID*. 2019. URL: https://www.g-ba.de/downloads/40-268-5426/2018-11-22_Kinder-RL_SCID-Screening_ZD.pdf (Accessed February 17, 2021).

134 Gemeinsamer Bundesausschuss. *Neugeborenen-Hörscreening*. 2008. URL: https://www.g-ba.de/downloads/40-268-759/2008-12-17-Abschluss-Hörscreening.pdf (Accessed February 17, 2021).

135 Lawrenson R. *NSAC “Statement of Advice”: Should women be screened for vitamin D during pregnancy in New Zealand?* 2010. URL: https://www.nsu.govt.nz/system/files/page/vitamin-d-screening-during-pregnancy.pdf (Accessed February 17, 2021).

136 Sherwood J. *Chlamydia Screening in New Zealand: Report for the National Screening Unit*. 2006. URL: https://www.nsu.govt.nz/system/files/resources/chlamydia-screening-report.pdf (Accessed February 17, 2021).

137 Williams L, Jackson G. *Screening for SCID - Literature Review*. 2013. URL: https://www.nsu.govt.nz/system/files/resources/screening-severe-combined-immune-deficiency-literature-review.pdf (Accessed February 17, 2021).

138 Lawrenson R. *Gestational Diabetes Mellitus: Should GPs keep a register of everyone with GDM?*. 2010. URL: https://www.nsu.govt.nz/system/files/page/gestational-diabetes-gp-conf-2010-r-lawrenson.pdf (Accessed February 17, 2021).

139 Gjertsen MK, Johansen M, Movik E, Norderhaug IN. *Forebygging av infeksjon med gruppe B-streptokokker i nyfødtperioden*. 2006. URL: https://www.fhi.no/globalassets/dokumenterfiler/notater/2006/notat_06_gbs.pdf (Accessed February 17, 2021).

140 Dorenberg DH, Greve-Isdahl M, Nøkleby H. *Kunnskapsoppsummering: Hepatitt B-undersøkelser i svangerskapet*. 2017. URL: https://www.fhi.no/globalassets/dokumenterfiler/tema/hepatitt/kunnskapsoppsummering-om-hepatitt-b-undersokelser-i-svangerskapet.pdf (Accessed February 17, 2021).

141 Lin JS, Eder M, Bean S. *Screening for Syphilis Infection in Pregnant Women: A Reaffirmation Evidence Update for the U.S. Preventive Services Task Force*. 2018. URL: https://www.uspreventiveservicestaskforce.org/Home/GetFile/1/16875/syphilis-screening-pregnancy-final-evidence-review/pdf (Accessed February 17, 2021).

142 Glass N, Nelson HD, Huffman L. *Screening for Genital Herpes Simplex: Brief Update for the U.S. Preventive Services Task Force*. 2005. URL: https://www.uspreventiveservicestaskforce.org/Home/GetFile/1/733/herpesup/pdf (Accessed February 17, 2021).

143 US Preventive Services Task Force. *Screening for Genital Herpes*. 2005. URL: https://www.uspreventiveservicestaskforce.org/Home/GetFile/1/735/herpesrs/pdf (Accessed February 17, 2021).

144 US Preventive Services Task Force. *Screening for Syphilis Infection*. 2004. URL: https://www.uspreventiveservicestaskforce.org/home/getfilebytoken/qVACAf9sMmuX48MnnVDcBR (Accessed February 17, 2021).

145 Moyer V. Screening for HIV: US preventive services task force recommendation statement. *Annals of Internal Medicine* 2013;**159**:51–60.

146 Donovan L, Hartling L, Muise M, Guthrie A, Vandermeer B, Dryden D. Screening tests for gestational diabetes: a systematic review for the US Preventive Services Task Force. *Annals of Internal Medicine* 2013;**159**:115–22.

147 Berg A, Allan J, Calonge N, Frame P. Screening for HIV: Recommendation Statement. *Annals of Internal Medicine* 2005;**143**:32.

148 Lin K, Barton M. *Screening for Hemoglobinopathies in Newborns: Reaffirmation Update for the U.S. Preventive Services Task Force*. 2007. URL: https://www.uspreventiveservicestaskforce.org/Home/GetFile/1/1051/sicklecelles/pdf (Accessed February 17, 2021).

149 Glass N, Nelson H, Villemyer K. *Screening for Gonorrhea: Update of the Evidence*. 2005. URL: http://www.uspreventiveservicestaskforce.org/Home/GetFile/1/609/gonup/pdf (Accessed February 17, 2021).

150 Shipman SA, Helfand M, Moyer VA, Yawn BP. *Screening for Developmental Dysplasia of the Hip: A Systematic Literature Review for the U.S. Preventive Services Task Force*. n.d. URL: https://www.uspreventiveservicestaskforce.org/home/getfilebytoken/V3HhneZFw__Vk7L8gwryw3 (Accessed February 17, 2021).

151 Chou R, Cottrell E, Wasson N, Rahman B, Guise J-M. *Screening for Hepatitis C Virus Infection in Adults*. 2012. URL: https://www.uspreventiveservicestaskforce.org/Home/GetFile/1/668/cerhepcscr/pdf (Accessed February 17, 2021).

152 Nelson H, Glass N, Huffman L, Villemyer K, Hamilton A, Frame P, *et al.* *Screening for Syphilis: Brief Update for the U.S. Preventive Services Task Force*. 2004. URL: https://www.uspreventiveservicestaskforce.org/Home/GetFile/1/991/syphilup/pdf (Accessed February 17, 2021).

153 Hayes I. *Ultrasonography (Ultrasound) in Pregnancy*. 2010. URL: https://www.hca.wa.gov/assets/program/final_report_ultrasound.pdf (Accessed February 17, 2021).

154 Cheng E. *Cell-free DNA prenatal screening for chromosomal aneuploidies*. 2020. URL: https://www.hca.wa.gov/assets/program/htcc-materials-cfdna-20200117.pdf (Accessed February 17, 2021).

155 Center for Evidence-based Policy Oregon Health & Science University. *Cell-free DNA Prenatal Screening for Chromosomal Aneuploidies - Draft key questions: public comment and response*. 2019. URL: https://www.hca.wa.gov/assets/program/cell-free-dna-drft-key-qs-comment-response-20190826.pdf (Accessed February 17, 2021).

156 Charis Management Consulting I. *Review of Newborn Screening for Inborn Errors of Metabolism and Cystic Fibrosis: Synthesis Report*. 2006. URL: https://open.alberta.ca/dataset/24724e5c-2b9a-4a25-a2ac-bfd0678fc9fb/resource/6df326d0-b6cb-4d46-b936-4f999d020f74/download/3839488-2006-review-of-newborn-screening-for-inborn-errors-synthesis-report.pdf (Accessed February 17, 2021).

157 The Health Technology Assessment Unit University of Calgary. *Hepatitis C Screening in Alberta*. 2016. URL: https://open.alberta.ca/dataset/94508e51-c9ae-4777-971f-3484860f7ede/resource/cbb2a13f-2a0f-428d-b3e2-5af1c6d514d1/download/ahtdp-hepatitisc-screening-hta-report-2016.pdf (Accessed February 17, 2021).

158 Canadian Agency for Drugs and Technologies in Health. *Non-invasive Prenatal Testing: A Review of the Cost Effectiveness and Guidelines*. 2014. URL: https://www.cadth.ca/sites/default/files/pdf/htis/apr-2014/RC0520-NIPT-Final.pdf (Accessed February 17, 2021).

159 Canadian Agency for Drugs and Technologies in Health. *Screening for Hepatitis C: A Review*. 2017. URL: https://www.cadth.ca/sites/default/files/pdf/HT0014_HepC_InBrief_e.pdf (Accessed February 17, 2021).

160 Canadian Agency for Drugs and Technologies in Health. *Thrombophilia Testing for Women Who Have Had Pregnancy Loss: Clinical Evidence, Cost-effectiveness, and Guidelines*. 2014. URL: https://www.cadth.ca/media/pdf/htis/dec-2014/RA0693 Thrombophilia in pregnancy Final.pdf (Accessed February 17, 2021).

161 Canadian Agency for Drugs and Technologies in Health. *Newborn Screening for Krabbe Leukodystrophy: A Review of the Clinical and Cost Effectiveness and Guidelines*. 2012. URL: https://www.cadth.ca/sites/default/files/pdf/htis/feb-2012/RC0328 Newborn screening for Krabbe Final.pdf (Accessed February 17, 2021).

162 Dziegiel M, Hedegaard M, Madsen C, Nilsson C, Pedersen H, Prahm K. *Føtal og neonatal alloimmun trombocytopeni (FNAIT): Gennemgang af den foreliggende litteratur med henblik på at forberede en national screenings indsats*. 2016. URL: https://www.dsog.dk/s/161218_FNAITP_guideline_foerste_udkast.pdf (Accessed February 17, 2021).

163 International Network of Agencies for Health Technology Assessment. *INAHTA Briefs Compilation 2009-2010*. 2010. URL: http://www.inahta.org/upload/Briefs_10/INAHTA Briefs Compilations volume 10.pdf (Accessed February 17, 2021).

164 International Network of Agencies for Health Technology Assessment. *INAHTA Briefs Compilation 2007-2008*. 2008. URL: https://www.inahta.org/upload/Briefs_8/INAHTA_Briefs_Compilation_8.pdf (Accessed February 17, 2021).

165 Canadian Agency for Drugs and Technologies in Health. *Screening for Chlamydia Trachomatis and Neisseria Gonorrhoeae During Pregnancy: A Health Technology Assessment*. 2019. URL: https://www.inahta.org/upload/2019/19017_Screening for Chlamydia Trachomatis and Neisseria Gonorrhoeae During Pregnancy.pdf (Accessed February 17, 2021).

166 Ritchie, K; Quinn S. *Routine Ultrasound Scanning Before 24 Weeks of Pregnancy*. 2004. URL: https://www.inahta.org/upload/Briefs_4/0417_NHSQIS.pdf (Accessed February 17, 2021).

167 International Network of Agencies for Health Technology Assessment. *INAHTA Briefs Compilation 2001-2002*. 2002. URL: https://www.inahta.org/upload/Briefs_2/INAHTA_Briefs_Compilation_2.pdf (Accessed February 17, 2021).

168 Mohamad N. *Hepatitis B and Hepatitis C Screening Among High Risk Groups*. 2018. URL: https://www.inahta.org/upload/2018/18033_Hepatitis B and Hepatitis C Screening Among High Risk Groups.pdf (Accessed February 17, 2021).

169 International Network of Agencies for Health Technology Assessment. *INAHTA Briefs Compilation 2002-2003*. 2003. URL: https://www.inahta.org/upload/Briefs_3/INAHTA_Briefs_Compilation_3.pdf (Accessed February 17, 2021).

170 Greer I. *Screening for Thrombophilia in High-Risk Situations: Systematic Review and Cost-Effectiveness Analysis*. 2006. URL: https://www.inahta.org/upload/Briefs_7/06118_NCCHTA_Thrombophilia_HighRisk_Situations.pdf (Accessed February 17, 2021).

171 Estrada M, Guillén M, Iruretagoiena M, Taboada J, López de Argumedo M, Lapuente J, *et al.* *Description of the Current Status of Prenatal Screening in the Most Frequent Fetal Chromosopathies, Mainly Down Syndrome in Spain, and Proposals for the Improvement of Everyday Clinical Practice*. 2009. URL: http://www.inahta.org/upload/Briefs_9/09002_CAHTA_Description_Current_Status_Prenatal_Screening_Most_Frequent_Foetal_Chromosopathies.pdf (Accessed February 17, 2021).

172 International Network of Agencies for Health Technology Assessment. *INAHTA Briefs Compilation 2010-2011*. 2011. URL: http://www.inahta.org/upload/Briefs_11/1_Briefs Compilation 11_2.pdf (Accessed February 17, 2021).

173 International Network of Agencies for Health Technology Assessment. *INAHTA Briefs Compilation 2006-2007*. 2007. URL: https://www.inahta.org/upload/Briefs_7/INAHTA_Briefs_Compilation_7.pdf (Accessed February 17, 2021).

174 Darus N. *Thyroid Screening in Pregnant Women*. 2009. URL: https://www.inahta.org/upload/Briefs_10/09086_MaHTAS_Thyroid_Screening _Pregnant_Women.pdf (Accessed February 17, 2021).

175 Institute of Health Economics. *The safety and efficacy/effectiveness of using automated testing devices for universal newborn hearing screening: an update*. 2012. URL: http://www.inahta.org/upload/Briefs_12/12026_The_safety_and_efficacy_effectiveness_of_using_automated_testing_devices_for_universal_newborn_hearing_screening.pdf (Accessed February 17, 2021).

176 Wald N. *First and Second Trimester Antenatal Screening for Down’s Syndrome: The Results of the Serum, Urine and Ultrasound Screening Study (SURUSS)*. 2003. URL: https://www.inahta.org/upload/Briefs_4/0343_NCCHTA_Downs_Syndrome_SURUSS.pdf (Accessed February 17, 2021).

177 Institute of Health Economics. *First and second trimester prenatal screening update*. 2014. URL: http://www.inahta.org/upload/2014/14043_IHE_First and second trimester prenatal screening update.pdf (Accessed February 17, 2021).

178 Bricker L. *Ultrasound Screening in Pregnancy: A Systematic Review of the Clinical Effectiveness, Cost Effectiveness and Women’s Views*. 2002. URL: http://www.inahta.org/upload/Briefs_3/02-66 NCCHTA - Ultrasound Screening in Pregnancy A Systematic Review of the Clinical Effectiveness, Cost Effectiveness and Women’s Views.pdf (Accessed February 17, 2021).

179 Pandor A. *Clinical Effectiveness and Cost Effectiveness of Neonatal Screening for Inborn Errors of Metabolism Using Tandem Mass Spectrometry: A Systematic Review*. 2004. URL: http://www.inahta.org/upload/Briefs_5/0479_NCCHTA_Neonatal_Screening_Inborn_Errors_Metabolism_Tandem_Mass_Spectrometry.pdf (Accessed February 17, 2021).

180 Institute of Health Economics. *Transcutaneous bilirubinometry for the screening of hyperbilirubinemia in neonates ≥35 weeks’ gestation*. 2013. URL: http://www.inahta.org/upload/2013/13041_IHE_Transcutaneous bilirubinometry for the screening of hyperbilirubinemia in neonates.pdf (Accessed February 17, 2021).

181 Campbell S. *Screening for Postnatal Depression Within the Well Child Tamariki Ora Framework: An Economic Analysis of Implementation of a Screening Program*. 2008. URL: http://www.inahta.org/upload/Briefs_9/08094_HSAC_Screening_Postnatal_Depression_Within_Well_Child_Tamariki_Ora_Framework_Economic_Analysis_Implementation_Screening_Program.pdf (Accessed February 17, 2021).

182 de Wit G, Verweij A, van Baal P, Vijgen S, van den Berg M, Busch M, *et al.* *Economic evaluation of prevention: further evidence*. n.d. URL: https://www.rivm.nl/bibliotheek/rapporten/270091004.pdf (Accessed February 17, 2021).

183 Beraadsgroep Genetica van de Gezondheidsraad. *Neonatale screening op cystic fibrosis*. 2010. URL: http://www.rivm.nl/sites/default/files/2018-11/GRadviesCF_201001%2Cmaart 2010.pdf (Accessed February 17, 2021).

184 van Gils P, Tariq L, Hamberg-van Reenen H, van den Berg M. *Kosteneffectiviteit van preventie*. 2009. URL: https://www.rivm.nl/bibliotheek/rapporten/270091009.pdf (Accessed February 17, 2021).

185 Luc-Murk J, Wiersma T, Koelewijn J, van Leeuwen L, Jacobs P, Limburg Z, *et al.* *Rubellascreeningsbeleid bij zwangere vrouwen*. 2017. URL: https://lci.rivm.nl/sites/default/files/2017-06/Rubellascreeningsbeleid bij zwangere vrouwen LCI mei 2016.pdf (Accessed February 17, 2021).

186 Struijs J, de Wit G, Jager J. *Literatuuronderzoek naar kosten-effectiviteits-aspecten van de screening van zwangeren op syfillis ter preventie van congenitale syfillis*. n.d. URL: http://rivm.nl/bibliotheek/rapporten/403505007.pdf%0A (Accessed February 17, 2021).

187 van de Laar M, Beuker R, Rijlaarsdam J, van Duynhoven Y. *SOA en AIDS in Nederland*. 2000. URL: https://www.rivm.nl/bibliotheek/rapporten/441500011.pdf (Accessed February 17, 2021).

188 Statens beredning för medicinsk utvärdering. *Metoder för tidig fosterdiagnostik: En systematisk litteraturöversikt*. 2006. URL: https://www.sbu.se/contentassets/5c2bae2f615f4f3b91d9e08dfcbf7f64/fosterdiagnostik_fulltext.pdf (Accessed February 17, 2021).

189 Statens beredning för medicinsk utvärdering. *Blodprov för tidig upptäckt av Downs syndrom*. 2000. URL: https://www.sbu.se/contentassets/992e0260f40f459aa9dd4575eadfb4d2/blodprov_tidig_upptackt_downs_syndrom_2000.pdf (Accessed February 17, 2021).

190 National Institute for Health and Care Excellence. *High-throughput, non-invasive prenatal testing (NIPT) for fetal rhesus D status*. 2015. URL: https://www.nice.org.uk/guidance/dg25/documents/final-scope (Accessed February 17, 2021).

191 National Institute for Health and Care Excellence. *Twin and Triplet Pregnancy: [C] Evidence review for ultrasound screening for twin anaemia polycythaemia sequences*. 2019. URL: https://www.nice.org.uk/guidance/ng137/evidence/c-ultrasound-screening-for-twin-anaemia-polycythaemia-sequences-pdf-6898605375 (Accessed February 17, 2021).

192 National Institute for Health and Care Excellence. *Routine antenatal anti-D prophylaxis for RhD-negative women (review of technology appraisal guidance 41)*. 2008. URL: https://www.nice.org.uk/guidance/ta156/documents/pregnancy-rhesus-negative-women-routine-antid-review-overview2 (Accessed February 17, 2021).

193 National Institute for Health and Care Excellence. *Appendix A: summary of new evidence from 10-year surveillance of Postnatal care up to 8 weeks after birth (2006) NICE guideline CG37*. 2006. URL: https://www.nice.org.uk/guidance/cg37/evidence/appendix-a-summary-of-new-evidence-pdf-2736100910 (Accessed February 17, 2021).

194 Scott Shipman, S; Helfand, M; Nygren, P; Bougatsos C. *Screening for Developmental Dysplasia of the Hip*. 2006. URL: https://www.ahrq.gov/downloads/pub/prevent/pdfser/hipdyssyn.pdf (Accessed February 17, 2021).

195 Institute of Health Economics. *Transcutaneous Bilirubinometry for the Screening of Hyperbilirubinemia in Neonates ≥35 Weeks’ Gestation*. 2013. URL: https://www.ihe.ca/download/transcutaneous_bilirubinometry_for_the_screening_of_neonatal_hyperbilirubinemia.pdf (Accessed February 17, 2021).

196 Brunet, J; Blancquaert, I; St-Louis M. *Évaluation de la pertinence du dépistage néonatal sanguin par spectrométrie de masse en tandem de l’acidurie 3-hydroxy-3 méthylglutarique (HMG)*. 2020. URL: https://www.inesss.qc.ca/fileadmin/doc/INESSS/Rapports/Depistage/INESSS_DepistageNeonatal_HMG.pdf (Accessed February 17, 2021).

197 Gemeinsame Bundesausschuss. *zum Beschluss des Gemeinsamen Bundesausschusses über eine Änderung der Kinder-Richtlinie: Screening von Neugeborenen zur Früherkennung von SCID*. 2018. URL: https://www.g-ba.de/downloads/40-268-5425/2018-11-22_Kinder-RL_SCID-Screening_TrG.pdf (Accessed February 17, 2021).

198 Unterausschuss Methodenbewertung. *Screening auf schwere congenitale Herzfehler mittels Pulsoxymetrie nach*. 2012. URL: https://www.g-ba.de/downloads/40-268-2400/2012-11-22_Einleitung-Beratungsverfahren-Pulsoxymetrie_Antrag-PatV.pdf (Accessed February 17, 2021).

199 Socialstyrelsen. *Screening för svår kombinerad immunbrist: Rekommendation och bedömningsunderlag*. 2019. URL: https://www.socialstyrelsen.se/globalassets/sharepoint-dokument/artikelkatalog/nationella-screeningprogram/2019-7-6225.pdf (Accessed February 17, 2021).

200 van den Berg, M; van Baal, PHM; de Wit, GA; Schuit A. *Kosteneffectiviteit van preventie: Literatuursignalering en modellering*. 2008. URL: https://www.rivm.nl/bibliotheek/rapporten/270091007.pdf (Accessed February 17, 2021).

201 Kauffman-de Boer, M; de Ridder Sluiter, H; Schuitema, T; Uilenburg, N; Vinks, E; van der Ploeg, K; Lanting, C; Oudshoorn, K; Verkerk P. *Implementatiestudie Neonatale Gehoorscreening*. 2001. URL: https://www.rivm.nl/sites/default/files/2018-11/Voorstudies implementatie neonatale gehoorscreening.pdf (Accessed February 17, 2021).

202 Ahya R, Turner ML, Urbaniak SJ, Snait Study T. Fetomaternal alloimmune thrombocytopenia. *Transfusion and Apheresis Science* 2001;**25**:139–45.

203 Alzarea A, Alolayan S, Almutairi H, Alqahtani S, Rittenhouse B. Re-Evaluating The Cost-Effectiveness of Screening For Congenital Adrenal Hyperplasia (CAH): The Sensitivity to Choice of Distributions In Probabilistic Sensitivity Analyses (PSAS). *Value in Health* 2015;**18**:A668.

204 Anderssen SH, Andresen J, Andersen R, Sponheim L. Universal neonatal hearing screening of infants with otoacoustic emissions. *Tidsskrift for Den Norske Laegeforening* 2002;**122**:2187–9.

205 Anonymous. Group B strep in pregnancy: Test-and-treat, or just treat? *Journal of Family Practice* 2008;**57**:11.

206 Bassett K, Lee PM, Green CJ, Mitchell L, Kazanjian A. Improving population health or the population itself? Health technology assessment and our genetic future. *International Journal of Technology Assessment in Health Care* 2004;**20**:106–14.

207 Bestwick J, Wald N. Cost and efficacy comparison of prenatal recall and reflex DNA screening for trisomy 21, 18 and 13. *PLoS ONE* 2019;**14**:e0220053.

208 Buser K, Bietenduwel A, Krauth C, Jalilvand N, Meyer S, Reuter G, *et al.* Model project of hearing screening in new-born in Hanover (preliminary results). *Gesundheitswesen* 2003;**65**:200–3.

209 Butt K, Crane J, Hutcheon J, Lim K, Nevo O. Universal Cervical Length Screening. *Journal of Obstetrics and Gynaecology Canada* 2019;**41**:363–74.

210 Cao-Nguyen MH, Kos MI, Guyot JP. Benefits and costs of universal hearing screening programme. *International Journal of Pediatric Otorhinolaryngology* 2007;**71**:1591–5.

211 Chaves F, Smith S, Xu DS. Universal screening for G6PD deficiency in pregnant women is not cost-effective. *American Journal of Clinical Pathology* 2004;**122**:634–5.

212 Clement MC, Mahlaoui N, Mignot C, Le Bihan C, Rabetrano H, Hoang L, *et al.* Systematic neonatal screening for severe combined immunodeficiency and severe T-cell lymphopenia: Analysis of cost-effectiveness based on French real field data. *Journal of Allergy and Clinical Immunology* 2015;**135**:1589–93.

213 Coop C, Edlin R, Brown J, Farquhar C. Cost-effectiveness of the New Zealand diabetes in pregnancy guideline screening recommendations. *BMJ Open* 2015;**5**:e006996.

214 Coskun B, Gulumser C, Coskun B, Artuk C, Karasahin KE. Impact of Syrian refugees on congenital TORCH infections screening in Turkey. *Journal of Obstetrics and Gynaecology Research* 2020;**46**:1017–24. https://doi.org/10.1111/jog.14273.

215 Crimmins S, Liu X, Doyle L, Harman C, Turan O. Universal QUAD screen versus universal cell free DNA testing for Down’s syndrome screening: Cost-effectiveness analysis. *American Journal of Obstetrics and Gynecology* 2016;**214**:S381–2.

216 Cunningham L, Phipps H. A literature review and cost analysis comparing effectiveness of two Group B Streptococcus screening methods in term pregnancies. *Women and Birth* 2017;**30**:24. https://doi.org/10.1016/j.wombi.2017.08.062.

217 Ensari T, Kirbas A, Ozgu-Erdinc AS, Gokay Saygan S, Erkaya S, Uygur D, *et al.* An eight-year retrospective analysis of antenatal screening results for syphilis: is it still cost effective? *Journal of Infection in Developing Countries* 2015;**9**:1011–5.

218 Evans M, Krantz D, Hallahan T, Carmichael J. Combined first trimester screening for Down syndrome with nasal bone (NB) is cost advantageous over NIPS in younger advanced maternal age (AMA) patients. *American Journal of Obstetrics and Gynecology* 2014;**210**:S99.

219 Evans MI, Sonek JD, Hallahan TW, Krantz DA. Cell-free fetal DNA screening in the USA: a cost analysis of screening strategies. *Ultrasound in Obstetrics and Gynecology* 2015;**45**:74–83.

220 Ferrier C, Khoshnood B, Dhombres F, Randrianaivo H, Perthus I, Jouannic JM, *et al.* Cost and outcomes of the ultrasound screening program for birth defects over time: a population-based study in France. *BMJ Open* 2020;**10**:e036566. https://doi.org/10.1136/bmjopen-2019-036566.

221 Galderisi A, Lolli E, Cavicchiolo ME, Bonadies L, Trevisanuto D, Baraldi E. The aftermath of SARS-CoV-2 in NICU: saving or checking accounts? Projected cost-effectiveness analysis. *European Journal of Pediatrics* 2021. https://doi.org/10.1007/s00431-020-03884-1.

222 Gogou M, Evangeliou A. Is Metabolic Screening Necessary in Children with Autism Spectrum Disorder? A Mini Review. *Journal of Pediatric Neurology* 2019;**17**:199–205.

223 Gorga MP, Neely ST. Cost-effectiveness and test-performance factors in relation to universal newborn hearing screening. *Mental Retardation and Developmental Disabilities Research Reviews* 2003;**9**:103–8.

224 Joubrel C, Gendron N, Dmytruk N, Touak G, Verlaguet M, Poyart C, *et al.* Comparative evaluation of 5 different selective media for Group B Streptococcus screening in pregnant women. *Diagnostic Microbiology and Infectious Disease* 2014;**80**:282–4.

225 Khurshid N, Connole S. To compare cost effectiveness for routine type and screen for patients undergoing cesarean section and vaginal delivery. *American Journal of Obstetrics and Gynecology* 2013;**208 (1 SUP**:S326–7.

226 Lee S, Lee E. Study on strategic approach of prenatal testing for trisomy 21 in general pregnancy population. *Clinical Chemistry* 2017;**63 (Supple**:S250.

227 Ong KJ, Soldan K, Jit M, Dunbar JK, Woodhall SC. Chlamydia sequelae cost estimates used in current economic evaluations: does one-size-fit-all? *Sexually Transmitted Infections* 2017;**93**:18–24.

228 Qutub M, Klapper P, Vallely P, Cleator G. Genital herpes in pregnancy: is screening cost-effective? *International Journal of STD and AIDS* 2001;**12**:14–6. https://doi.org/10.1258/0956462011916677.

229 Rey E, Hudon L, Michon N, Boucher P, Ethier J, Saint-Louis P. Fasting plasma glucose versus glucose challenge test: screening for gestational diabetes and cost effectiveness. *Clinical Biochemistry* 2004;**37**:780–4.

230 Rosignoli L, Tonni G. Should cell-free fetal DNA be included in first trimester screening (FTS) for common trisomy? A possible scenario on 6697 women screened over 10 years. *Journal of Evaluation in Clinical Practice* 2016;**22**:899–906.

231 Rosselli D, Rueda JD, Ruiz-Patino A. Cost analysis of universal neonatal screening for inborn errors of metabolism with tandem mass spectrometry in Colombia. *Pediatria* 2014;**47**:68–73.

232 Stillwaggon E, Perez-Zetune V, Bialek SR, Montgomery SP. Congenital Chagas Disease in the United States: Cost Savings through Maternal Screening. *American Journal of Tropical Medicine and Hygiene* 2018;**98**:1733–42.

233 Teljeur C, O’Neill M, Harrington P. Some issues with the icer in technology assessments of screening interventions for rare conditions. *Value in Health* 2012;**15 (7)**:A280.

234 Thaler M, Biedermann R, Lair J, Krismer M, Landauer F. Cost-effectiveness of universal ultrasound screening compared with clinical examination alone in the diagnosis and treatment of neonatal hip dysplasia in Austria. *Journal of Bone and Joint Surgery* 2011;**93**:1126–30.

235 van Heyningen AM, Levenston MJ, Tamminga N, Scoop-Martijn EG, Wever RM, Verhagen AA, *et al.* Estimated incidence of sickle-cell disease in Aruba and St. Maarten suggests cost-effectiveness of a universal screening programme for St. Maarten. *West Indian Medical Journal* 2009;**58**:301–4.

236 Wei D, Sardesai SR, Barton L. The c in torch: A cost-effective alternative to screening small-for-gestational-age infants. *Neonatology* 2014;**106**:24–9.

237 Seedat F, Taylor-Phillips S, Geppert J, Stinton C, Patterson J, Brown C, *et al.* *Universal antenatal culture-based screening for maternal Group B Streptococcus (GBS) carriage to prevent early-onset GBS disease*. 2016. URL: https://legacyscreening.phe.org.uk/policydb_download.php?doc=654%0A (Accessed February 17, 2021).

238 UK National Screening Committee. *First trimester combined screening for trisomy 13 and trisomy 18*. 2013. URL: https://legacyscreening.phe.org.uk/policydb_download.php?doc=451%0A (Accessed February 17, 2021).

239 UK National Screening Committee. *Newborn Screening for Duchenne Muscular Dystrophy*. 2011. URL: https://legacyscreening.phe.org.uk/policydb_download.php?doc=1028 (Accessed February 17, 2021).

240 Hawkes S, Gomez G. *Screening for syphilis in pregnancy*. 2013. URL: https://legacyscreening.phe.org.uk/policydb_download.php?doc=1183 (Accessed February 17, 2021).

241 UK National Screening Committee. *Screening for Preterm Labour in asymptomatic, low risk women*. 2014. URL: https://legacyscreening.phe.org.uk/policydb_download.php?doc=992 (Accessed February 17, 2021).

242 UK National Screening Committee. *Newborn Screening for Duchenne Muscular Dystrophy*. 2016. URL: https://legacyscreening.phe.org.uk/policydb_download.php?doc=1029 (Accessed February 17, 2021).

243 Public Health England. *Newborn Pulse Oximetry Screening Pilot*. 2016. URL: https://legacyscreening.phe.org.uk/documents/pulse-oximetry/NPOSP End Project Report.pdf (Accessed February 17, 2021).

244 Medical Services Advisory Committee. *Application No. 1492 – Non-invasive prenatal testing (NIPT) for trisomies 21, 18 and 13*. 2019. URL: https://www1.health.gov.au/internet/msac/publishing.nsf/Content/B1BCB2807D09C12DCA258258000F2025/$File/1492 Final PSD.pdf (Accessed February 17, 2021).

245 Morona J, Newton S, Wang S, Tamblyn D, Ellery B, Merlin T. *Genetic testing for hereditary mutations in the VHL gene that cause von Hippel-Lindau syndrome*. 2012. URL: https://www1.health.gov.au/internet/msac/publishing.nsf/Content/3474C4A5A7E262E5CA25801000123B1A/$File/Final Report for VHL 31-7-2012 accessible2015.pdf (Accessed February 17, 2021).

246 Federaal Kenniscentrum voor de Gezondheidszorg. *Is Neonatale Screening op Mucoviscidose aangewezen in België?*. 2010. URL: https://kce.fgov.be/sites/default/files/atoms/files/kce_132a_neonatale_screening_op_mucoviscidose.pdf (Accessed February 17, 2021).

247 Canadian Task Force on Preventive Health Care. *Recommendations on Screening for asymptomatic bacteriuria in pregnancy*. 2018. URL: https://canadiantaskforce.ca/wp-content/uploads/2018/06/KTStakeholderDeck-Script-2018-05-11.pdf (Accessed February 17, 2021).

248 Canadian Task Force on Preventive Health Care. *Dépistage de la bactériurie asymptomatique pendant la grossesse*. n.d. URL: https://canadiantaskforce.ca/wp-content/uploads/2018/06/ASBPregnancy_KTdeck_Fr.pdf (Accessed February 17, 2021).

249 Wingert A, Pillay J, Featherstone R, Gates M, Sebastianski M, Shave K, *et al.* *Screening for Asymptomatic Bacteriuria in Pregnancy: Systematic Review & Meta-analysis*. 2017. URL: https://canadiantaskforce.ca/wp-content/uploads/2019/03/Screening-for-Asymptomatic-Bacteriuria-in-Pregnancy-Final-Report-Appendices-13Oct2017.pdf (Accessed February 17, 2021).

250 Makni H, St-Hilaire C, Robb L, Larouche K, Blancquaert I. *Tandem Mass Spectrometry and Neonatal Blood Screening in Quebec*. 2007. URL: https://www.inesss.qc.ca/fileadmin/doc/AETMIS/Rapports/DepistageGenetique/2007_03_en.pdf (Accessed February 17, 2021).

251 Fröschl B, Brunner-Ziegler S, Wirl C. *Prävention des fetalen Alkoholsyndroms*. 2013. URL: https://portal.dimdi.de/de/hta/hta_berichte/hta330_bericht_de.pdf (Accessed February 17, 2021).

252 Gemeinsamer Bundesausschuss. *zum Beschluss des Gemeinsamen Bundesaus-schusses über eine Änderung der Gesundheitsuntersuchungs-Richtlinie (GU-RL): Einführung eines Screenings auf Hepatitis-B- und auf Hepatitis-C-Virusinfektion*. 2020. URL: https://www.g-ba.de/downloads/40-268-7078/2020-11-20_GU-RL_Screening-Hepatitis-B-und-C_TrG.pdf (Accessed February 17, 2021).

253 Glass N, Nelson H, Villemyer K. *Screening for Gonorrhea: Update of the Evidence*. 2005. URL: https://uspreventiveservicestaskforce.org/home/getfilebytoken/Wezro6zTSfAWxCrneF-nxB (Accessed February 17, 2021).

254 Statens beredning för medicinsk utvärdering. *Analys av foster-DNA i kvinnans blod: icke-invasiv fosterdiagnostik (NIPT) för trisomi 13, 18 och 21*. 2015. URL: https://www.sbu.se/contentassets/0dcc05c3977b45bcb598a20909f2d07b/analys_foster-dna_kvinnans_blod_icke-invasiv_fosterdiagnostik_nipt_201503.pdf (Accessed February 17, 2021).

255 National Institute for Health and Care Excellence. *Twin and Triplet Pregnancy: [A] Evidence review for ultrasound screening for feto-fetal transfusion syndrome*. 2019. URL: https://www.nice.org.uk/guidance/ng137/evidence/a-ultrasound-screening-for-fetofetal-transfusion-syndrome-pdf-6898605373 (Accessed February 17, 2021).

256 Unterausschuss Methodenbewertung. *SGB V auf Bewertung eines Neugeborenenscreenings auf Spinale*. 2019. URL: https://www.g-ba.de/downloads/40-268-5424/2019-11-22_Einleitung-Beratungsverfahren-Screening-spinale-Muskelatrophie_Antrag-PatV.pdf (Accessed February 17, 2021).

257 Albright C, Werner E, Anderson B. Universal cytomegalovirus screening in pregnancy: A cost-effectiveness analysis. *American Journal of Obstetrics and Gynecology* 2015;**212**:S307–8.

258 Berruti A, Gift T. Cost and cost-effectiveness of antenatal screening for congenital syphilis in the U.S. *Sexually Transmitted Diseases* 2018;**45 (Supple**:S70.

259 Bert F, Gualano MR, Biancone P, Brescia V, Camussi E, Martorana M, *et al.* HIV-screening in pregnant women: a systematic review of cost-effectiveness studies. *European Journal of Public Health* 2016;**26**:341.

260 Bessey A, Chilcott J, Pandor A, Paisley S. The Cost-Effectiveness of Expanding the Nhs Newborn Bloodspot Screening Programme To Include Homocystinuria (Hcu), Maple Syrup Urine Disease (Msud), Glutaric Aciduria Type 1 (Ga1), Isovaleric Acidaemia (Iva), and Long-Chain Hydroxyacyl-Coa Dehydrogenase D. *Value in Health* 2014;**17**:A531.

261 Bessey A, Leaviss J, Galvan De la Cruz C, Chilcott J, Wong R. The cost-effectiveness of screening for severe combined immunodeficiency (SCID) in the UK NHS newborn bloodspot screening programme. *Value in Health* 2017;**20 (9)**:A501.

262 Bessos H, Turner M, Fagge T, Harkness M, Rentoul F, Seymour J, *et al.* Health economic evaluation of the cost effectiveness of antenatal screening for HPA-1a induced neonatal alloimmune thrombocytopenia (NAIT). *Transfusion* 2003;**43**:38A-38A.

263 Beulen L, Grutters JPC, Bekker MN, Van Vugt JMG. The implementation of noninvasive prenatal diagnosis in national health care: A decision-analytic economic model. *Prenatal Diagnosis* 2013;**33**:69.

264 Caughey A, Norton M, Kuppermann M, Washington AE. First vs. second trimester screening tools for Down syndrome: A cost-utility analysis. *American Journal of Obstetrics and Gynecology* 2001;**185**:S224–S224. https://doi.org/10.1016/s0002-9378(01)80558-5.

265 Chaillon A, Reau N, Rand E, Martin N. The Cost-Effectiveness of HCV Screening of Pregnant Women in the United States. *Hepatology* 2018;**68**:573A-574A.

266 Chilcott J, Bessey A, Leaviss J, Sutton A. Potential cost-effectiveness of including screening for x-linked adrenoleukodystrophy in the UK national health service newborn blood spot screening program. *Journal of Inborn Errors of Metabolism and Screening* 2017;**5**:43–4.

267 Chowers M, Shavit O. Economic evaluation of universal antenatal HIV screening compared with current “at risk” policy in Israel. *Value in Health* 2013;**16 (7)**:A359.

268 Cipriano LE, Rupar CA, Zaric GS. The cost-effectiveness of expanding newborn screening for inherited metabolic disorders using tandem mass spectrometry. *Value in Health* 2005;**8**:298. https://doi.org/10.1016/s1098-3015(10)62762-3.

269 Donnay S, Balsa JA, Alvarez J, Crespo C, Perez-Alcantara F, Villacampa A, *et al.* Cost-effectiveness of universal screening for thyroid disease in pregnant women in Spain. *Value in Health* 2013;**16**:A438–A438. https://doi.org/10.1016/j.jval.2013.08.662.

270 Dosiou C, Barnes J, Schwartz A, Negro R, Crapo L, Stagnaro-Green A. Cost-effectiveness of universal and risk-based screening for autoimmune thyroid disease in pregnant women. *Journal of Clinical Endocrinology and Metabolism* 2012;**97**:1536–46.

271 Hamel M, Orzechowski K, Berghella V, Thung S, Werner E. Cost-effectiveness of transvaginal ultrasound cervical length screening in singletons without prior preterm birth: An update. *American Journal of Obstetrics and Gynecology* 2015;**212**:S90–1.

272 Hersh AR, Megli CJ, Caughey AB. Universal repeat screening for syphilis in the third trimester of pregnancy: a cost-effective analysis. *American Journal of Obstetrics and Gynecology* 2017;**216**:S183–S183. https://doi.org/10.1016/j.ajog.2016.11.556.

273 Hillman SC, Barton PM, Roberts TE, Maher ER, Kilby MD. Chromosomal microarray (CMA) use for the prenatal detection of chromosome anomalies: Model-based health economic evaluation. *Archives of Disease in Childhood: Fetal and Neonatal Edition* 2013;**98**:.

274 Hopkins M, Dugoff L, Durnwald C, Havrilesky L, Dotters-Katz S. Cell-free DNA for Down syndrome screening in morbidly obese women: Is it a cost-effective strategy? *American Journal of Obstetrics and Gynecology* 2019;**220 (1 Sup**:S583–4.

275 Ibekwe E, Francis Fatoye F, Haigh C. Economic impact of routine opt-out antenatal HIV screening: A systematic review. *Value in Health* 2017;**20 (5)**:A353.

276 Keren R, Helfand M, Homer C, McPhillips H, Lieu TA. Projected cost-effectiveness of statewide universal newborn hearing screening. *Pediatrics* 2002;**110**:855–64.

277 Killie MK, Kjeldsen-Kragh J, Husebekk A, Skogen B, Olsen JA, Kristiansen IS. Cost-utility analysis of antenatal screening for neonatal alloimmune thrombocytopenia. *Vox Sanguinis* 2006;**91**:263.

278 Little SE, Janakiraman V, Kaimal A, Musci T, Ecker J, Caughey AB. The cost-effectiveness of prenatal screening for spinal muscular atrophy. *American Journal of Obstetrics and Gynecology* 2010;**202**:253.e1-7.

279 Londono D, Taborda A, Dominguez MT, Sandoval NS, Troncoso GT, Fonseca AF, *et al.* Cost-effectiveness analysis of neonatal screening with pulse oximetry for the detection of critical congenital heart disease in Colombia, 2017. *Value in Health* 2017;**20 (9)**:A587.

280 Mahlaoui N, Clement M, Mignot C, Le Bihan C, Rabetrano H, Hoang LY, *et al.* Systematic neonatal screening for severe combined immunodeficiency and severe T-cell lymphopenia: Analysis of cost-effectiveness based on french real field data. *Journal of Clinical Immunology* 2014;**34**:S162.

281 Masucci L, Bryan S, Kaczorowski J, Collet JP, Schreiber RA. Cost-effectiveness analysis of universal newborn screening strategies for biliary atresia. *Gastroenterology* 2017;**152 (5 Sup**:S1157.

282 Masucci L, Bryan S, Kaczorowski JA, Collet JP, Schreiber RA. The cost-effectiveness of universal screening for biliary atresia in Canada. *Hepatology* 2011;**54**:595A-596A.

283 Masucci L, Schreiber RA, Kaczorowski J, Collet JP, Bryan S. Universal screening of newborns for biliary atresia: Cost-effectiveness of alternative strategies. *Journal of Medical Screening* 2019;**26**:113–9.

284 McGhee SA, McCabe ERB, Stiehm ER. Cost-effectiveness of newborn screening for severe combined immunodeficiency. *Journal of Investigative Medicine* 2004;**52**:S127–S127. https://doi.org/10.1097/00042871-200401001-00274.

285 Mission J, Ohno M, Yanit K, Cheng Y, Caughey A. Gestational diabetes screening with the new IADPSG 2 hour glucose tolerance test vs the 1 hour glucose challenge test: A cost-effectiveness analysis. *American Journal of Obstetrics and Gynecology* 2012;**206**:S126.

286 Mogul D, Zhou M, Intihar P, Schwarz KB, Frick K. Cost-effective analysis of screening for biliary atresia with the stool color card. *Hepatology* 2013;**58**:216A.

287 Mone F, Mulcahy C, McParland P, O’Mahony J, Tyrell E, Breathnach F, *et al.* A randomised controlled trial and cost-effectiveness analysis of low dose aspirin with an early screening test for preeclampsia in low risk women. *Reproductive Sciences* 2017;**24 (1 Supp**:68A.

288 Mukerji A, Shafey A, Jain A, Cohen E, Shah P, Shah V, *et al.* Cost-effectiveness of pulse oximetry screening for critical congenital heart defects in Ontario. *Paediatrics & Child Health* 2018;**23 (Supple**:e17–8.

289 Nshimyumukiza L, Bois A, Daigneault P, Lands L, Laberge A, Fournier D, *et al.* Simulation of cost-effectiveness of newborn screening for cystic fibrosis in the province of Quebec (Canada). *Pediatric Pulmonology* 2013;**48**:381.

290 O’Mahony JF, Mone F, Tyrrell E, Mulcahy C, McParland P, Breathnach F, *et al.* The cost effectiveness of a policy of universal aspirin versus aspirin indicated by a positive pre-eclampsia screening test. *American Journal of Obstetrics and Gynecology* 2017;**216 (1 Sup**:S483.

291 Peterson C, Grosse SD, Cassell CH, Oster ME, Olney RS. A cost-effectiveness analysis of universal pulse oximetry screening to detect critical congenital heart disease in U.S. Newborns. *Circulation* 2012;**5**:.

292 Pfeil J, Listl S, Hoffmann GF, Kolker S, Lindner M, Burgard P. Newborn screening by tandem mass spectrometry for glutaric aciduria type 1: A cost-effectiveness analysis. *Molecular Genetics and Metabolism* 2014;**111 (3)**:299.

293 Pinto N, Nelson R, Smith K, Metz TD, Puchalski M. Cost-effectiveness of prenatal screening strategies for congenital heart disease (CHD). *Journal of the American Society of Echocardiography* 2012;**25 (6)**:B27.

294 Rours GIJG, Verkooijen RP, Verbrugh HA, Postma MJ. Cost-effectiveness of screening for chlamydia trachomatis in dutch pregnant women. *Sexually Transmitted Infections* 2011;**87**:A61–2.

295 Schwartz PJ, Quaglini S, Rognoni C, Spazzolini C, Mannarino S, Priori SG. Cost-effectiveness of neonatal ECG screening the long QT syndrome. *European Heart Journal* 2005;**26**:552.

296 Sicuri E, Munoz J, Pinazo MJ, Posada E, Sanchez J, Alonso P, *et al.* Economic evaluation of Chagas disease screening of pregnant Latin American women and of their infants in a non-endemic area. *Tropical Medicine and International Health* 2009;**14**:241.

297 Sinkey R, Odibo A. Screening strategies for vasa previa during the mid-trimester ultrasound: A decision and cost-effective analysis. *American Journal of Obstetrics and Gynecology* 2016;**214**:S257.

298 Thung SF, Grobman WA. The cost-effectiveness of routine antenatal screening for maternal herpes simplex virus 1 and 2 antibodies. *American Journal of Obstetrics and Gynecology* 2003;**189**:S97–S97. https://doi.org/10.1016/j.ajog.2003.10.115.

299 Urbanus A, van Keep M, Matser A, Rozenbaum M, Weegink C, van den Hoek A, *et al.* Is adding HCV screening to the antenatal national screening program in Amsterdam, The Netherlands cost-effective? *Journal of Hepatology* 2013;**58**:S22–S22. https://doi.org/10.1016/s0168-8278(13)60052-5.

300 Van Der Ploeg CP, Vernooij-van Langen AM, Van Den Akker-Van Marle ME, Elvers B, Gille H, Dankert-Roelse JE. Cost-effectiveness of neonatal screening for cystic fibrosis. *Pediatric Pulmonology* 2010;**45**:394.

301 Venditti CP, Venditti LN, Kaplan PB, Kaye EM, Glick H, Stanley CA, *et al.* Newborn screening by tandem mass spectrometry for medium chain Acyl-CoA dehydrogenase deficiency: Is it cost effective? *Pediatric Research* 2002;**51**:225A-226A.

302 Venditti CP, Venditti LN, Stanley CA, Berry GT, Kaplan P, Kaye EM, *et al.* Newborn screening by tandem mass spectrometry for medium chain acyl-CoA dehydrogenase deficiency: Is it cost effective? *American Journal of Human Genetics* 2001;**69**:444.

303 Waites BT, Walker AR, Skeith AE, Caughey AB. First trimester fasting plasma glucose screen in advanced maternal age to detect pre-existing glucose intolerance. *Obstetrics and Gynecology* 2018;**131 (Suppl**:168S.

304 Walker AR, Valent A, Caughey AB. Positivity thresholds of HbA1c assay as a screening test for gestational diabetes mellitus in the first trimester in high-risk populations. *American Journal of Obstetrics and Gynecology* 2017;**216 (1 Sup**:S291–2.

305 Wastlund D, Moraitis A, Dacey A, Sovio U, Wilson E, Smith G. Screening for breech presentation using late pregnancy ultrasonography: a prospective cohort study and cost-effectiveness analysis. *BJOG: An International Journal of Obstetrics & Gynaecology* 2019;**126**:125.

306 Wastlund D, Moraitis A, Thornton J, Sanders J, White I, Brocklehurst P, *et al.* The cost-effectiveness of universal late pregnancy ultrasound screening for fetal macrosomia in low-risk women. *BJOG: An International Journal of Obstetrics & Gynaecology* 2019;**126**:123.

307 Werner E, Han C, Pettker C, Buhimschi C, Copel J, Funai E, *et al.* Universal cervical length screening to prevent preterm birth: A cost-effectiveness analysis. *American Journal of Obstetrics and Gynecology* 2009;**201**:S224–5.

308 Werner EF, Pettker CM, Reel M, Zuckerwise LC, Funai EF, Thung SF. Long term diabetes risk reduction necessary for gestational diabetes screening to be cost effective. *American Journal of Obstetrics and Gynecology* 2012;**206**:S122–3.

309 Zantow E, Williams M, Turrentine M. Evaluating the cost effectiveness of the latest recommendations for Group B Streptococcus screening. *American Journal of Obstetrics and Gynecology* 2020;**222 (1 Sup**:S488.

310 Zhang W, Mohammadi T, Sou J, Anis A. Pns52 Can Cost-Effectiveness Analysis Inform the Extent of Coverage under Public Healthcare? A Microsimulation Model of Alternative Prenatal Screening and Diagnostic Strategies. *Value in Health* 2019;**22 (Supple**:S295.

311 Adams EJ, Turner KM, Edmunds WJ. The cost effectiveness of opportunistic chlamydia screening in England. *Sexually Transmitted Infections* 2007;**83**:267–74.

312 Allan WC, Timothy K, Vincent GM, Palomaki GE, Neveux LM, Haddow JE. Long QT syndrome in children: the value of rate corrected QT interval and DNA analysis as screening tests in the general population. *Journal of Medical Screening* 2001;**8**:173–7.

313 Allen A, Shaffer BL, Caughey AB, Pilliod RA. Nuchal translucency ultrasound in women with low risk cell free DNA screening: A cost-effectiveness analysis. *American Journal of Obstetrics and Gynecology* 2020;**222 (1 Sup**:S517.

314 Askew A, Heine RP, Myers E, Swamy G. Cost-effectivenss of penicillin skin testing in GBS plus pregnant women with penicillin allergy. *American Journal of Obstetrics and Gynecology* 2015;**212**:S300–S300. https://doi.org/10.1016/j.ajog.2014.10.809.

315 Barbosa C, Smith E, Hoerger T, Fenlon N, Schillie S, Bradley C, *et al.* Cost-effectiveness analysis of the national Perinatal Hepatitis B Prevention Program. *Pediatrics* 2014;**133**:243–53.

316 Beauchamp KA, Johansen Taber KA, Muzzey D. Clinical impact and cost-effectiveness of a 176-condition expanded carrier screen. *Genetics in Medicine* 2019;**21**:1948–57.

317 Bernstein KT, Mehta SD, Rompalo AM, Erbelding EJ. Cost-effectiveness of screening strategies for gonorrhea among females in private sector care. *Obstetrics and Gynecology* 2006;**107**:813-821\.

318 Desplanches T, Lejeune C, Cottenet J, Sagot P, Quantin C. Cost-effectiveness of diagnostic tests for threatened preterm labor in singleton pregnancy in France. *Cost Effectiveness and Resource Allocation* 2018;**16**:21.

319 Gray A, Elbourne D, Dezateux C, King A, Quinn A, Gardner F. Economic evaluation of ultrasonography in the diagnosis and management of developmental hip dysplasia in the United Kingdom and Ireland. *Journal of Bone and Joint Surgery* 2005;**87**:2472–9.

320 Greeley SA, John PM, Winn AN, Ornelas J, Lipton RB, Philipson LH, *et al.* The cost-effectiveness of personalized genetic medicine: the case of genetic testing in neonatal diabetes. *Diabetes Care* 2011;**34**:622-627\.

321 Grimshaw GM, Szczepura A, Hulten M, MacDonald F, Nevin NC, Sutton F, *et al.* Evaluation of molecular tests for prenatal diagnosis of chromosome abnormalities. *Health Technology Assessment* 2003;**7**:1–146.

322 Harris RA, Washington AE, Nease Jr. RF, Kuppermann M. Cost utility of prenatal diagnosis and the risk-based threshold. *The Lancet* 2004;**363**:276–82.

323 Jacklin PB, Maresh MJ, Patterson CC, Stanley KP, Dornhorst A, Burman-Roy S, *et al.* A cost-effectiveness comparison of the NICE 2015 and WHO 2013 diagnostic criteria for women with gestational diabetes with and without risk factors. *BMJ Open* 2017;**7**:e016621.

324 Jackson KM, Scott KE, Graff Zivin J, Bateman DA, Flynn JT, Keenan JD, *et al.* Cost-utility analysis of telemedicine and ophthalmoscopy for retinopathy of prematurity management. *Archives of Ophthalmology* 2008;**126**:493-499\.

325 MacDonell-Yilmaz R, Anderson K, DeNardo B, Sprinz P, Padula W V. Cost-effectiveness Analysis of Screening Extremely Low Birth Weight Children for Hepatoblastoma Using Serum Alpha-fetoprotein. *Journal of Pediatrics* 2020;**225**:80-89 e4. https://doi.org/10.1016/j.jpeds.2020.05.041.

326 Porter HL, Neely ST, Gorga MP. Using benefit-cost ratio to select Universal Newborn Hearing Screening test criteria. *Ear and Hearing* 2009;**30**:447–57.

327 Toledano-Alhadef H, Basel-Vanagaite L, Magal N, Davidov B, Ehrlich S, Drasinover V, *et al.* Fragile-X carrier screening and the prevalence of premutation and full-mutation carriers in Israel. *American Journal of Human Genetics* 2001;**69**:351–60.

328 Ulph F, Wright S, Dharni N, Payne K, Bennett R, Roberts S, *et al.* Provision of information about newborn screening antenatally: a sequential exploratory mixed-methods project. *Health Technology Assessment* 2017;**21**:1–240.

329 Liufu V, Mundy L, Hiller J. *Screening for gestational diabetes*. 2008. URL: https://www1.health.gov.au/internet/horizon/publishing.nsf/Content/BB580B674729F620CA2575AD0080F351/$File/Volume_21_Update_Aug_2008_Gestational_Diabetes.pdf (Accessed February 17, 2021).

330 Medical Services Advisory Committee. *Application No. 1531 – Alpha Thalassaemia genetic testing*. 2019. URL: https://www1.health.gov.au/internet/msac/publishing.nsf/Content/BDB27F8F2CD7839DCA2583B7000013BA/$File/1531 Final PSD-Mar2019.pdf (Accessed February 17, 2021).

331 Myrhaug H, Reinar L, Stoinska-Schneider A, Hval G, Movik E, Brurberg K, *et al.* *Safety, clinical effectiveness, predictive accuracy and cost effectiveness of blood based tests for women with suspected preeclampsia*. 2020. URL: https://www.fhi.no/contentassets/902cc5b12aa94c01a8199a28239b052d/safety-clinical-effectiveness-predictive-accuracy-and-cost-effectiveness-of-blood-based-tests-for-women-with-suspected-preeclampsia-hta-2020.pdf (Accessed February 17, 2021).

332 National Institute for Public Health and the Environment. *Chlamydia Screening Implementation Netherlands: Impact evaluation and cost-effectiveness*. 2010. URL: https://www.rivm.nl/bibliotheek/rapporten/210261008.pdf (Accessed February 17, 2021).

333 Lewis, D; Barham L. *Economic Modelling of Interventions to Reduce the Transmission of Chlamydia and other Sexually Transmitted Infections and to Reduce the Rate of Under Eighteen Conceptions*. 2006. URL: https://www.nice.org.uk/guidance/ph3/evidence/economic-modelling-report-pdf-124482061 (Accessed February 17, 2021).

334 Adeniji AA, Fuller I, Dale T, Lindow SW. Should we continue screening rhesus D positive women for the development of atypical antibodies in late pregnancy? *Journal of Maternal-Fetal and Neonatal Medicine* 2007;**20**:59–61.

335 Barre S, Corbillon E. Economic evaluation of strategies for screening newborns for bilateral hearing impairment in France. *Value in Health* 2006;**9**:A139–40. https://doi.org/10.1016/s1098-3015(10)64737-7.

336 Dangouloff T, Hiligsmann M, Caberg J, Boemer F, Servais L. Development of a decision-analytic model for the economic evaluation of newborn screening for spinal muscular atrophy. *Neuromuscular Disorders* 2018;**28**:S59–S59. https://doi.org/10.1016/j.nmd.2018.06.125.

337 Dangouloff T, Servais L, Hiligsmann M. SMA: Registries, Biomarkers & Outcome Measures. *Neuromuscular Disorders* 2020;**30**:. https://doi.org/10.1016/j.nmd.2020.08.193.

338 Finegold DN, Naylor EW, Chace DH, Kamlet M. Cost effectiveness of tandem mass spectrometry (MS/MS) for neonatal screening: Medium chain acyl-CoA dehydrogenase (MCAD) as a model. *American Journal of Human Genetics* 2001;**69**:443.

339 Kingston D, Kingston D, McDonald S, Biringer A, Austin MP, McDonald SD, *et al.* Comparing the Acceptability, Clinical-, and Cost-effectiveness of Mental Health E-screening to Paper-based Screening in Pregnant Women: a Randomized, Parallel-group, Superiority Trial. *European Psychiatry* 2015;**30**:. https://doi.org/10.1016/s0924-9338(15)30773-2.

340 Komakech H, Muhumuza C, Lamorde M, Marques E, Kuznik A. The cost-effectiveness of antenatal syphilis screening using point-of-care testing in Latin America. *Value in Health* 2014;**17 (3)**:A160.

341 Korres S, Nikolopoulos TP, Komkotou V, Balatsouras D, Kandiloros D, Constantinou D, *et al.* Newborn hearing screening: effectiveness, importance of high-risk factors, and characteristics of infants in the neonatal intensive care unit and well-baby nursery. *Otology and Neurotology* 2005;**26**:1186–90.

342 Munster JM, Leenders A, Van der Hoek W, Schneeberger PM, Rietveld A, Riphagen-Dalhuisen J, *et al.* (Cost-) Effectiveness of a Screening Strategy for Q Fever among Pregnant Women in Risk Areas: A Clustered Randomized Controlled Trial. *Pharmacoepidemiology and Drug Safety* 2011;**20**:S50–1.

343 Thomas C, Mirallie S, Durand-Zaleski I, Sebille V, Mahlaoui N, Fischer A, *et al.* Clinical and cost-effectiveness prospective study of neonatal screening for severe combined immunodeficiency using the t-cell receptor excision circles assay in a french multicentre study. *Journal of Clinical Immunology* 2014;**34**:S377.

344 Anonymous. Noninvasive prenatal testing for trisomies 21, 18, and 13, sex chromosome aneuploidies, and microdeletions: A health technology assessment. *Ontario Health Technology Assessment Series* 2019;**19**:1–166.

345 Kanga I, Williams D, Hatchette T, MacKinnon SB, Jung H, Black C, *et al.* Screening for Chlamydia Trachomatis and Neisseria Gonorrhoeae During Pregnancy: A Health Technology Assessment. *Canadian Agency for Drugs and Technologies in Health CADTH Health Technology Assessments* 2018;**11**:11.

346 Konomura K, Tamura Y, Akazawa M, Fukuda T. Cost-Utility Analysis of Newborn Screening Program by Tandem Mass Spectrometry in Japan. *Value in Health* 2019;**22 (Supple**:S632.

347 Krantz DA, Hallahan TW, Carmichael JB, Liu HP. First trimester screening for early onset preeclampsia is a cost effective approach in prenatal care. *Pregnancy Hypertension* 2015;**5 (1)**:92.

348 Ramos Gon J, Serrano Aguilar P, Saenz-Torres M, Posada M. Cost-effectiveness of neonatal screening for congenital errors of metabolism using tandem mass spectrometry. *Value in Health* 2009;**12 (3)**:A164.

349 Torres YAM, Rojas JAD, Garay OAG. Cost effectiveness of universal neonatal hearing screening with otoacoustic emissions and/or automated auditory brainstem response, for the detection of bilateral congenital hearing loss and early treatment, in newborns without risk factors, in Colombia. *Value in Health* 2017;**20**:A586–7.

350 Wetzel S, Miller ES, Cirino N, Dukhovny D, Ameel B, Caughey AB. Routine antenatal screening for depression: what are the outcomes and is it cost-effective? *American Journal of Obstetrics and Gynecology* 2016;**214**:S383–4. https://doi.org/10.1016/j.ajog.2015.10.779.

351 Beulen L, Grutters JPC, Faas BHW, Feenstra I, Groenewoud H, van Vugt JMG, *et al.* Women’s and healthcare professionals’ preferences for prenatal testing: A discrete choice experiment. *Prenatal Diagnosis* 2015;**35**:549–57. https://doi.org/10.1002/pd.4571.

352 Carroll F, Al‐Janabi H, Flynn T, Montgomery A. Women and their partners’ preferences for Down’s syndrome screening tests: a discrete choice experiment. *Prenatal Diagnosis* 2013;**33**:449–56.

353 Rouse DJ, Stringer JS. An appraisal of screening for maternal type-specific herpes simplex virus antibodies to prevent neonatal herpes. *American Journal of Obstetrics and Gynecology* 2000;**183**:400–6. https://doi.org/10.1067/mob.2000.105967.

354 Vintzileos AM, Ananth C V, Smulian JC, Beazoglou T, Knuppel RA. Routine second-trimester ultrasonography in the United States: a cost-benefit analysis. *American Journal of Obstetrics and Gynecology* 2000;**182**:655–60.

355 Wald NJ, Rodeck C, Hackshaw AK, Walters J, Chitty L, Mackinson AM. First and second trimester antenatal screening for Down’s syndrome: The results of the serum, urine and ultrasound screening study (SURUSS). *Health Technology Assessment* 2003;**7**:.

356 Walker B, Jackson B, LaGrave D, Ashwood E, Schmidt R. A cost-effectiveness analysis of cell free DNA as a replacement for serum screening for Down syndrome. *Prenatal Diagnosis* 2015;**35**:440–6.

357 Berrigan P, Andrew G, Reynolds JN, Zwicker JD. The cost-effectiveness of screening tools used in the diagnosis of fetal alcohol spectrum disorder: a modelled analysis. *BMC Public Health* 2019;**19**:. https://doi.org/10.1186/s12889-019-8110-5.

358 Abbey R, Dunsmoor-Su R. Cost-benefit analysis of indirect antiglobulin screening in Rh(D)-negative women at 28 weeks of gestation. *Obstetrics and Gynecology* 2014;**123**:938–45.

359 Albright CM, Emerson JB, Werner EF, Hughes BL. Third-Trimester Prenatal Syphilis Screening: A Cost-Effectiveness Analysis. *Obstetrics and Gynecology* 2015;**126**:479–85.

360 Zaric GS, Bayoumi AM, Brandeau ML, Owens DK. The cost effectiveness of voluntary prenatal and routine newborn HIV screening in the United States. *JAIDS: Journal of Acquired Immune Deficiency Syndromes* 2000;**25**:403–16.

361 Castilla-Rodriguez I, Cela E, Vallejo-Torres L, Valcarcel-Nazco C, Dulin E, Espada M, *et al.* Cost-effectiveness analysis of newborn screening for sickle-cell disease in Spain. *Expert Opinion on Orphan Drugs* 2016;**4**:567–75.

362 Daniels J, Gray J, Pattison H, Roberts T, Edwards E, Milner P, *et al.* Rapid testing for group B streptococcus during labour: a test accuracy study with evaluation of acceptability and cost-effectiveness. *Health Technology Assessment* 2009;**13**:1–154, iii.

363 Doyle NM, Levison JE, Gardner MO. Rapid HIV versus enzyme-linked immunosorbent assay screening in a low-risk Mexican American population presenting in labor: a cost-effectiveness analysis. *American Journal of Obstetrics and Gynecology* 2005;**193**:1280–5.

364 Duplantie J, Gonzales OM, Bois A, Nshimyumukiza L, Gekas J, Bujold E, *et al.* Cost-effectiveness of the management of rh-negative pregnant women. *Journal of Obstetrics and Gynaecology Canada* 2013;**35**:730–40.

365 Gomez M. A comparison of three screening strategies for prevention of perinatal HIV infection in Colombia: a decision analysis model. *Pan American Journal of Public Health* 2008;**24**:256–64.

366 Immergluck LC, Cull WL, Schwartz A, Elstein AS. Cost-effectiveness of universal compared with voluntary screening for human immunodeficiency virus among pregnant women in Chicago. *Pediatrics* 2000;**105**:E54.

367 Mol B, Kamphuis E, Naber S, Habbema D, De Groot C. The impact of cervical screening on preterm birth: A decision analysis. *American Journal of Obstetrics and Gynecology* 2016;**214**:S351.

368 Ortved D, Hawkins TL, Johnson JA, Hyett J, Metcalfe A. Cost-effectiveness of first-trimester screening with early preventative use of aspirin in women at high risk of early-onset pre-eclampsia. *Ultrasound in Obstetrics and Gynecology* 2019;**53**:239–44.

369 Pinot De Moira A, Edmunds WJ, Breuer J. The cost-effectiveness of antenatal varicella screening with post-partum vaccination of susceptibles. *Vaccine* 2006;**24**:1298-1307\.

370 Poncet B, Touzet S, Rocher L, Berland M, Orgiazzi J, Colin C. Cost-effectiveness analysis of gestational diabetes mellitus screening in France. *European Journal of Obstetrics, Gynecology, & Reproductive Biology* 2002;**103**:122–9.

371 Rivera-Alsina ME, Rivera CC, Rollene N, Kirby RS, Ayres A, McNamara M. Voluntary screening program for HIV in pregnancy. Cost effectiveness. *Journal of Reproductive Medicine* 2001;**46**:243–8.

372 Stan CM, Boulvain M, Bovier PA, Auckenthaler R, Berner M, Irion O. Choosing a strategy to prevent neonatal early-onset group B streptococcal sepsis: economic evaluation. *BJOG: An International Journal of Obstetrics & Gynaecology* 2001;**108**:840–7.

373 Wastlund D, Moraitis AA, Dacey A, Sovio U, Wilson ECF, Smith GCS. Screening for breech presentation using universal late-pregnancy ultrasonography: A prospective cohort study and cost effectiveness analysis. *PLoS Medicine* 2019;**16**:. https://doi.org/10.1371/journal.pmed.1002778.

374 Williams M, Zantow E, Turrentine M. Cost Effectiveness of Latest Recommendations for Group B Streptococci Screening in the United States. *Obstetrics and Gynecology* 2020;**10**:10.

375 Sansom, S. Human immunodeficiency virus retesting during pregnancy: costs and effectiveness in preventing perinatal transmission. *Obstetrics and Gynecology* 2003;**102**:782–90. https://doi.org/10.1016/s0029-7844(03)00624-0.

376 Mrus JM, Tsevat J. Cost-effectiveness of interventions to reduce vertical HIV transmission from pregnant women who have not received prenatal care. *Medical Decision Making* 2004;**24**:30–9. https://doi.org/10.1177/0272989X03261570.

377 Park F, Deeming S, Bennett N, Hyett J. Cost effectiveness analysis of a model of first trimester prediction and prevention for preterm preeclampsia against usual care. *Ultrasound in Obstetrics and Gynecology* 2020. https://doi.org/10.1002/uog.22193.

378 Rodriguez PJ, Roberts DA, Meisner J, Sharma M, Owiredu MN, Gomez B, *et al.* Cost-effectiveness of dual maternal HIV and syphilis testing strategies in high and low HIV prevalence countries: a modelling study. *The Lancet Global Health* 2021;**9**:e61–71. https://doi.org/10.1016/s2214-109x(20)30395-8.

379 Arentz-Hansen H, Brurberg K, Kvamme M, Stoinska-Schneider A, Hofmann B, Ormstad S, *et al.* *Rhesus typing av foster basert på blodprøve fra rhesus negative gravide*. 2014. URL: https://www.fhi.no/globalassets/dokumenterfiler/rapporter/2014/rapport_2014_25_rhesustyping_foster.pdf%0A (Accessed February 20, 2021).

380 Huntington S, Weston G, Adams E. *Repeat screening for syphilis in pregnancy as an alternative screening strategy in the UK - a cost-effectiveness analysis*. 2020. URL: https://legacyscreening.phe.org.uk/policydb_download.php?doc=1306%0A (Accessed February 20, 2021).

381 van den Akker-van Marle ME, Dankert HM, Verkerk PH, Dankert-Roelse JE. Cost-effectiveness of 4 neonatal screening strategies for cystic fibrosis. *Pediatrics* 2006;**118**:896–905.

382 Benn P, Curnow KJ, Chapman S, Michalopoulos SN, Hornberger J, Rabinowitz M. An Economic Analysis of Cell-Free DNA Non-Invasive Prenatal Testing in the US General Pregnancy Population. *PLoS ONE* 2015;**10**:e0132313.

383 Beulen L, Grutters JP, Faas BH, Feenstra I, van Vugt JM, Bekker MN. The consequences of implementing non-invasive prenatal testing in Dutch national health care: a cost-effectiveness analysis. *European Journal of Obstetrics, Gynecology, and Reproductive Biology* 2014;**182**:53–61.

384 Biggio Jr. JR, Morris TC, Owen J, Stringer JS. An outcomes analysis of five prenatal screening strategies for trisomy 21 in women younger than 35 years. *American Journal of Obstetrics and Gynecology* 2004;**190**:721–9.

385 Bramley D, Graves N, Walker D. The cost effectiveness of universal antenatal screening for HIV in New Zealand. *AIDS* 2003;**17**:741–8.

386 Christiansen M, Olesen Larsen S. An increase in cost-effectiveness of first trimester maternal screening programmes for fetal chromosome anomalies is obtained by contingent testing. *Prenatal Diagnosis* 2002;**22**:482–6.

387 Chung EH, Lim SL, Havrilesky LJ, Steiner AZ, Dotters-Katz S. The cost-effectiveness of prenatal congenital heart defect screening methods in IVF pregnancies. *Ultrasound in Obstetrics and Gynecology* 2020;**18**:18.

388 Dhaifalah I, Majek O. Cost effectiveness, the economic considerations of prenatal screening strategies for trisomy 21 in the Czech Republic. *Ceska Gynekologie* 2012;**77**:39–51.

389 Dormandy E, Bryan S, Gulliford M, Roberts T, Ades A, Calnan M, *et al.* Antenatal screening for haemoglobinopathies in primary care: a cohort study and cluster randomised trial to inform a simulation model. The Screening for Haemoglobinopathies in First Trimester (SHIFT) trial. *Health Technology Assessment* 2010;**14**:1–60.

390 Gekas J, Durand A, Bujold E, Vallee M, Forest JC, Rousseau F, *et al.* Cost-effectiveness and accuracy of prenatal Down syndrome screening strategies: should the combined test continue to be widely used? *American Journal of Obstetrics and Gynecology* 2011;**204**:175.e1-8.

391 Gekas J, Gagne G, Bujold E, Douillard D, Forest JC, Reinharz D, *et al.* Comparison of different strategies in prenatal screening for Down’s syndrome: cost effectiveness analysis of computer simulation. *BMJ* 2009;**338**:b138.

392 Gilbert RE, Augood C, Gupta R, Ades AE, Logan S, Sculpher M, *et al.* Screening for Down’s syndrome: effects, safety, and cost effectiveness of first and second trimester strategies. *BMJ* 2001;**323**:423–5.

393 Graves N, Walker DG, McDonald AM, Kaldor JM, Ziegler JB. Would universal antenatal screening for HIV infection be cost-effective in a setting of very low prevalence? Modelling the data for Australia. *Journal of Infectious Diseases* 2004;**190**:166–74.

394 Hacker F, Griffin E, Shaffer B, Caughey A. Role of sequential genetic sonogram and cellfree fetal DNA after EIF detection: A cost-effectiveness analysis. *American Journal of Obstetrics and Gynecology* 2017;**216 (1 Sup**:S95–6.

395 Hacker F, Griffin E, Shaffer B, Caughey A. Role of genetic sonogram and NIPT after EIF detection: A cost-effectiveness analysis. *American Journal of Obstetrics and Gynecology* 2015;**212**:S171–2.

396 Hacker FM, Griffin EE, Shaffer BL, Caughey AB. Role of genetic ultrasonogram after choroid plexus cyst detection a cost-effectiveness analysis. *Obstetrics and Gynecology* 2015;**125**:116S.

397 Harris AH. The cost effectiveness of prenatal ultrasound screening for trisomy 21. *International Journal of Technology Assessment in Health Care* 2004;**20**:464–8.

398 Health Quality O. Noninvasive Prenatal Testing for Trisomies 21, 18, and 13, Sex Chromosome Aneuploidies, and Microdeletions: A Health Technology Assessment. *Ontario Health Technology Assessment Series* 2019;**19**:1–166.

399 Norman R, van Gool K, Hall J, Delatycki M, Massie J. Cost-effectiveness of carrier screening for cystic fibrosis in Australia. *Journal of Cystic Fibrosis* 2012;**11**:281–7.

400 Odibo AO, Stamilio DM, Nelson DB, Sehdev HM, Macones GA. A cost-effectiveness analysis of prenatal screening strategies for Down syndrome. *Obstetrics and Gynecology* 2005;**106**:562–8.

401 Resch S, Altice FL, Paltiel AD. Cost-effectiveness of HIV screening for incarcerated pregnant women. *JAIDS: Journal of Acquired Immune Deficiency Syndromes* 2005;**38**:163–73.

402 Ritchie K, Bradbury I, Slattery J, Wright D, Iqbal K, Penney G. Economic modelling of antenatal screening and ultrasound scanning programmes for identification of fetal abnormalities. *BJOG: An International Journal of Obstetrics & Gynaecology* 2005;**112**:866–74.

403 Udeh B, Udeh C, Graves N. Perinatal HIV transmission and the cost-effectiveness of screening at 14 weeks gestation, at the onset of labour and the rapid testing of infants. *BMC Infectious Diseases* 2008;**8**:174.

404 Vanara F, Bergeretti F, Gaglioti P, Todros T. Economic evaluation of ultrasound screening options for structural fetal malformations. *Ultrasound in Obstetrics and Gynecology* 2004;**24**:633–9.

405 Vintzileos AM, Ananth C v, Smulian JC, Day-Salvatore DL, Beazoglou T, Knuppel RA. Cost-benefit analysis of prenatal diagnosis for Down syndrome using the British or the American approach. *Obstetrics and Gynecology* 2000;**95**:577–83.

406 Xie X, Wang M, Goh ES, Ungar WJ, Little J, Carroll JC, *et al.* Noninvasive Prenatal Testing for Trisomies 21, 18, and 13, Sex Chromosome Aneuploidies, and Microdeletions in Average-Risk Pregnancies: A Cost-Effectiveness Analysis. *Journal of Obstetrics and Gynaecology Canada* 2020;**31**:31.

407 Nielsen R, Gyrd-Hansen D. Prenatal screening for cystic fibrosis: an economic analysis. *Health Econnomics* 2002;**11**:285–99. https://doi.org/10.1002/hec.652.

408 Kessels S, Morona J, Mittal R, Vogan A, Newton S, Schubert C, *et al.* *Testing for hereditary mutations in the cystic fibrosis transmembrane conductance regulator (CFTR) gene*. 2015. URL: https://www1.health.gov.au/internet/msac/publishing.nsf/Content/D54C11627FEA9785CA25801000123BDC/$File/1216-MSAC-CA-CFTR.pdf%0A (Accessed February 20, 2021).

409 Ayres AC, Whitty JA, Ellwood DA. A cost-effectiveness analysis comparing different strategies to implement noninvasive prenatal testing into a Down syndrome screening program. *Australian and New Zealand Journal of Obstetrics & Gynaecology* 2014;**54**:412–7.

410 Bayon JC, Orruno E, Portillo MI, Asua J. The consequences of implementing non-invasive prenatal testing with cell-free foetal DNA for the detection of Down syndrome in the Spanish National Health Service: a cost-effectiveness analysis. *Cost Effectiveness and Resource Allocation* 2019;**17**:6.

411 Bricker L, Garcia J, Henderson J, Mugford M, Neilson J, Roberts T, *et al.* Ultrasound screening in pregnancy: a systematic review of the clinical effectiveness, cost-effectiveness and women’s views. *Health Technology Assessment* 2000;**4**:i–vi, 1.

412 Caughey AB, Kuppermann M, Norton ME, Washington AE. Nuchal translucency and first trimester biochemical markers for down syndrome screening: a cost-effectiveness analysis. *American Journal of Obstetrics and Gynecology* 2002;**187**:1239–45.

413 DeVore GR, Romero R. Genetic sonography: a cost-effective method for evaluating women 35 years and older who decline genetic amniocentesis. *Journal of Ultrasound in Medicine* 2002;**21**:5–13.

414 Di Cianni G, Volpe L, Casadidio I, Bottone P, Marselli L, Lencioni C, *et al.* Universal screening and intensive metabolic management of gestational diabetes: cost-effectiveness in Italy. *Acta Diabetologica* 2002;**39**:69–73.

415 Fairbrother G, Burigo J, Sharon T, Song K. Prenatal screening for fetal aneuploidies with cell-free DNA in the general pregnancy population: a cost-effectiveness analysis. *Journal of Maternal-Fetal and Neonatal Medicine* 2016;**29**:1160–4.

416 Gekas J, van den Berg DG, Durand A, Vallee M, Wildschut HI, Bujold E, *et al.* Rapid testing versus karyotyping in Down’s syndrome screening: cost-effectiveness and detection of clinically significant chromosome abnormalities. *European Journal of Human Genetics* 2011;**19**:3–9.

417 Juvet LK, Ormstad SS, Stoinska-Schneider A, Solberg B, Arentz-Hansen H, Kvamme MK, *et al.* Non invasive prenatal test (NIPT) for identification of trisomy 21, 18 and 13. *Knowledge Centre for the Health Services at The Norwegian Institute of Public Health (NIPH)* 2016;**NIPH Syste**:Executive Summaries.

418 Kaimal AJ, Norton ME, Kuppermann M. Prenatal Testing in the Genomic Age: Clinical Outcomes, Quality of Life, and Costs. *Obstetrics and Gynecology* 2015;**126**:737–46.

419 Nshimyumukiza L, Beaumont JA, Duplantie J, Langlois S, Little J, Audibert F, *et al.* Cell-Free DNA-Based Non-invasive Prenatal Screening for Common Aneuploidies in a Canadian Province: A Cost-Effectiveness Analysis. *Journal of Obstetrics and Gynaecology Canada* 2018;**40**:48–60.

420 Ohno M, Caughey A. The role of noninvasive prenatal testing as a diagnostic versus a screening tool--a cost-effectiveness analysis. *Prenatal Diagnosis* 2013;**33**:630–5.

421 O’Leary P, Maxwell S, Murch A, Hendrie D. Prenatal screening for Down syndrome in Australia: costs and benefits of current and novel screening strategies. *Australian and New Zealand Journal of Obstetrics & Gynaecology* 2013;**53**:425–33.

422 Ong JJ, Chen M, Hocking J, Fairley CK, Carter R, Bulfone L, *et al.* Chlamydia screening for pregnant women aged 16-25 years attending an antenatal service: a cost-effectiveness study. *BJOG: An International Journal of Obstetrics & Gynaecology* 2016;**123**:1194–202.

423 Pinto NM, Nelson R, Puchalski M, Metz TD, Smith KJ. Cost-effectiveness of prenatal screening strategies for congenital heart disease. *Ultrasound in Obstetrics and Gynecology* 2014;**44**:50–7.

424 Song K, Musci TJ, Caughey AB. Clinical utility and cost of non-invasive prenatal testing with cfDNA analysis in high-risk women based on a US population. *Journal of Maternal-Fetal and Neonatal Medicine* 2013;**26**:1180–5.

425 Walker B, Nelson R, Jackson B, Grenache D, Ashwood E, Schmidt R. A Cost-Effectiveness Analysis of First Trimester Non-Invasive Prenatal Screening for Fetal Trisomies in the United States. *PLoS ONE* 2015;**10**:e0131402.

426 Taylor-Phillips S, Freeman K, Geppert J, Madan J, Uthman O, Agbebiyi A, *et al.* *Systematic review and cost-consequence assessment of cell-free DNA testing for T21, T18 and T13 in the UK – Final report*. 2015. URL: https://legacyscreening.phe.org.uk/policydb_download.php?doc=1265%0A (Accessed February 20, 2021).

427 Hoogendoorn M, Hamberg-van Reenen H, van Genugten M, de Wit G, Schielen P. *Vergelijking van kosten en effecten van prenatale screeningsmethoden voor Down syndroom en neuraalbuisdefecten*. 2004. URL: https://www.rivm.nl/bibliotheek/rapporten/230041001.pdf%0A (Accessed February 20, 2021).

428 Institute of Health Economics. *First and second trimester prenatal screening update*. 2014. URL: https://www.ihe.ca/download/first_and_second_trimester_prenatal_screening_update.pdf%0A (Accessed February 20, 2021).

429 Hulstaert F, Neyt M, Gyselaers W. *The non-invasive prenatal test (NIPT) for trisomy 21 – health economic aspects*. 2014. URL: https://kce.fgov.be/sites/default/files/atoms/files/KCE_222_Non_invasive_prenatal_ test_Report.pdf (Accessed February 20, 2021).

430 National Institute for Health and Care Excellence. *Diabetes in pregnancy: Management of diabetes and its complications from preconception to the postnatal period. NG3, Feb 2015 (Appendix N.3)*. 2015. URL: https://www.nice.org.uk/guidance/ng3/evidence/appendices-ag-and-in-pdf-3784286%0A (Accessed February 20, 2021).

431 Boshuizen HC, van der Lem GJ, Kauffman-de Boer MA, van Zanten GA, Oudesluys-Murphy AM, Verkerk PH. Costs of different strategies for neonatal hearing screening: a modelling approach. *Archives of Disease in Childhood: Fetal and Neonatal Edition* 2001;**85**:F177-81.

432 Burke MJ, Shenton RC, Taylor MJ. The economics of screening infants at risk of hearing impairment: an international analysis. *International Journal of Pediatric Otorhinolaryngology* 2012;**76**:212–8.

433 Chicaiza-Becerra L, Garcia-Molina M, Oviedo-Ariza S, Gomez-Marin JE, Gomez-Sanchez PI. Cost effectiveness of various diagnostic strategies for detecting congenital toxoplasmosis in newborns. *Infectio* 2013;**17**:53–60.

434 Hessel F, Grill E, Schnell-Inderst P, Siebert U, Kunze S, Nickisch A, *et al.* Economic evaluation of newborn hearing screening: modelling costs and outcomes. *German Medical Science* 2003;**1**:Doc09.

435 Institute of Health E. Newborn blood spot screening for galactosemia, tyrosinemia type I, homocystinuria, sickle cell anemia, sickle cell/beta-thalassemia, sickle cell/hemoglobin C disease, and severe combined immunodeficiency. *Institute of Health Economics* 2016;**03**:3.

436 Kemper AR, Downs SM. A cost-effectiveness analysis of newborn hearing screening strategies. *Archives of Pediatrics and Adolescent Medicine* 2000;**154**:484–8.

437 Londono Trujillo D, Sandoval Reyes NF, Taborda Restrepo A, Chamorro Velasquez CL, Dominguez Torres MT, Romero Ducuara S V, *et al.* Cost-effectiveness analysis of newborn pulse oximetry screening to detect critical congenital heart disease in Colombia. *Cost Effectiveness and Resource Allocation* 2019;**17**:.

438 Nshimyumukiza L, Bois A, Daigneault P, Lands L, Laberge AM, Fournier D, *et al.* Cost effectiveness of newborn screening for cystic fibrosis: a simulation study. *Journal of Cystic Fibrosis* 2014;**13**:267–74.

439 Raimond V, Sambuc C, Pibouleau L. Ethics Evaluation Revealing Decision-Maker Motives: A Case of Neonatal Screening. *International Journal of Technology Assessment in Health Care* 2018;**34**:189–95.

440 Ramwadhdoebe S, Van Merode GG, Boere-Boonekamp MM, Sakkers RJ, Buskens E. Implementation by simulation; strategies for ultrasound screening for hip dysplasia in the Netherlands. *BMC Health Services Research* 2010;**10**:75.

441 Schmidt M, Werbrouck A, Verhaeghe N, De Wachter E, Simoens S, Annemans L, *et al.* A model-based economic evaluation of four newborn screening strategies for cystic fibrosis in Flanders, Belgium. *Acta Clinica Belgica* 2019;**75**:1–9.

442 Seror V, Cao C, Roussey M, Giorgi R. PAP assays in newborn screening for cystic fibrosis: a population-based cost-effectiveness study. *Journal of Medical Screening* 2016;**23**:62–9.

443 Zupancic JA, Triedman JK, Alexander M, Walsh EP, Richardson DK, Berul CI. Cost-effectiveness and implications of newborn screening for prolongation of QT interval for the prevention of sudden infant death syndrome. *Journal of Pediatrics* 2000;**136**:481–9.

444 Tran K, Banerjee S, Li H, Noorani H, Mensinkai S, Dooley K. *Newborn Screening for Medium Chain Acyl-CoA Dehydrogenase Deficiency Using Tandem Mass Spectrometry: Clinical and Cost-effectiveness*. 2006. URL: https://www.cadth.ca/media/pdf/297_tandemmass_tr_e_no-appendices.pdf%0A (Accessed February 20, 2021).

445 Institute of Health Economics. *Screening Newborns for Hearing*. 2007. URL: https://www.ihe.ca/download/screening_newborns_for_hearing.pdf%0A (Accessed February 20, 2021).

446 Hillman SC, Barton PM, Roberts TE, Maher ER, McMullan DM, Kilby MD. BAC chromosomal microarray for prenatal detection of chromosome anomalies in fetal ultrasound anomalies: an economic evaluation. *Fetal Diagnosis and Therapy* 2014;**36**:49–58.

447 Kott B, Dubinsky TJ. Cost-effectiveness model for first-trimester versus second-trimester ultrasound screening for Down syndrome. *Journal of the American College of Radiology* 2004;**1**:415–21.

448 Le Bras A, Salomon LJ, Bussieres L, Malan V, Elie C, Mahallati H, *et al.* Cost-effectiveness of five prenatal screening strategies for trisomies and other unbalanced chromosomal abnormalities: model-based analysis. *Ultrasound in Obstetrics and Gynecology* 2019;**54**:596–603.

449 Feuchtbaum L, Cunningham G. Economic evaluation of tandem mass spectrometry screening in California. *Pediatrics* 2006;**117**:S280-6.

450 Griebsch I, Knowles RL, Brown J, Bull C, Wren C, Dezateux CA. Comparing the clinical and economic effects of clinical examination, pulse oximetry, and echocardiography in newborn screening for congenital heart defects: a probabilistic cost-effectiveness model and value of information analysis. *International Journal of Technology Assessment in Health Care* 2007;**23**:192–204.

451 Kezirian EJ, White KR, Yueh B, Sullivan SD. Cost and cost-effectiveness of universal screening for hearing loss in newborns. *Otolaryngology - Head and Neck Surgery* 2001;**124**:359–67.

452 Knowles R, Griebsch I, Dezateux C, Brown J, Bull C, Wren C. Newborn screening for congenital heart defects: a systematic review and cost-effectiveness analysis. *Health Technology Assessment* 2005;**9**:1–152, iii.

453 Lanting CI, van Tijn DA, Loeber JG, Vulsma T, de Vijlder JJ, Verkerk PH. Clinical effectiveness and cost-effectiveness of the use of the thyroxine/thyroxine-binding globulin ratio to detect congenital hypothyroidism of thyroidal and central origin in a neonatal screening program. *Pediatrics* 2005;**116**:168–73.

454 Narayen IC, Te Pas AB, Blom NA, van den Akker-van Marle ME. Cost-effectiveness analysis of pulse oximetry screening for critical congenital heart defects following homebirth and early discharge. *European Journal of Pediatrics* 2019;**178**:97–103.

455 Roberts T, Pickering K, Barton P, Ewer A. *Pulse oximetry as a screening test for critical congenital heart defects and other significant diagnoses in newborn infants: a cost effectiveness analysis*. 2019. URL: https://legacyscreening.phe.org.uk/documents/pulse-oximetry/PO Health Economics Report.pdf%0A (Accessed February 20, 2021).

456 Hubbard HB. Expanded newborn screening for genetic and metabolic disorders: modeling costs and outcomes. *Nursing Economics* 2007;**25**:345–52.

457 Hopkins MK, Dugoff L, Durnwald C, Havrilesky LJ, Dotters-Katz S. Cell-free DNA for Down syndrome screening in obese women: Is it a cost-effective strategy? *Prenatal Diagnosis* 2020;**40**:173–8.

458 Sinkey RG, Odibo AO. Vasa previa screening strategies: decision and cost-effectiveness analysis. *Ultrasound in Obstetrics and Gynecology* 2018;**52**:522–9.

459 Mukerji A, Shafey A, Jain A, Cohen E, Shah PS, Sander B, *et al.* Pulse oximetry screening for critical congenital heart defects in Ontario, Canada: a cost-effectiveness analysis. *Canadian Journal of Public Health* 2020;**06**:6.

460 De Laet C, Hanquet G, Hendrickx E. *Multi criteria decision analysis to select priority diseases for newborn blood screening*. 2016. URL: https://kce.fgov.be/sites/default/files/atoms/files/KCE_267_Newborn_blood_screening.pdf%0A (Accessed February 20, 2021).

461 Cipriano LE, Rupar CA, Zaric GS. The cost-effectiveness of expanding newborn screening for up to 21 inherited metabolic disorders using tandem mass spectrometry: results from a decision-analytic model. *Value in Health* 2007;**10**:83–97.

462 Bergevin A, Zick CD, McVicar SB, Park AH. Cost-benefit analysis of targeted hearing directed early testing for congenital cytomegalovirus infection. *International Journal of Pediatric Otorhinolaryngology* 2015;**79**:2090–3.

463 Bonds DE, Freedberg KA. Cost-effectiveness of prenatal screening for postpartum thyroiditis. *Journal of Womens Health and Gender-Based Medicine* 2001;**10**:649–58.

464 Dosiou C, Sanders GD, Araki SS, Crapo LM. Screening pregnant women for autoimmune thyroid disease: a cost-effectiveness analysis. *European Journal of Endocrinology* 2008;**158**:841-851\.

465 Tasillo A, Eftekhari Yazdi G, Nolen S, Schillie S, Vellozzi C, Epstein R, *et al.* Short-Term Effects and Long-Term Cost-Effectiveness of Universal Hepatitis C Testing in Prenatal Care. *Obstetrics and Gynecology* 2019;**133**:289–300.

466 Bessey A, Chilcott JB, Leaviss J, Sutton A. Economic impact of screening for X-linked Adrenoleukodystrophy within a newborn blood spot screening programme. *Orphanet Journal Of Rare Diseases* 2018;**13**:179.

467 Dritsaki M, Rivero-Arias O, Gray A, Ball C, Nanchahal J. What do we know about managing Dupuytren’s disease cost-effectively? *BMC Musculoskeletal Disorders* 2018;**19**:34. https://doi.org/10.1186/s12891-018-1949-2.

468 Peterson C, Grosse SD, Oster ME, Olney RS, Cassell CH. Cost-effectiveness of routine screening for critical congenital heart disease in US newborns. *Pediatrics* 2013;**132**:e595-603.

469 Tiwana SK, Rascati KL, Park H. Cost-effectiveness of expanded newborn screening in Texas. *Value in Health* 2012;**15**:613–21.

470 Turcotte C, Blancquaert I, Brabant J, St-Louis M. *Évaluation de la pertinence du dépistage néonatal sanguin par spectrométrie de masse en tandem de l’homocystinurie classique (HCY)*. 2020. URL: https://www.inesss.qc.ca/fileadmin/doc/INESSS/Rapports/Depistage/INESSS_DepistageNeonatal_HCY.pdf%0A (Accessed February 20, 2021).

471 Cahill AG, Odibo AO, Stamilio DM, Macones GA. Screening and treating for primary cytomegalovirus infection in pregnancy: where do we stand? A decision-analytic and economic analysis. *American Journal of Obstetrics and Gynecology* 2009;**201**:466.e1-7.

472 Ohno M, Cheng YW, Shaffer B, Caughey AB. A new test to diagnose Down Syndrome using maternal serum - At what specificity, sensitivity, and cost is it cost-effective? *American Journal of Obstetrics and Gynecology* 2011;**204 (1 SUP**:S236–7.

473 Chowers M, Shavit O. Economic evaluation of universal prenatal HIV screening compared with current “at risk” policy in a very low prevalence country. *Sexually Transmitted Infections* 2017;**93**:112–7.

474 Cipriano LE, Barth Jr. WH, Zaric GS. The cost-effectiveness of targeted or universal screening for vasa praevia at 18-20 weeks of gestation in Ontario. *BJOG: An International Journal of Obstetrics & Gynaecology* 2010;**117**:1108–18.

475 Darlington M, Carbonne B, Mailloux A, Brossard Y, Levy-Mozziconacci A, Cortey A, *et al.* Effectiveness and costs of non-invasive foetal RHD genotyping in rhesus-D negative mothers: a French multicentric two-arm study of 850 women. *BMC Pregnancy and Childbirth* 2018;**18**:496.

476 Farrar D, Simmonds M, Griffin S, Duarte A, Lawlor DA, Sculpher M, *et al.* The identification and treatment of women with hyperglycaemia in pregnancy: an analysis of individual participant data, systematic reviews, meta-analyses and an economic evaluation. *Health Technology Assessment* 2016;**20**:1–348.

477 Gantt S, Dionne F, Kozak FK, Goshen O, Goldfarb DM, Park AH, *et al.* Cost-effectiveness of Universal and Targeted Newborn Screening for Congenital Cytomegalovirus Infection. *JAMA Pediatrics* 2016;**170**:1173–80.

478 Hauspurg A, Albright C, Rouse D, Werner E. Third trimester fetal growth ultrasound: A cost-benefit analysis. *American Journal of Obstetrics and Gynecology* 2015;**212**:S285.

479 Hersh AR, Megli CJ, Caughey AB. Repeat Screening for Syphilis in the Third Trimester of Pregnancy: A Cost-Effectiveness Analysis. *Obstetrics and Gynecology* 2018;**132**:699–707.

480 Imaz-Iglesia I, Miguel LGS, Ayala-Morillas LE, Garcia-Perez L, Gonzalez-Enriquez J, Blasco-Hernandez T, *et al.* Economic evaluation of chagas disease screening in Spain. *Acta Tropica* 2015;**148**:77–88.

481 Kowada A. Cost effectiveness of interferon-gamma release assay for TB screening of HIV positive pregnant women in low TB incidence countries. *Journal of Infection* 2014;**68**:32–42.

482 Marseille E, Lohse N, Jiwani A, Hod M, Seshiah V, Yajnik CS, *et al.* The cost-effectiveness of gestational diabetes screening including prevention of type 2 diabetes: application of a new model in India and Israel. *Journal of Maternal-Fetal and Neonatal Medicine* 2013;**26**:802–10.

483 Mistry H, Gardiner HM. The cost-effectiveness of prenatal detection for congenital heart disease using telemedicine screening. *Journal of Telemedicine and Telecare* 2013;**19**:190–6.

484 Plunkett BA, Grobman WA. Routine hepatitis C virus screening in pregnancy: a cost-effectiveness analysis. *American Journal of Obstetrics and Gynecology* 2005;**192**:1153–61.

485 Round JA, Jacklin P, Fraser RB, Hughes RG, Mugglestone MA, Holt RI. Screening for gestational diabetes mellitus: cost-utility of different screening strategies based on a woman’s individual risk of disease. *Diabetologia* 2011;**54**:256–63.

486 Rozenbaum MH, Verweel G, Folkerts DK, Dronkers F, van den Hoek JA, Hartwig NG, *et al.* Cost-effectiveness estimates for antenatal HIV testing in the Netherlands. *International Journal of STD and AIDS* 2008;**19**:668–75.

487 Selvapatt N, Ward T, Bailey H, Bennett H, Thorne C, See LM, *et al.* Is antenatal screening for hepatitis C virus cost-effective? A decade’s experience at a London centre. *Journal of Hepatology* 2015;**63**:797–804.

488 Shmueli A, Meiri H, Gonen R. Economic assessment of screening for pre-eclampsia. *Prenatal Diagnosis* 2012;**32**:29–38.

489 Turner ML, Bessos H, Fagge T, Harkness M, Rentoul F, Seymour J, *et al.* Prospective epidemiologic study of the outcome and cost-effectiveness of antenatal screening to detect neonatal alloimmune thrombocytopenia due to anti-HPA-1a. *Transfusion* 2005;**45**:1945–56.

490 Wetzel S, Miller ES, Cirino N, Dukhovny D, Ameel B, Caughey AB. Routine antenatal screening for depression: What are the outcomes and is it cost-effective? *American Journal of Obstetrics and Gynecology* 2016;**214**:S383–4.

491 Wu O, Robertson L, Twaddle S, Lowe GD, Clark P, Greaves M, *et al.* Screening for thrombophilia in high-risk situations: systematic review and cost-effectiveness analysis. The Thrombosis: Risk and Economic Assessment of Thrombophilia Screening (TREATS) study. *Health Technology Assessment* 2006;**10**:1–110.

492 Zhang W, Mohammadi T, Sou J, Anis AH. Cost-effectiveness of prenatal screening and diagnostic strategies for Down syndrome: A microsimulation modeling analysis. *PLoS ONE* 2019;**14**:e0225281.

493 Eijsink JFH, al Khayat M, Boersma C, ter Horst PGJ, Wilschut JC, Postma MJ. Cost-effectiveness of hepatitis C virus screening, and subsequent monitoring or treatment among pregnant women in the Netherlands. *European Journal of Health Economics* 2021;**22**:75–88. https://doi.org/10.1007/s10198-020-01236-2.

494 National Collaborating Centre for Women’s and Children’s Health. *Antenatal care routine care for the healthy pregnant woman (Appendix G)*. 2008. URL: https://stratog.rcog.org.uk/sites/default/files/Induction of labour and  prolonged pregnancy/nice_antenatalcare_62_2008_pg15.pdf%0A (Accessed February 20, 2021).

495 Carroll AE, Downs SM. Comprehensive cost-utility analysis of newborn screening strategies. *Pediatrics* 2006;**117**:S287-95.

496 Castillo-Riquelme MC, Lord J, Moseley MJ, Fielder AR, Haines L. Cost-effectiveness of digital photographic screening for retinopathy of prematurity in the United Kingdom. *International Journal of Technology Assessment in Health Care* 2004;**20**:201–13.

497 Chan K, Davis J, Pai SY, Bonilla FA, Puck JM, Apkon M. A Markov model to analyze cost-effectiveness of screening for severe combined immunodeficiency (SCID). *Molecular Genetics and Metabolism* 2011;**104**:383–9.

498 Ding Y, Thompson JD, Kobrynski L, Ojodu J, Zarbalian G, Grosse SD. Cost-Effectiveness/Cost-Benefit Analysis of Newborn Screening for Severe Combined Immune Deficiency in Washington State. *Journal of Pediatrics* 2016;**172**:127–35.

499 Fox DA, Ronsley R, Khowaja AR, Haim A, Vallance H, Sinclair G, *et al.* Clinical Impact and Cost Efficacy of Newborn Screening for Congenital Adrenal Hyperplasia. *Journal of Pediatrics* 2020;**07**:7.

500 Hamers FF, Rumeau-Pichon C. Cost-effectiveness analysis of universal newborn screening for medium chain acyl-CoA dehydrogenase deficiency in France. *BMC Pediatrics* 2012;**12**:60.

501 Herrero C, Moreno-Ternero JD. Hospital Costs and Social Costs: A Case Study of Newborn Hearing Screening. *Investigaciones Economicas* 2005;**29**:203–16.

502 Insinga RP, Laessig RH, Hoffman GL. Newborn screening with tandem mass spectrometry: examining its cost-effectiveness in the Wisconsin Newborn Screening Panel. *Journal of Pediatrics* 2002;**141**:524–31.

503 McGhee SA, Stiehm ER, McCabe ER. Potential costs and benefits of newborn screening for severe combined immunodeficiency. *Journal of Pediatrics* 2005;**147**:603–8.

504 Pandor A, Eastham J, Beverley C, Chilcott J, Paisley S. Clinical effectiveness and cost-effectiveness of neonatal screening for inborn errors of metabolism using tandem mass spectrometry: a systematic review. *Health Technology Assessment* 2004;**8**:iii, 1–121.

505 Panepinto JA, Magid D, Rewers MJ, Lane PA. Universal versus targeted screening of infants for sickle cell disease: a cost-effectiveness analysis. *Journal of Pediatrics* 2000;**136**:201–8.

506 Pfeil J, Listl S, Hoffmann GF, Kolker S, Lindner M, Burgard P. Newborn screening by tandem mass spectrometry for glutaric aciduria type 1: a cost-effectiveness analysis. *Orphanet Journal Of Rare Diseases* 2013;**8**:167.

507 Prosser LA, Kong CY, Rusinak D, Waisbren SL. Projected costs, risks, and benefits of expanded newborn screening for MCADD. *Pediatrics* 2010;**125**:e286-94.

508 Schoen EJ, Baker JC, Colby CJ, To TT. Cost-benefit analysis of universal tandem mass spectrometry for newborn screening. *Pediatrics* 2002;**110**:781–6.

509 Schreiber RA, Masucci L, Kaczorowski J, Collet JP, Lutley P, Espinosa V, *et al.* Home-based screening for biliary atresia using infant stool colour cards: a large-scale prospective cohort study and cost-effectiveness analysis. *Journal of Medical Screening* 2014;**21**:126–32.

510 Simpson N, Anderson R, Sassi F, Pitman A, Lewis P, Tu K, *et al.* The cost-effectiveness of neonatal screening for cystic fibrosis: an analysis of alternative scenarios using a decision model. *Cost Effectiveness and Resource Allocation* 2005;**3**:8.

511 Vallejo-Torres L, Castilla I, Couce ML, Perez-Cerda C, Martin-Hernandez E, Pineda M, *et al.* Cost-Effectiveness Analysis of a National Newborn Screening Program for Biotinidase Deficiency. *Pediatrics* 2015;**136**:e424-32.

512 van der Hilst CS, Derks TG, Reijngoud DJ, Smit GP, TenVergert EM. Cost-effectiveness of neonatal screening for medium chain acyl-CoA dehydrogenase deficiency: the homogeneous population of The Netherlands. *Journal of Pediatrics* 2007;**151**:115–20, 120.e1.

513 Van der Ploeg CPB, Blom M, Bredius RGM, van der Burg M, Schielen P, Verkerk PH, *et al.* Cost-effectiveness of newborn screening for severe combined immunodeficiency. *European Journal of Pediatrics* 2019;**178**:721–9.

514 van der Ploeg CP, van den Akker-van Marle ME, Vernooij-van Langen AM, Elvers LH, Gille JJ, Verkerk PH, *et al.* Cost-effectiveness of newborn screening for cystic fibrosis determined with real-life data. *Journal of Cystic Fibrosis* 2015;**14**:194–202.

515 Venditti LN, Venditti CP, Berry GT, Kaplan PB, Kaye EM, Glick H, *et al.* Newborn screening by tandem mass spectrometry for medium-chain Acyl-CoA dehydrogenase deficiency: a cost-effectiveness analysis. *Pediatrics* 2003;**112**:1005–15.

516 Yoo B, Grosse S. The cost effectiveness of screening newborns for congenital adrenal hyperplasia. *Public Health Genomics* 2009;**12**:67–72.

517 Zupancic JAF, Ying GS, de Alba Campomanes A, Tomlinson LA, Binenbaum G, Group GRS. Evaluation of the economic impact of modified screening criteria for retinopathy of prematurity from the Postnatal Growth and ROP (G-ROP) study. *Journal of Perinatology* 2020;**40**:1100–8. https://doi.org/10.1038/s41372-020-0605-5.

518 Bessey A, Chilcott J, Pandor A, Paisley S. The Cost-Effectiveness of Expanding the UK Newborn Bloodspot Screening Programme to Include Five Additional Inborn Errors of Metabolism. *International Journal of Neonatal Screening* 2020;**6**:. https://doi.org/10.3390/ijns6040093.

519 Richardson JS, Kemper AR, Grosse SD, Lam WKK, Rose AM, Ahmad A, *et al.* Health and economic outcomes of newborn screening for infantile-onset Pompe disease. *Genetics in Medicine* 2020. https://doi.org/10.1038/s41436-020-01038-0.

520 Mogul D, Zhou M, Intihar P, Schwarz K, Frick K. Cost-effective analysis of screening for biliary atresia with the stool color card. *Journal of Pediatric Gastroenterology and Nutrition* 2015;**60**:91–8. https://doi.org/10.1097/MPG.0000000000000569.

521 Bessey A, Chilcott J, Leaviss J, de la Cruz C, Wong R. A Cost-Effectiveness Analysis of Newborn Screening for Severe Combined Immunodeficiency in the UK. *International Journal of Neonatal Screening* 2019;**5**:28. https://doi.org/10.3390/ijns5030028.

522 Health Partners Consulting Group. *Cost-effectiveness of newborn screening for Severe Combined Immune Deficiency*. 2014. URL: https://www.nsu.govt.nz/system/files/resources/cost-effectiveness-newborn-screening-severe-combined-immune-deficiency.pdf%0A (Accessed February 20, 2021).

523 Ball RH, Caughey AB, Malone FD, Nyberg DA, Comstock CH, Saade GR, *et al.* First- and second-trimester evaluation of risk for Down syndrome. *Obstetrics and Gynecology* 2007;**110**:10–7.

524 Hollingsworth B, Harris A. Economic evaluation of prenatal population screening for fragile X syndrome. *Community Genetics* 2005;**8**:68–72.

525 Musci TJ, Caughey AB. Cost-effectiveness analysis of prenatal population-based fragile X carrier screening. *American Journal of Obstetrics and Gynecology* 2005;**192**:1905–12; discussion 1912.

526 Wald NJ, Rudnicka AR, Bestwick JP. Sequential and contingent prenatal screening for Down syndrome. *Prenatal Diagnosis* 2006;**26**:769–77.

527 Han C, Werner E, Pettker C, Norwitz E, Funai E, Thung S. Universal antenatal screening for spinal muscular atrophy (SMA): A cost-utility analysis. *American Journal of Obstetrics and Gynecology* 2009;**201**:S261.

528 Boyd KA, Briggs AH, Fenwick E, Norrie J, Stock S. Power and sample size for cost-effectiveness analysis: fFN neonatal screening. *Contemporary Clinical Trials* 2011;**32**:893–901.

529 Cahill AG, Odibo AO, Caughey AB, Stamilio DM, Hassan SS, Macones GA, *et al.* Universal cervical length screening and treatment with vaginal progesterone to prevent preterm birth: a decision and economic analysis. *American Journal of Obstetrics and Gynecology* 2010;**202**:548.e1-8.

530 Berger LM. Estimating the benefits and costs of a universal substance abuse screening and treatment referral policy for pregnant women. *Journal of Social Service Research* 2002;**29**:57–84. https://doi.org/10.1300/J079v29n01_03.

531 Killie MK. Cost-effectiveness of antenatal screening for neonatal alloimmune thrombocytopenia (vol 114, pg 588, 2007). *BJOG: An International Journal of Obstetrics & Gynaecology* 2007;**114**:1453. https://doi.org/10.1111/j.1471-0528.2007.01551.x.

532 Albright CM, MacGregor C, Sutton D, Theva M, Hughes BL, Werner EF. Group B Streptococci Screening Before Repeat Cesarean Delivery: A Cost-Effectiveness Analysis. *Obstetrics and Gynecology* 2017;**129**:111–9.

533 Kanga I, Williams D, Hatchette T, MacKinnon S, Jung H, Black C, *et al.* Screening for Chlamydia Trachomatis and Neisseria Gonorrhoeae During Pregnancy: A Health Technology Assessment. *Canadian Agency for Drugs and Technologies in Health* 2018.

534 Bak GS, Shaffer BL, Madriago E, Allen A, Kelly B, Caughey AB, *et al.* Impact of maternal obesity on fetal cardiac screening: which follow-up strategy is cost-effective? *Ultrasound in Obstetrics and Gynecology* 2019;**15**:.

535 Baker D, Brown Z, Hollier LM, Wendel GD, Hulme L, Griffiths DA, *et al.* Cost-effectiveness of herpes simplex virus type 2 serologic testing and antiviral therapy in pregnancy. *American Journal of Obstetrics and Gynecology* 2004;**191**:2074-2084\.

536 Colbourn T, Asseburg C, Bojke L, Philips Z, Claxton K, Ades A, *et al.* Prenatal screening and treatment strategies to prevent group B streptococcal and other bacterial infections in early infancy: cost-effectiveness and expected value of information analyses. *Health Technology Assessment* 2007;**11**:1–226.

537 Danyliv A, Gillespie P, O’Neill C, Tierney M, O’Dea A, McGuire BE, *et al.* The cost-effectiveness of screening for gestational diabetes mellitus in primary and secondary care in the Republic of Ireland. *Diabetologia* 2016;**59**:436–44.

538 Miller ES, Grobman WA. Cost-effectiveness of transabdominal ultrasound for cervical length screening for preterm birth prevention. *American Journal of Obstetrics and Gynecology* 2013;**209**:546.e1-6.

539 Mission JF, Ohno MS, Cheng YW, Caughey AB. Gestational diabetes screening with the new IADPSG guidelines: a cost-effectiveness analysis. *American Journal of Obstetrics and Gynecology* 2012;**207**:326.e1-9.

540 Mone F, O’Mahony JF, Tyrrell E, Mulcahy C, McParland P, Breathnach F, *et al.* Preeclampsia Prevention Using Routine Versus Screening Test-Indicated Aspirin in Low-Risk Women. *Hypertension* 2018;**72**:1391–6.

541 Nicholson WK, Fleisher LA, Fox HE, Powe NR. Screening for gestational diabetes mellitus: a decision and cost-effectiveness analysis of four screening strategies. *Diabetes Care* 2005;**28**:1482–4.

542 Straub HL, Antoniewicz LW, Riggs JW, Plunkett BA, Hollier LM. Cost-effectiveness analysis of rubella screening strategies using electronic medical records. *American Journal of Perinatology* 2013;**30**:759–64.

543 Thung SF, Grobman WA. The cost-effectiveness of routine antenatal screening for maternal herpes simplex virus-1 and -2 antibodies. *American Journal of Obstetrics and Gynecology* 2005;**192**:483–8.

544 Tuite AR, McCabe CJ, Ku J, Fisman DN. Projected cost-savings with herpes simplex virus screening in pregnancy: towards a new screening paradigm. *Sexually Transmitted Infections* 2011;**87**:141–8.

545 Walker A, Caughey A. Positivity thresholds of HbA1c assay as a screening test for diabetes mellitus in the first trimester in high-risk populations. *Journal of Maternal-Fetal and Neonatal Medicine* 2020:1–5.

546 Wastlund D, Moraitis AA, Thornton JG, Sanders J, White IR, Brocklehurst P, *et al.* The cost-effectiveness of universal late-pregnancy screening for macrosomia in nulliparous women: a decision analysis. *BJOG: An International Journal of Obstetrics & Gynaecology* 2019;**126**:1243–50. https://doi.org/10.1111/1471-0528.15809.

547 Werner EF, Pettker CM, Zuckerwise L, Reel M, Funai EF, Henderson J, *et al.* Screening for gestational diabetes mellitus: are the criteria proposed by the international association of the Diabetes and Pregnancy Study Groups cost-effective? *Diabetes Care* 2012;**35**:529–35.

548 Ontario H. Noninvasive Fetal RhD Blood Group Genotyping: A Health Technology Assessment. *Ontario Health Technology Assessment Series* 2020;**20**:1–160.

549 Dunbar JA, Hsu V, Christensen M, Black B, Williams P, Beauchamp G. Cost-utility analysis of screening and laser treatment of retinopathy of prematurity. *Journal of American Association for Pediatric Ophthalmology and Strabismus* 2009;**13**:186–90.

550 Hopkins RB, Paradis J, Roshankar T, Bowen J, Tarride JE, Blackhouse G, *et al.* Universal or targeted screening for fetal alcohol exposure: a cost-effectiveness analysis. *Journal of Studies on Alcohol and Drugs* 2008;**69**:510–9.

551 Malec LM, Sidonio Jr. RF, Smith KJ, Cooper JD. Three cost-utility analyses of screening for intracranial hemorrhage in neonates with hemophilia. *Journal of Pediatric Hematology/Oncology* 2014;**36**:474–9.

552 Socialstyrelsen. *Screening för X-bunden adrenoleukodystrofi (X-ALD)*. 2020. URL: https://www.socialstyrelsen.se/globalassets/sharepoint-dokument/artikelkatalog/nationella-screeningprogram/2020-12-7078-halsoekonomisk-analys.pdf%0A (Accessed February 20, 2021).

553 Postma MJ, van den Hoek JA, Beck EJ, Heeg B, Jager JC, Coutinho RA. Pharmaco-economic evaluation of mandatory HIV-screening in pregnancy; a cost-efficacy analysis in Amsterdam. *Nederlands Tijdschrift Voor Geneeskunde* 2000;**144**:749–54.

554 Urbanus AT, van Keep M, Matser AA, Rozenbaum MH, Weegink CJ, van den Hoek A, *et al.* Is adding HCV screening to the antenatal national screening program in Amsterdam, the Netherlands, cost-effective? *PLoS ONE* 2013;**8**:e70319\.

555 Modell V, Knaus M, Modell F. An analysis and decision tool to measure cost benefit of newborn screening for severe combined immunodeficiency (SCID) and related T-cell lymphopenia. *Immunologic Research* 2014;**60**:145–52.

556 Quaglini S, Rognoni C, Spazzolini C, Priori SG, Mannarino S, Schwartz PJ. Cost-effectiveness of neonatal ECG screening for the long QT syndrome. *European Heart Journal* 2006;**27**:1824–32.

557 Shermock KM, Gildea TR, Singer M, Stoller JK. Cost-effectiveness of population screening for alpha-1 antitrypsin deficiency: a decision analysis. *COPD: Journal of Chronic Obstructive Pulmonary Disease* 2005;**2**:411–8.

558 Yeh J, Stout NK, Chaudhry A, Gooch M, McMahon P, Christensen KD, *et al.* Population-based cancer predisposition testing as a component of newborn screening: A costeffectiveness analysis. *Journal of Clinical Oncology* 2019;**37**:.

559 Bak GS, Shaffer BL, Madriago E, Allen A, Kelly B, Caughey AB, *et al.* Detection of fetal cardiac anomalies: is increasing the number of cardiac views cost-effective? *Ultrasound in Obstetrics and Gynecology* 2020;**16**:16.

560 Donnay Candil S, Balsa Barro JA, Alvarez Hernandez J, Crespo Palomo C, Perez-Alcantara F, Polanco Sanchez C. Cost-effectiveness analysis of universal screening for thyroid disease in pregnant women in Spain. *Endocrinologia y Nutricion* 2015;**62**:322–30.

561 Dorius A, Worstell T, Griffin E, Little S, Sparks T, Caughey A. Routine antenatal testing for pregnancies at 40 weeks gestation: A cost-effectiveness analysis. *American Journal of Obstetrics and Gynecology* 2014;**210**:S214–5.

562 Ginsberg GM, Eidelman AI, Shinwell E, Anis E, Peyser R, Lotan Y. Should Israel screen all mothers-to-be to prevent early-onset of neonatal group B streptococcal disease? A cost-utility analysis. *Israel Journal of Health Policy Research* 2013;**2**:.

563 Hacker F, Griffin E, Shaffer B, Caughey AB. Role of genetic sonogram after EIF detection: A cost-effectiveness analysis. *Obstetrics and Gynecology* 2017;**129 (Suppl**:165S-166S.

564 Lee BY, Wiringa AE, Mitgang EA, McGlone SM, Afriyie AN, Song Y, *et al.* Routine pre-cesarean Staphylococcus aureus screening and decolonization: a cost-effectiveness analysis. *American Journal of Managed Care* 2011;**17**:693-700\.

565 Odibo AO, Coassolo KM, Stamilio DM, Ural SH, Macones GA. Should all pregnant diabetic women undergo a fetal echocardiography? A cost-effectiveness analysis comparing four screening strategies. *Prenatal Diagnosis* 2006;**26**:39–44.

566 Rours GI, Smith-Norowitz TA, Ditkowsky J, Hammerschlag MR, Verkooyen RP, de Groot R, *et al.* Cost-effectiveness analysis of Chlamydia trachomatis screening in Dutch pregnant women. *Pathogens and Global Health* 2016;**110**:292–302.

567 Scott RK, Crochet S, Huang CC. Universal Rapid Human Immunodeficiency Virus Screening at Delivery: A Cost-Effectiveness Analysis. *Infectious Diseases in Obstetrics and Gynecology* 2018;**2018**:6024698.

568 Sicuri E, Munoz J, Pinazo MJ, Posada E, Sanchez J, Alonso PL, *et al.* Economic evaluation of Chagas disease screening of pregnant Latin American women and of their infants in a non endemic area. *Acta Tropica* 2011;**118**:110–7.

569 Thung SF, Funai EF, Grobman WA. The cost-effectiveness of universal screening in pregnancy for subclinical hypothyroidism. *American Journal of Obstetrics and Gynecology* 2009;**200**:267-269\.

570 Turrentine MA, Ramirez MM, Mastrobattista JM. Cost-effectiveness of universal prophylaxis in pregnancy with prior group B streptococci colonization. *Infectious Diseases in Obstetrics and Gynecology* 2009;**2009**:934698.

571 Hacker FM, Hersh AR, Shaffer BL, Caughey AB. Isolated echogenic intracardiac foci and the role of cell-free fetal DNA: A cost-effectiveness analysis. *Prenatal Diagnosis* 2020;**40**:1517–24. https://doi.org/10.1002/pd.5803.

572 Albright CM, Werner EF, Hughes BL. Cytomegalovirus Screening in Pregnancy: A Cost-Effectiveness and Threshold Analysis. *American Journal of Perinatology* 2019;**36**:678–87.

573 Neovius M, Tiblad E, Westgren M, Kublickas M, Neovius K, Wikman A. Cost-effectiveness of first trimester non-invasive fetal RHD screening for targeted antenatal anti-D prophylaxis in RhD-negative pregnant women: a model-based analysis. *BJOG: An International Journal of Obstetrics & Gynaecology* 2016;**123**:1337–46. https://doi.org/10.1111/1471-0528.13801.

574 Ditkowsky J, Shah KH, Hammerschlag MR, Kohlhoff S, Smith-Norowitz TA. Cost-benefit analysis of Chlamydia trachomatis screening in pregnant women in a high burden setting in the United States. *BMC Infectious Diseases* 2017;**17**:155.

575 Doyle NM, Gardner MO. Prenatal cystic fibrosis screening in Mexican Americans: An economic analysis. *American Journal of Obstetrics and Gynecology* 2003;**189**:769–74.

576 el Helali N, Giovangrandi Y, Guyot K, Chevet K, Gutmann L, Durand-Zaleski I. Cost and effectiveness of intrapartum group B streptococcus polymerase chain reaction screening for term deliveries. *Obstetrics and Gynecology* 2012;**119**:822–9.

577 Kekki M, Kurki T, Kotomaki T, Sintonen H, Paavonen J. Cost-effectiveness of screening and treatment for bacterial vaginosis in early pregnancy among women at low risk for preterm birth. *Acta Obstetricia et Gynecologica Scandinavica* 2004;**83**:27–36.

578 Postma MJ, Bakker A, Welte R, Van Bergen JEAM, Van den Hoek JAR, De Jong-Van den Berg LTW, *et al.* Screening for asymptomatic infection with Chlamydia trachomatis in pregnancy; favourable cost-effectiveness if prevalence is 3% or more. [Dutch]. *Nederlands Tijdschrift Voor Geneeskunde* 2000;**144**:2350–4.

579 Binquet C, Lejeune C, Seror V, Peyron F, Bertaux AC, Scemama O, *et al.* The cost-effectiveness of neonatal versus prenatal screening for congenital toxoplasmosis. *PLoS ONE* 2019;**14**:e0221709.

580 Haberland CA, Benitz WE, Sanders GD, Pietzsch JB, Yamada S, Nguyen L, *et al.* Perinatal screening for group B streptococci: cost-benefit analysis of rapid polymerase chain reaction. *Pediatrics* 2002;**110**:471–80.

581 Picchiassi E, Coata G, Babucci G, Giardina I, Summa V, Tarquini F, *et al.* Intrapartum test for detection of Group B Streptococcus colonization during labor. *Journal of Maternal-Fetal and Neonatal Medicine* 2018;**31**:3293–300.

582 Geelhoed EA, Lewis B, Hounsome D, O’Leary P. Economic evaluation of neonatal screening for phenylketonuria and congenital hypothyroidism. *Journal of Paediatrics and Child Health* 2005;**41**:575–9.

583 Magnusson G, Persson U. Screening for congenital cataracts: a cost-consequence analysis of eye examination at maternity wards in comparison to well-baby clinics. *Acta Paediatrica* 2005;**94**:1089–95.

584 Brancazio L, Paglia M, Kuller J, Wells S. Prenatal Down syndrome screening: A cost analysis of different strategies. *American Journal of Obstetrics and Gynecology* 2003;**189**:S115–S115. https://doi.org/10.1016/j.ajog.2003.10.192.

585 Stillwaggon E, Carrier CS, Sautter M, McLeod R. Maternal serologic screening to prevent congenital toxoplasmosis: a decision-analytic economic model. *PLoS Neglected Tropical Diseases* 2011;**5**:e1333.

586 Clifford C, House M, Ashley L. 660 Prenatal aneuploidy screening in a low-risk hispanic population: price elasticity and cost-effectiveness. *American Journal of Obstetrics and Gynecology* 2021;**224**:. https://doi.org/10.1016/j.ajog.2020.12.684.

587 Avram CM, Dyer AL, Shaffer BL, Caughey AB. The cost-effectiveness of genotyping versus sequencing for prenatal cystic fibrosis carrier screening. *Prenatal Diagnosis* 2021;**41**:1449–59. https://doi.org/10.1002/pd.6027.

588 Chatroux IC, Hersh AR, Caughey AB. Herpes Simplex Virus Serotyping in Pregnant Women With a History of Genital Herpes and an Outbreak in the Third Trimester of Pregnancy: A Cost-Effectiveness Analysis. *Obstetrics and Gynecology* 2021;**137**:63–71. https://doi.org/10.1097/AOG.0000000000004181.

589 Grabner M, Burchard J, Nguyen C, Chung H, Gangan N, Boniface JJ, *et al.* Cost-Effectiveness of a Proteomic Test for Preterm Birth Prediction. *ClinicoEconomics and Outcomes Research* 2021;**13**:809–20. https://doi.org/10.2147/CEOR.S325094.

590 McCormick CA, Domegan L, Carty PG, Drew R, McAuliffe FM, O’Donohoe O, *et al.* Routine screening for hepatitis C in pregnancy is cost-effective in a large urban population in Ireland: a retrospective study. *BJOG: An International Journal of Obstetrics & Gynaecology* 2021. https://doi.org/10.1111/1471-0528.16984.

591 van den Akker-van Marle ME, Blom M, van der Burg M, Bredius RGM, Van der Ploeg CPB. Economic Evaluation of Different Screening Strategies for Severe Combined Immunodeficiency Based on Real-Life Data. *International Journal of Neonatal Screening* 2021;**7**:. https://doi.org/10.3390/ijns7030060.

592 Chen HF, Rose AM, Waisbren S, Ahmad A, Prosser LA. Newborn Screening and Treatment of Phenylketonuria: Projected Health Outcomes and Cost-Effectiveness. *Children (Basel)* 2021;**8**:. https://doi.org/10.3390/children8050381.

593 Susich M, Hersh AR, Greiner K, Chaiken SR, Caughey AB. A cost-effectiveness analysis of universal hepatitis C screening in all United States pregnancies. *Journal of Maternal-Fetal and Neonatal Medicine* 2021:1–8. https://doi.org/10.1080/14767058.2021.1949442.

594 Seror V, Leruez-Ville M, Zek A, Ville Y. Leaning towards Cytomegalovirus serological screening in pregnancy to prevent congenital infection: a cost-effectiveness perspective. *BJOG: An International Journal of Obstetrics & Gynaecology* 2021. https://doi.org/10.1111/1471-0528.16966.

595 Beilby H, Yang F, Gannon B, McIntyre HD. Cost-effectiveness of gestational diabetes screening including prevention of type 2 diabetes: application of the GeDiForCE model in Australia. *Journal of Maternal-Fetal and Neonatal Medicine* 2021:1–8. https://doi.org/10.1080/14767058.2021.1973415.

596 Smith GC, Moraitis AA, Wastlund D, Thornton JG, Papageorghiou A, Sanders J, *et al.* Universal late pregnancy ultrasound screening to predict adverse outcomes in nulliparous women: a systematic review and cost-effectiveness analysis. *Health Technology Assessment* 2021;**25**:1–190. https://doi.org/10.3310/hta25150.

597 Dubon Garcia A, Devlieger R, Redekop K, Vandeweyer K, Verlohren S, Poon LC. Cost-utility of a first-trimester screening strategy versus the standard of care for nulliparous women to prevent pre-term pre-eclampsia in Belgium. *Pregnancy Hypertension* 2021;**25**:219–24. https://doi.org/10.1016/j.preghy.2021.06.012.

598 Shih ST, Farrar MA, Wiley V, Chambers G. Newborn screening for spinal muscular atrophy with disease-modifying therapies: a cost-effectiveness analysis. *Journal of Neurology, Neurosurgery, and Psychiatry* 2021;**92**:1296–304. https://doi.org/10.1136/jnnp-2021-326344.

599 Sharma Y; Sinha, K; Ching, TYC; Marnane, V; Gold, L; Wake, M; Wang, J; Parkinson, B RG. An Economic Evaluation of Australia’s Newborn Hearing Screening Program: A Within-Study Cost-Effectiveness Analysis. *Ear & Hearing* 2021;**11**:.

600 Swallow CH, Harvey CN, Harmanli O, Shepherd JP. 47 Universal screening for fecal incontinence in pregnant women with a history of obstetric anal sphincter injury: A cost-effectiveness analysis. *American Journal of Obstetrics and Gynecology* 2021;**224**:S773–4. https://doi.org/10.1016/j.ajog.2021.04.072.

601 Vidavalur R, Bhutani VK. Economic evaluation of point of care universal newborn screening for glucose-6-Phosphate dehydrogenase deficiency in United States. *Journal of Maternal-Fetal and Neonatal Medicine* 2021:1–9. https://doi.org/10.1080/14767058.2021.1892067.

602 Bacheller HL, Frank ZC, Caughey AB. 855 Universal screening for vasa previa: a cost-effectiveness analysis. *American Journal of Obstetrics and Gynecology* 2021;**224**:. https://doi.org/10.1016/j.ajog.2020.12.878.

603 Sumire S. Cost-effectiveness analysis of maternal immunisation against Group B streptococcus in Japan. *Journal of Obstetrics and Gynaecology Research* 2021;**47**:2815.
